# Opioid‐specific medication‐assisted therapy and its impact on criminal justice and overdose outcomes

**DOI:** 10.1002/cl2.1215

**Published:** 2022-01-07

**Authors:** C. Clare Strange, Sarah M. Manchak, Jordan M. Hyatt, Damon M. Petrich, Alisha Desai, Cory P. Haberman

**Affiliations:** ^1^ Department of Sociology and Criminology, Criminal Justice Research Center Pennsylvania State University University Park Pennsylvania USA; ^2^ University of Cincinnati School of Criminal Justice Cincinnati Ohio USA; ^3^ Department of Criminology and Justice Studies Drexel University Philadelphia Pennsylvania USA; ^4^ Department of Psychology Drexel University Philadelphia Pennsylvania USA

## Abstract

**Background:**

The overlap between justice system involvement and drug use is well‐documented. Justice‐involved people who misuse opioids are at high risk for relapse and criminal recidivism. Criminal justice policymakers consider opioid‐specific medication‐assisted therapies (MATs) one approach for improving outcomes for this population. More research is needed that explores the impacts of opioid‐specific MATs for justice‐involved people.

**Objectives:**

This study sought to assess the effects of opioid‐specific MAT for reducing the frequency and likelihood of criminal justice and overdose outcomes for current or formerly justice‐involved individuals.

**Search Methods:**

Records were searched between May 7, 2021 and June 23, 2021. We searched a total of sixteen proprietary and open access databases that included access to gray literature and conference proceedings. The bibliographies of included studies and relevant reviews were also searched.

**Selection Criteria:**

Studies were eligible for inclusion in the review if they: (a) assessed the effects of opioid‐specific MATs on individual‐level criminal justice or overdose outcomes; included (b) a current or formerly justice‐involved sample; and (c) a randomized or strong quasi‐experimental design; and c) were published in English between January 1, 1960 and October 31, 2020.

**Data Collection and Analysis:**

We used the standard methodological procedures as expected by The Campbell Collaboration.

**Main Results:**

Twenty studies were included, representing 30,119 participants. The overall risk of bias for the experimental studies ranged from “some” to “high” and for quasi‐experimental studies ranged from “moderate” to “serious.” As such, findings must be interpreted against the backdrop of less‐than‐ideal methodological contexts. Of the 20 included studies, 16 included outcomes that were meta‐analyzed using mean log odds ratios (which were reported as mean odds ratios). Mean effects were nonsignificant for reincarceration (odds ratio [OR] = 0.93 [0.68, 1.26], SE = .16), rearrest (OR = 1.47 [0.70, 3.07], SE = 0.38), and fatal overdose (OR = 0.82 [0.56, 1.21], SE = 0.20). For nonfatal overdose, the average effect was significant (OR = 0.41 [0.18, 0.91], SE = 0.41, *p* < 0.05), suggesting that those receiving MAT had nearly 60% reduced odds of a nonfatal overdose.

**Implications for Policy, Practice, and Research:**

The current review supports some utility for adopting MAT for the treatment of justice‐involved people with opioid addiction, however, more studies that employ rigorous methodologies are needed. Researchers should work with agencies to improve adherence to medication regimens, study design, and collect more detailed information on participants, their criminal and substance use histories, onset, and severity. This would help clarify whether treatment and control groups are indeed comparable and provide better insight into the potential reasons for participant dropout, treatment failure, and the occurrence of recidivism or overdose. Outcomes should be assessed in multiple ways, if possible (e.g., self‐report and official record), as reliance on official data alone may undercount participants' degree of criminal involvement.

## 1 PLAIN LANGUAGE SUMMARY

### 1.1 Opioid‐specific medication assisted therapy reduces non‐fatal overdoses for justice‐involved people, but not fatal overdose or criminal justice outcomes

Opioid‐specific medication assisted therapy (MAT) significantly reduces the odds that justice‐involved people will experience non‐fatal overdoses. MAT does not appear to reduce the odds of criminal justice outcomes (such as offending, rearrest, reconviction, reincarceration) or fatal overdose, despite some evidence of this in individual studies.

### 1.2 What is this review about?

There is much overlap between people who misuse opioids and those with justice system involvement. Addiction is shown to increase the likelihood of continued justice system involvement and overdose. Criminal justice agencies are therefore under pressure to treat addiction, and MAT is one promising approach specific to opioids.

This Campbell systematic review considers the impacts of opioid‐specific MAT on rearrest, reconviction, reincarceration, and offending, as well as fatal and non‐fatal overdose for justice‐involved populations.

### 1.3 What is the aim of this review?

This Campbell systematic review assesses the effects of opioid‐specific medication assisted therapy (MAT) for reducing the frequency and likelihood of criminal justice and overdose outcomes for current or formerly justice‐involved individuals.

### 1.4 What studies are included?

This review includes 20 studies, of which 14 are experimental and six are quasi‐experimental. Though there is some degree of methodological concern across all studies, 16 studies were of sufficient methodological quality, rigour and similarity (e.g., measurement of outcomes) to be included in the meta‐analysis.

The studies span the years 1999 to 2021, and were carried out in the USA, Canada, Australia, the UK and Norway.

### 1.5 What are the main findings of this review?

There is a significant reduction in the odds of non‐fatal overdose for justice‐involved people who are treated with MAT. However, there are no significant reductions in the odds of offending, rearrest, reconviction, reincarceration or fatal overdose.

These findings are tempered by evidence of poor adherence to study designs and medication regimens.

### 1.6 What do the findings of the review mean?

MAT may be useful to agencies that serve justice‐involved people, in conjunction with interventions that target other causes of criminal behaviour and that deliver comprehensive evidence‐based substance use treatment services beyond medication.

More studies are needed that include strong research designs such as random assignment to treatment or control groups.

Researchers should work with agencies to improve adherence to medication regimens, study design, and to collect more detailed information on study participants, including their demographic information, treatment and criminal histories, medication adherence, and symptom onset and severity. Outcomes should be assessed in multiple ways across the full follow‐up period, including from self‐reported and official records.

### 1.7 How up‐to‐date is this review?

The review authors searched for studies between May and June 2021.

## 2 BACKGROUND

### 2.1 Description of the problem

Opioids have become increasingly available worldwide and pose some of the most serious health consequences as compared to other types of drugs (United Nations Office on Drugs & Crime [UNODC], 2015). In 2018, 58 million people used opioids worldwide. Over two‐thirds of deaths due to drug use are from opioids, and in 2017, over 115,000 died from an opioid overdose (World Health Organization [WHO], 2020).

The overlap between criminal justice system involvement and drug use is well‐documented across a variety of countries and samples (e.g., Boutwell et al., 2007; Dolan et al., 2007; European Monitoring Centre on Drugs and Drug Addiction [EMCDDA], 2012; Winkelman et al., 2018). Once released from secure correctional facilities, people with opioid addiction are at high risk for relapse and criminal recidivism. Specifically, a meaningful minority of deaths of former inmates is attributable to opioid overdose (Binswanger et al., 2013; Singleton et al., 2016; World Health Organization [WHO], 2010), and a significant percentage of former inmates will recidivate within five years (Fazel & Wolf, 2015).

Criminal justice agencies have been particularly overwhelmed by the recent opioid epidemic. Treating opioid (and other substance) addiction as a means to reduce risk for future criminality and improve public safety is inherently a responsibility for the criminal justice system, as the influence of substance use on criminal activity is well documented in the literature (Bonta & Andrews, 2017). Of course, one could also argue that opioid addiction, its withdrawal symptoms, and its recovery constitute a serious medical condition for which criminal justice agencies have a responsibility to treat and manage, in accordance with the United Nations requirements for the Basic Principles for the Treatment of Prisoners (United Nations, 1990). In fulfilling their responsibility to provide adequate health care for individuals in their custody, and through their efforts to rehabilitate offenders to reduce their risk for recidivism more generally, correctional providers must treat substance use disorders. When they do, they may impact criminal recidivism as well as health outcomes like future overdose. As such, it is necessary to deploy the most effective treatment available to achieve maximum impact on these outcomes.

Policy recommendations (WHO, 2009) place emphasis on the use of medication‐assisted treatments (MAT) as a front‐line defense among correctional populations, because its efficacy and effectiveness has been well‐established in other contexts (Belenko et al., 2013; Koehler et al., 2014). Despite these policy recommendations criminal justice agencies have been reluctant or slow to do so (Friedmann et al., 2012; Matusow et al., 2013; Parrino et al., 2015). Many factors may contribute to the poor uptake of this particular approach for managing and treating opioid addiction. It is possible that practitioners may question the utility for MAT to impact public safety outcomes—the chief policy concern of the criminal justice system. Indeed, the uptake of psychological research evidence—particularly that which establishes a strong link between addiction and criminality—into correctional policy and practice has been slow, at best (Gannon & Ward, 2014). Moreover, there may be confusion or even hesitation among practitioners in correctional settings about their responsibility to encourage or administer an intervention that traditionally falls under the purview of health care providers.

### 2.2 Description of the intervention

There are a variety of MAT drugs that are currently used for the management and treatment of opioid addiction. This review focuses on those most modern and commonly used drugs to treat opioid addiction over the long‐term, in the form of supervised maintenance programs, drug substitution, or antagonist protocols. Thus, this review does not examine the effects of Naloxone, which is used to revive someone in a singular emergent opioid overdose event.

The drugs examined in the current review include opioid agonists (heroin, methadone, and levo‐alpha‐acetyl‐methadol), partial agonists (buprenorphine), and antagonists (naltrexone). Opioid agonists are drugs that work on the opioid receptors in the brain and produce a full opioid effect. Heroin and methadone maintenance MAT services must be administered under the supervision of medical professionals in a highly controlled environment and on a regimented schedule. This approach is designed to help reduce illicit or off‐label use of opioids, cravings, and, gradually, the amount of opioid intake over time. Partial agonists like buprenorphine also operate on the opioid receptors but produce weaker euphoric effects than felt with full agonists. This class of MATs is also designed to help lower dependency symptoms, misuse, cravings, and symptoms of withdrawal. Buprenorphine is a longer‐acting agent, so it can be administered less frequently and has approval to be administered in a variety of clinical settings. Opioid antagonists like naltrexone block the opioid receptors entirely, so that if a patient used an opioid, they will not be able to achieve any euphoric effects. It is designed to relieve withdrawal and cravings and must be administered by a doctor, nurse, or nurse practitioner.

### 2.3 How the intervention might work

The impact of opioid‐specific MAT on overdose outcomes is well established. MAT reduces cravings, illicit drug use, and the amount of opioid use over time (Belenko et al., 2013; Koehler et al., 2014). All of these, in turn, aid in the reduction of overdose outcomes. The mechanisms by which opioid‐specific MAT impact criminal justice outcomes are less understood. However, prior research on substance use treatment in general and correctional rehabilitation theory suggests MAT could reduce criminal risk. As substance use is a robust predictor of criminal involvement, reducing substance use may reduce future criminal involvement (see Bonta & Andrews, 2017). By extension, any intervention targeting addiction, including MAT, may operate to reduce recidivism risk. More specifically, because MAT facilitates reductions in risky drug use, opioid users may engage less frequently or not at all in drug‐related behaviors that warrant a criminal justice response (i.e., drug use, possession, trafficking, paraphernalia possession). Similarly, by reducing cravings and use, people may no longer be motivated to engage in criminal activity that supports, or fuels, their addiction (e.g., burglary).

### 2.4 Why it is important to do this review

The current evidence base for MAT on overdose outcomes is strong, but little is known about its impact on the subsample of people with an opioid addiction who also are involved with the criminal justice system. Because these individuals face challenges posed by addiction and criminal justice involvement, they likely have different experiences, needs, and risks than people not facing this combination of challenges. Thus, it is necessary to identify whether the same positive clinical outcomes seen among non‐offender or mixed groups can be observed among people with current or prior criminal justice involvement. Further, although addressing substance use should reduce criminal risk (Bonta & Andrews, 2017), it is unclear if it is enough to reduce recidivism for people with a serious opioid addiction. A rigorous and systematic synthesis of the evidence base on the effectiveness of MAT for improving public safety will allow criminal justice agencies to make informed decisions about policy, practice, and the allocation of resources. In light of the range of MAT options currently available and the pressing need for methodologically robust results and changes to the underling legal and public health landscape, an updated and complete review is particularly policy relevant today.

This systematic review is an update and modification of a 2009 Campbell Systematic Review entitled “Effects of Drug Substitution Programs on Offending among Drug‐Addicts” (Egli et al., 2009). Although the authors of this review reported the intent to publish an update every five years, no update has yet been published. To the current authors' knowledge, an update is also not currently in progress or planned. As ten years of research has amassed on this topic, particularly during the height of the “opioid epidemic” and with the application of newer MAT therapies in opioid treatment (e.g., naltrexone), it is necessary to update this 2009 review. Further, this review is more comprehensive, because it includes both criminal justice and overdose outcomes observed among exclusive criminal justice samples and incorporates studies examining a variety of pharmacological interventions for opioid use.

### 2.5 Objectives

The current review provides criminal justice and substance use treatment decision‐makers with information regarding the efficacy and effectiveness of opioid‐specific medication‐assisted therapies (MAT) on offending and overdose outcomes. Specifically, the authors address the following objectives:

1. To assess the effects of opioid‐specific MATs for reducing the frequency or likelihood of criminal justice outcomes (as defined by official or self‐reported indices of offending, arrest, conviction, or incarceration) for individuals currently or previously involved in the criminal justice system; and
2. To assess the effects of opioid‐specific MATs for reducing the frequency or likelihood of opioid overdose for individuals currently or previously involved in the criminal justice system.

The objectives will help to inform criminal justice and substance use treatment policymakers on the usefulness of opioid‐specific MATs in reducing criminal justice outcomes in criminal justice settings, or overdose outcomes in treatment settings with the criminal justice population. Implementing or maintaining an opioid‐specific MAT program is a practical decision that requires the use of program resources, and therefore it is important that program leadership have a foundational understanding of the intervention and its established efficacy and effectiveness.

## 3 METHODS

The current review is an update and expansion of a Campbell Collaboration publication “Effects of Drug Substitution Programs on Offending among Drug‐Addicts” (Egli et al., 2009). The associated protocol can be found at https://onlinelibrary.wiley.com/doi/full/10.1002/cl2.1138 (Strange et al., 2021).

### 3.1 Criteria for considering studies for this review

#### 3.1.1 Types of studies

To be eligible for inclusion in this review, studies were required to use a strong quasi‐experimental or randomized experimental design that prospectively tests the effects of the MAT for opioid use disorder on criminal justice and overdose outcomes. Due to the difficulty of conducting randomized controlled trials (RCTs) in criminal justice settings, it is necessary to examine quasi‐experimental studies that employ more rigorous design features. Specifically, all quasi‐experimental studies were required to either use a matching procedure when testing differences in the treatment and comparison groups or use statistical controls for baseline group differences (if observed). This was necessary to ensure equivalent comparison groups. All studies were required to use an individual level unit of analysis.

#### 3.1.2 Types of participants

Study samples were required to consist of opioid‐using adults and adolescents who are male, female, or nonbinary, and racially/ethnically diverse. All participants had to have current opioid use as indicated by self‐report or diagnosis; participants were not required to have an opioid‐specific substance use disorder (OUD) but were likely to, given opioid‐specific MAT is typically administered for people with a known diagnosis of OUD. Additionally, all participants in the study samples had to have current prior criminal justice involvement, as indicated by self‐report or official report of prior or current arrest, incarceration, charges, or convictions. This review did not include studies that examined samples with no current or prior criminal justice involvement, or if the sample was mixed with respect to criminal justice involvement, or if this information was missing from the manuscript.

#### 3.1.3 Types of interventions

In contrast to the original review, which included MAT treatment for other illicit substance use (e.g., cocaine), this review focused solely on MAT for opioid use disorder. Specifically, this review included studies that tested the impacts of heroin and methadone maintenance, buprenorphine, levo‐alpha‐acetyl‐methadol, and/or naltrexone as the independent variable. The comparison and control group for the quasi‐experimental and experimental designs, respectively, could be any intervention that was not an opioid‐specific MAT (i.e., alternative medication not specifically intended for opioid use treatment [e.g., anti‐depressant]), “talk therapy” (i.e., any individual or group counseling, using any theoretical model or approach; e.g., cognitive behavioral therapy, group processing, psychotherapy), no intervention, forced detoxification, wait list control, or a placebo. Additionally, the review also allowed for comparison of two opioid‐specific MAT conditions (e.g., methadone vs. buprenorphine), as well as combined MAT + talk therapy versus a comparison condition fitting the above criteria. We did not impose restrictions on the number of treatment versus control conditions, but because biomedical or pharmaceutical research with criminal justice populations can be logistically challenging, we did not anticipate many studies with multiple conditions.

The current review included studies of opioid‐specific MAT meeting the inclusion criteria, regardless of where it was administered or delivered (e.g., community, court, institutional). This was an expansion upon the original review, which excluded incarceration‐based treatment programs.

#### 3.1.4 Types of outcomes

The primary dependent variable, criminal justice involvement, was determined through self‐report or official record, and could include any of the following outcomes: reconviction, rearrest, reincarceration, or reoffending. Outcomes could be in the form of failure proportions, mean frequencies, or survival rates.

The secondary dependent variable examined was opioid overdose, which could also be determined through self‐report (nonfatal overdose) or official record (nonfatal and fatal overdose). Nonfatal outcomes could be in the form of mean frequencies, and both overdose outcomes could be in the form of failure proportions or survival rates.

### 3.2 Search methods for identification of studies

#### 3.2.1 Electronic searches

The original review included relevant studies identified through databases such as Campbell Crime and Justice Group, National Criminal Justice Reference Service, MEDLINE, National Treatment Agency for Substance Misuse, National Treatment Outcome Research Study, Central Committee on the Treatment of Heroin Addicts, Criminal Justice Abstracts, and JSTOR. The current review considered all studies from the Egli et al. (2009) review, in addition to studies independently selected by the authors of the current review. For criminal justice outcomes the authors considered all studies published between January 1, 2007 and October 31, 2020 as this portion of the review was an update of the Egli et al. (2009) study. For overdose outcomes the authors considered any study published between January 1, 1960 through October 31, 2020. Thus, any studies published on or after November 1, 2020 were not included in this review. In contrast to the original review, only studies published in English were considered for the current review, as the author team did not have sufficient bi‐ or multi‐lingual individuals to have two independent coders per study in another language. All searches were conducted by the first author between May 7, 2021 and June 23, 2021 (see Supporting Information Appendix 1 for database‐specific dates).

The studies for the current review were accessed on the following platforms (via access from the University of Cincinnati), followed by the specific databases and dates of coverage in parentheticals: EBSCOhost (Criminal Justice Abstracts [1910‐present], SocINDEX with Full Text [1895‐present], Legal Collection [1965‐present], Wilson Omnifile [1980‐present], PsycINFO [1872‐present], Social Work Abstracts [1965‐present], MEDLINE [1781‐present for citations and 1965‐present for full text], and Women's Studies International [1972‐present, included gray literature]); ProQuest (Criminal Justice Database [1937‐present], PAIS [1914‐present, included gray literature], Dissertations & Theses Global [1961‐present, included gray literature]); ISI Web of Knowledge (Web of Science Core Collection [1900‐present]). The following open access platforms and databases were also consulted: Elsevier (Scopus [1966‐present, includes gray literature], elsevier.com/solutions/scopus); National Institute of Justice Crime Solutions (crimesolutions.ojp.gov [2011‐present]); Cochrane Central Register of Controlled Trials (CENTRAL) (www.cochranelibrary.com/central [1908‐present]); and Google Scholar (scholar.google.com, for forward citation searching of included articles only [dates of coverage unknown]). These databases were identified as common databases used in criminal justice research, at the expertise of several general and criminal justice‐specific librarians at the University of Cincinnati and include such important unpublished sources as conference papers and gray literature.

#### 3.2.2 Searching other sources

Furthermore, and similar to Egli et al. (2009), the authors of the current review consulted the bibliographies of other relevant reviews for additional studies to include, as well as the bibliographies of the included studies. Irrespective of electronic availability, the authors contacted university libraries and first/corresponding authors to retrieve all articles that appeared to meet the criteria for inclusion.

For the current review, search terms were harvested according to their demonstrated success in drawing out relevant and complete results for studies regarding the effectiveness of opioid‐specific MAT. This method was adapted from the rigorous strategies often employed in systematic reviews from the medical field. First, 10 “gold‐standard” articles were selected from the Egli et al. (2009) review (i.e., those studies that best reflected the type of studies desired for the current review, both methodologically and in subject matter). These articles were entered into the PubMed database, where a Medical Subject Heading (MeSH) analysis generated a list of common terms across all ten gold‐standard articles. The author team identified relevant terms from the MeSH analysis and then brainstormed potential variants of each term and Boolean operators (including variants of the terminology, spelling, use of quotations, and truncations) to determine the version of each term that was most likely to draw complete and relevant results. Each term was tested using the Criminal Justice Abstracts database for its breadth of subject matter. From this process two core search strings were created, each with the same general base terms, but unique outcome measure(s) (i.e., the specified criminal justice or overdose outcomes). Search strings were created such that studies were retrieved if they contained any of the base terms, *and* the outcome. Some search strings were modified due to database functionality. All final search strings are listed by platform and database in Supporting Information Appendix 1. Results from all source types were considered in the initial phase of the search (e.g., newspapers, journals, letters, conference abstracts) unless otherwise indicated in Supporting  Information Appendix 1 (due to issues with volume and relevance of results).

### 3.3 Data collection and analysis

#### 3.3.1 Description of methods used in primary research

The studies included in the current review employed an experimental or strong quasi‐experimental design and measured the impacts of the specified MATs on individual‐level criminal and overdose outcomes for people with opioid use problems who are currently or previously justice system‐involved.

In the quasi‐experimental and experimental studies, the treatment group could have received an opioid‐specific MAT (e.g., buprenorphine, naltrexone, methadone maintenance, heroin maintenance, levo‐alpha‐acetyl‐methadol), and the control group could have received a different type of opioid‐specific MAT (e.g., methadone compared to buprenorphine), a placebo, some sort of alternative medication not specific to opioid addiction, talk therapy (e.g., individual or group counseling), or no treatment at all. Additionally, the treatment condition could also have been a MAT + talk therapy treatment. Coders attempted to subclassify all talk therapy interventions into cognitive‐behavioral (CBT) versus other, since cognitive‐behavioral therapies traditionally produce greater effects and have a larger evidence base than other approaches in the treatment of substance use disorder (McHugh et al., 2010). However, the descriptions of the psychosocial/talk therapy interventions lacked this level of specificity in the original articles to support this level of detail in coding.

#### 3.3.2 Criteria for determination of independent findings

Egli et al. (2009) discussed three potential avenues for the nonindependence of findings: (1) multiple indicators of offending reported from a single study (e.g., arrest, criminal offending); (2) the same outcome measured at multiple points in time; and (3) the same data being reported across multiple studies. The criteria for the determination of independent findings are the same for the current review as is standard in Campbell Review protocols (see e.g., Lipsey & Landenberger, 2006).

Four primary, potentially correlated indicators of criminal involvement were examined: (1) rearrest; (2) reincarceration; (3) reconviction; and (4) reoffending. Upon completion of coding, only two of these outcomes were consistently measured across multiple studies, lending themselves to meta‐analysis: reincarceration and rearrest. Studies that reported the other criminal justice outcomes that were not included in the meta‐analysis are discussed in narrative format (e.g., Bellin et al., 1999) so as not to preclude them from contributing information to the review. Given that only four studies reported both reincarceration and rearrest outcomes, and because these are unique outcomes that are often correlated in the literature but not necessarily interdependent, these four studies are meta‐analyzed in each criminal justice outcome. Importantly, no one study is represented twice within an analysis. Following a similar logic, nonfatal and fatal overdoses are meta‐analyzed separately and include studies that report both outcomes. For studies that reported outcomes at multiple points, the outcome with the longest‐follow up or with the follow up most similar to that used across the other studies was coded—typically six or 12 months. This was done to encourage as much comparability as possible given the unique methods employed across some studies (Lipsey & Landenberger, 2006).

In the event that multiple publications reported results using the same set of data, the study with the most complete and detailed outcome information was used as the primary coding source. Following the study coding protocol, coders also referenced published study protocols (e.g., clinical trial registrations) and affiliated publications to ensure accurate and complete coding of study methodologies and findings. A list of all reports of the included studies (i.e., study “families”) can be found in Supporting Information Appendix 2.

In addition to the above avenues for the potential nonindependence of findings, it is also possible that multi‐arm studies will include more than one eligible comparator condition. For these studies the authors combined MAT and comparator conditions so that only a single pairwise comparison was computed. This is in line with recommendations from Higgins, Eldridge, et al. (2019) and prevents an intervention group from being double counted.

#### 3.3.3 Selection of studies

Once a full set of potentially relevant citations were identified, the authors received assistance from a Campbell Collaboration representative to de‐duplicate the results using EndNote®. After de‐duplication, all remaining citations were uploaded to DistillerSR® systematic review software. Three members of the author team and six students trained by the study authors independently reviewed all potentially relevant studies for the proper inclusion criteria. All studies were screened in two phases. In the first phase, the titles and abstracts were reviewed to determine if basic inclusion criteria appeared to be met—that is, (a) the experimental or strong quasi‐experimental evaluation of effectiveness of MAT services (b) on criminal or overdose outcomes (c) for people with opioid use disorder (d) who are or have been involved in the criminal justice system. Studies meeting these criteria, or any study for which this information could not be readily determined from the title or abstract, were retained for screening in Phase 2.

**Figure 1:**
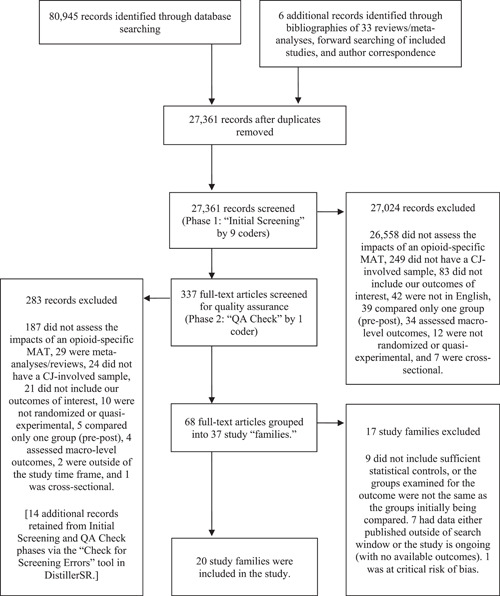
PRISMA flow chart for included studies

In Phase 2, the full text of each study was reviewed by the second author. All studies with inappropriate design and/or rigor, irrelevant independent or dependent variables, and ineligible sample characteristics were removed from consideration for inclusion. Reviews and meta‐analyses were also removed from inclusion but flagged so that the coding team could later review their reference lists for studies that should be included in the current review but were not identified through the initial search. As a check to ensure relevant studies were not mistakenly excluded at either phase, the “Check for Screening Errors” function in the DistillerSR® software was employed for all excluded studies that went through both phases of review and, separately, for all studies excluded at Phase 1. This software feature uses machine learning to identify potentially misclassified studies based upon characteristics of the studies included. The second author re‐reviewed in detail 382 total citations identified by the software and added back in 14 citations mistakenly excluded at earlier phases of review. Figure 1 contains the PRISMA flow chart.

#### 3.3.4 Data extraction and management

The Egli et al. (2009) team created a coding protocol for the original review that provided a systematic method of extracting information regarding each study's research design, program, nature of the outcome measures, and outcome data. This protocol was availed to the study authors to promote consistency in the coding procedures. The current study team updated the protocol to reflect the changes from the original to the current review. The updated coding protocol included the systematic extraction of information regarding the study identification, content and methodological inclusion criteria and rigor, control and treatment sample descriptive information, actions taken upon the control and treatment samples, treatment characteristics, the types and measurement of outcome data, and effect size information (see Supporting Information Appendix 3 for the updated coding protocol).

A team of eight Ph.D. and doctoral‐level coders were trained on the updated coding protocol and two coders coded each study independently. If discrepancies were observed, a third coder not originally assigned to that study resolved the discrepancy. Discrepancies between the coders were quite rare and were often the result of coding information in the wrong place, as opposed to coding the information incorrectly.

#### 3.3.5 Assessment of risk‐of‐bias for included studies

We used two tools to assess risk‐of‐bias in our included studies: (1) The Revised Cochrane risk‐of‐bias tool for randomized trials (RoB 2; Higgins, Savović, et al., 2019; see Supporting Information Appendix 7); and (2) The Risk of Bias in Non‐randomized Studies—of Interventions (ROBINS‐I; Sterne et al., 2016; see Supporting Information Appendix 8) assessment tool. The RoB tool guides coders in rating five total domains: (1) Bias arising from the randomization process; (2) Bias due to deviations from intended interventions; (3) Bias due to missing outcome data; (4) Bias in the measurement of the outcome; and (5) Bias in the selection of reported results. Each domain is rated on a scale from one to three, where one = low risk of bias, two = some concerns, and three = high risk of bias. Risk of bias for randomized trials “should be expressed only about issues that are likely to affect the ability to draw reliable conclusions from the study” (Higgins, Savović, et al., 2019, p. 4). Coders follow the RoB manual to answer specific “signaling questions” posed in each domain rated. Coders follow the instructions based on the answers to these signaling questions to yield specific overall judgments of risk in each domain. The manual provides specific definitions of low, some, and high risk for each domain, which further helps coders to reliably rate the domain. The overall risk of bias for the whole study must be at least the level of the domain rated as highest risk (e.g., if one domain is rated as some concern [two] and all others are rated as low risk [one], the study must be rated as some concern [two]). Additionally, if multiple domains are rated as some concern [two], but none are rated as high concern individually, the study's overall rating could be either some [two] or high [three] risk, depending on the extent of the issues within and across domains.

For the ROBINS‐I tool, coders assessed bias across seven domains: (1) Bias due to confounding; (2) Bias in selection of participants into the study; (3) Bias in classification of the interventions; (4) Bias due to deviations from intended interventions; (5) Bias due to missing data; (6) Bias in measurement of the outcomes; and (7) Bias in the selection of reported results. Each domain is scored on a 1–5 scale, where 1 = *low risk of bias* (“comparable to a well‐performed randomized trial with regard to this domain”), 2 = *moderate risk of bias* (“study is sound for a non‐randomized study with regard to this domain but cannot be considered comparable to a well‐performed randomized trial”), 3 = *serious risk of bias* (“the study has some important problems in this domain”), 4 = *critical risk of bias* (“the study is too problematic in this domain to provide any useful evidence on effects of the intervention and should not be included in any synthesis”), and 5 = *no information* (“no information on which to base a judgment about risk of bias for this domain”). Like the RoB, the ROBINS‐I tool provides signaling questions and detailed guidance to coders in how to render an overall rating of risk of bias for each domain. Also like the RoB, the overall rating for the study must be at least equal to the highest score (one through four) in any one domain. Studies can be rated with an overall risk of five (no information) only if one or more domains is rated as no information [five] and the other domains are rated as low risk [one].

Two of the study authors read both the RoB and the ROBINS‐I manuals and referenced them frequently while completing the ratings of risk of bias for each study. The two coders independently rated each domain for each study first. Scores were then compared and the coders discussed any domains for which there was disagreement and arrived at an overall risk of bias rating for each study, consistent with each tool's scoring guidelines. The percent agreement in initial coding (i.e., pre‐discussion/consensus), plus the overall judgment of risk of bias for each study, and the rationale supporting these determinations are reported for experimental studies in Supporting Information Appendix 4 and quasi‐experimental studies in Supporting Information Appendix 5.

#### 3.3.6 Measures of treatment effects

The statistical procedures and conventions align closely with those that were used in the Egli et al. (2009) review, as the types of studies and outcomes that were included are similar. The most detailed numerical data were coded to facilitate similar analyses across the included studies. For binary offending outcomes (e.g., arrest, conviction, incarceration, and criminal involvement) and overdose outcomes (fatal and nonfatal), odds ratios were computed for the individual studies and mean logged odds ratios were used in the meta‐analyses. We exponentiated and inverted the mean logged odds ratios and reported these in the tables, forest plots, and text to show a positive mean treatment effect (i.e., an odds ratio [OR] < 1 indicates a reduction in the outcome). Continuous or quasi‐continuous measures of these outcomes (e.g., average number of arrests) were rarely and inconsistently reported across studies and therefore were not meta‐analyzed.

#### 3.3.7 Dealing with missing data

One study (Bellin et al., 1999) did not report the necessary data to allow its inclusion in the meta‐analysis. Despite successful contact and correspondence with the lead author, these data were no longer available or on record and, as such, the study could not be included in the meta‐analysis.

#### 3.3.8 Assessment of heterogeneity

To assess heterogeneity, we used the homogeneity *Q* test. A *p*‐value of 0.10 was set as the cut off for significance as higher quality studies are likely to have smaller sample sizes, which may reduce the statistical power of the *Q* test and increase the likelihood of a type II error.

#### 3.3.9 Data and analysis

The current review complies with the standards of meta‐analysis as specified in *Practical Meta‐Analysis* by Lipsey and Wilson (2001). The two types of included studies (RCTs and quasi‐experiments) were meta‐analyzed together using SPSS v.28 (IBM Corp., 2021). As stated above, in multi‐arm studies (i.e., in which there was more than one eligible comparator condition) the authors combined the intervention and comparator conditions so that only a single pairwise comparison was computed. This prevents an intervention group from being “double counted” and inflating the unit of analysis error (Higgins, Savović, et al., 2019). This affected six total studies, for which two of the authors independently classified the comparator arms into either a treatment or control groups based upon both the similarity of the intervention received and the timing of the condition. The two raters had 100% agreement on these classifications. Study arms were combined in the following manner: (1) in Farabee et al. (2020), the naltrexone and naltrexone + patient navigation groups were combined into one MAT condition; (2) in Farrell‐MacDonald et al. (2014), the methadone continued and methadone discontinued groups were combined into one MAT condition; (3) in Kinlock et al. (2005), the LAAM group was combined with the LAAM discontinued group into one MAT condition; (4) in Gordon et al. (2008), the passive and active referral groups were combined into one no‐MAT comparator condition; (5) in MacSwain et al. (2014), the no treatment and wait list groups were combined into one no‐MAT comparator condition; and (6) in McKenzie et al. (2012), the referral and referral + financial assistance were combined into one no‐MAT comparator condition.

To compute mean effect sizes (i.e., log odds ratios) the inverse variance weight method of the meta‐analysis was used (Lipsey & Wilson, 2001) and random effects models were assumed a priori. Fixed effects models were conducted first to examine any heterogeneity of effects due to sample size. There was no significant evidence of funnel plot asymmetry and, as such, results from random effects models are reported. No effect size outliers were observed, and no data were imputed for missing values.

##### 3.3.9.1 Subgroups

The potential moderators of MAT effectiveness on criminal justice and overdose outcomes determined a priori included: (1) study design elements (i.e., experimental, quasi‐experimental, follow‐up period); and (2) treatment elements (e.g., supplementing MAT with individual or group CBT or non‐CBT counseling, medication dosage and adherence, treatment length). Secondary a priori potential moderators included gender, race, and age of the sample, location/context of treatment (e.g., jail, prison, community, court), and era (i.e., before or during the opioid epidemic). Upon completion of coding, we were only able to empirically examine the effects of study design type (experimental vs. quasi‐experimental) on the outcome. The other potential moderators were either missing, inconsistently reported across the studies, or there was not enough variability across the studies that reported the variable and measured it consistently.

#### 3.3.10 Sensitivity analysis

Relative to the quasi‐experimental studies, the experimental studies employed smaller sample sizes and, in many cases, were underpowered. As such, we assessed the impacts of sample size using meta‐regression, in which a covariate for sample size was included in the initial models. This was nonsignificant in the analyses of all four outcomes (reincarceration, rearrest, fatal overdose, and nonfatal overdose).

#### 3.3.11 Treatment of qualitative research

Qualitative research was not eligible for inclusion in this review.

## 4 RESULTS

### 4.1 Description of studies

#### 4.1.1 Results of the search

Upon completion of the initial search, 80,945 total citations were identified. After de‐duplication, 27, 361 citations remained and were subsequently screened for inclusion. In Phase 1 of screening, 27,024 citations were excluded for not meeting eligibility/inclusion criteria. Thus, 337 citations were screened at Phase 2 (quality assurance phase). At the completion of this phase, 68 citations were retained for inclusion and subsequently grouped by study, yielding 21 total studies. One study was removed at the risk of bias phase (details reported below), so 20 total studies are included in the review (of which 16 are meta‐analyzed). Details about decision‐making at each phase are available in Figure 1.

#### 4.1.2 Included studies

Of the 20 studies included in this review, 18 were peer reviewed publications, one was a student thesis, and one was an agency report. There were six quasi‐experimental studies and fourteen experimental studies. All quasi‐experimental studies examined the effectiveness of methadone, though one study (Marsden et al., 2017) had participants who were “MAT‐exposed” and could have had methadone or buprenorphine. Among the experimental studies, most assessed the effectiveness of methadone (*n* = 7), or naltrexone (*n* = 6), followed by buprenorphine (*n* = 2), and LAAM (*n* = 1).^1^ Figure 1 shows the study selection process and Table 1 shows the characteristics of the included studies (Supporting Information Appendix 2 is a supplement to Table 1, which contains all reports, that is, “study families,” for each included study).

**Table 1:** Characteristics of included studies

Citation [Study Dates]	Sample Size^a^ n (Tx) n (control) n (Tx2)		Tx Sample Character‐istics^b^		Tx group dosage, frequency and length^c^	Setting/timing of initial Tx group dose			Outcomes examined (source)	Length of follow‐up	Study location	Funding sources and declarations of interest (DOI)^d^
*Experimental studies*												
Brinkley‐Rubinstein et al. (2018) [2010–2013]	128 (MMT) 51 (DTX)	*M* age = 32; 77% male; 79% White; 4% Black; 15% Lat.; 17% Other			As clinically indicated according to dose before jail/prison	Jail/prison (continued from community)			% Rearrested % Reincarcerated % nonfatal overdose (self‐report and official)	12 months	Rhode Island, USA	National Institutes of Health (NIH) No DOIs
Cornish et al. (1997) [Dates not reported]	34 (ONX) 17 (COUN)	*M* age = 39; 90% male; 24% White; 62% Black; 14% Lat.			100 mg on Tues. and 150 mg on Fri. by study staff for 6 months	Community (Study office across the hall from probation office)			% Reincarcerated for probation violations	6 months	Philadelphia, Pennsylvania, USA	National Institute on Drug Abuse (NIDA) DuPont Pharmaceuticals No DOIs
Coviello et al. (2010) [Dates not reported]	56 (ONX) 55 (COUN)	*M* age = 33; 82% male; 54% White; 20% Black; 27% Lat.			150 mg^e^ 2×/week for *M* 3.4 months	Community (University of Pennsylvania research office)			*M* # Reconvictions *M* # Months Incarcerated *M* # Charges % Drug Arrest % Parole Violation Arrest (official and self‐report)	6 months	Philadelphia, Pennsylvania, USA	NIDA No DOIs
Dolan et al. (2005) [1997–2002]	129 (MMT) 129 (WLC)	Not separated by group.			*M* 61 mg for *M* 4.8 mos.	Prison and community			% Reincarcerated (official)	48 months	New South Wales, Australia	Several funding sources^f^ No DOIs
Farabee et al. (2020) [2015–2019]	53 (INX) 48 (NO) 50 (INX‐PN)	*M* age = 31; 76% male; 78% White; 7% Black; 65% Lat.; 15% Other			380 mg every 4 weeks for 6 months	Jail (before release)			% Rearrested (official)	12 months	Albuquerque, New Mexico, USA	NIDA Arnold Foundation Alkermes provided study medications
Gordon et al. (2018) [2008–2013]	100 (BUP) 99 (WLC)	*M* age = 40; 69% male; 29% White; 67% Black			16 mg.^g^ Delivered by study nurses	Jail			% Rearrested *M* # Rearrests *M* # Days to rearrest (official)	12 months	Baltimore, Maryland, USA	Reckitt Benckiser Pharmaceuticals, Alkermes DOIs^h^
Hyatt et al. (2021) [2015–2018]	47 (INX) 47 (WLC)	*M* age = 37; 62% male; 62% White; 19% Black; 17% Lat.			380 mg once a month for 6 months	Prison			% Reincarcerated % Rearrested (official)	6 months	Pennsylvania, USA	Pennsylvania Department of Corrections No DOIs
Kinlock et al. (2005) [2000–2001]	20 (LAAM) 31 (NO) 13(ELIG)	*M* age = 37; 100% male; 15% White; 75% Black; 10% Native American			50 mg 3× week until release, then as clinically indicated. Delivered by community program.	Prison (before release)			% Rearrested % Reincarcerated (official) *M* # Crime days (self‐report)	9 months	Baltimore, Maryland, USA	Open Society Institute No DOIs
Kinlock, Gordon, Schwartz, Fitzgerald, et al. (2009) [2003‐2008]	71 (MMT) 64 (COUN) 69 (WLC)	*M* age = 40; 100% male; 21% White; 70% Black; 9% Other			60 mg for *M* 3.3 months. Delivered by community program.	Prison (before release)			% Rearrested (official) *M* # Crime days (self‐report)	12 months	Baltimore, Maryland, USA	NIDA Reckitt Benckiser Pharmaceuticals provided the study drug DOIs^i^
Lee et al. (2016) [2008–2015]	153 (INX) 155 (COUN)	*M* age = 44; 84% male; 20% White; 53% Black; 24% Lat.			380 mg once a month for 6 months. Delivered by trial nurses/physicians.	Community			*M* # Reincarceration days % Reincarcerated (self‐report) % Fatal/nonfatal overdose (official)	6.25 months for CJ outcomes, Up to 19.5 for overdose	Pennsylvania, New York, Rhode Island, Maryland, USA	NIDA DOIs^j^
Lobmaier, Kunøe, Gossop, Katevoll, et al. (2010) [2005–2008]	23 (MMT) 21 (NTX)	Not separated by group.			*M* 90 mg for MMT, implant for NTX.	Prison (1 month before release)			% Reincarcerated (official) *M* # Days criminal activity (self‐report)	6 months	Oslo, Norway	Research Council of Norway No DOIs
Magura et al. (2009) [2006–2008]	60 (SUB) 56 (MMT)	*M* age = 38; 100% male; 25% Black; 65% Lat.			32 mg for SUB, ≥30 mg for MMT. Delivered by KEEP^k^ physicians.	Jail (at intake)			# arrests % Type of arrest (self‐report) % Reincarcerated (official)	3 months	New York, New York, USA	NIDA No DOIs
McKenzie et al. (2012) [2006–2009]	25 (MMT) 16 (NO+$) 219 (NO)	*M* age = 41; 71% male; 73% White; 21% Lat.; 6% Other; 21% NR			*M* 33 mg for 1 month. Delivered by CODAC staff.^l^	Jail/prison (1 month before release and then community referral)			% Rearrested % Reincarcerated % Fatal/nonfatal overdose (self‐report)	6 months	Rhode Island, USA	Substance Abuse and Mental Health Services Administration (SAMHSA) NIH No DOIs
Schwartz et al. (2020) [2014–2019]	69 (MMT) 72 (DTX) 71 (MMT‐PN)	Not separated by group.			60 mg for *M* 2.9 months for MMT‐PN and *M* 2.2 months for MMT. Delivered by Detention Center's Opioid Treatment Program	Jail			*M* # Crime days (self‐report)	12 months	Baltimore, Maryland, USA	NIDA Arnold Foundation Dr. Schwartz reports consulting for Verily Life Sciences, Ltd.
*Quasi‐experimental studies*												
Bellin et al. (1999)^m^ [1996–1997]	1423 (H‐MMT) 6898 (DTX) 1371 (L‐MMT)			*M* age = 39; 76% male; 21% White; 38% Black; 40% Lat.	≥60 mg for H‐MMT, ≤ 30 mg for l‐MMT. Delivered by KEEP physicians.		Jail (at intake)	*Mdn* # days to reincarceration (official)		Until reincar‐ceration or study's end (not specified)	New York, New York, USA	No DOIs
Farrell‐MacDonald et al. (2014) [2003–2011]	25 (MMT‐C) 45 (NO) 67 (MMT‐T)			*M* age = 33; 0% male; 40% Aborig.	*M* 89.1 mg (not separated by MMT group; no sig. difference between groups)		Prison	% Reincarcerated # Days on supervision (i.e., until reincarceration) (official)		27 months (max)	Canada	Canadian Institutes of Health Research (CIHR)/Public Health Agency of Canada (PHAC) Chair in Applied Public Health No DOIs
Haas (2020) [2013–2018]	660 (MMT) 904 (NO)			100% male; 43% White; 36% Black; 33% Lat.			Jail (at intake, continued from community)	% Reincarcerated % Fatal/nonfatal overdose (official)		Varied	New Haven, Bridgeport, Connecticut, USA	Centers for Disease Control and Prevention (CDC) No DOIs
MacSwain et al. (2014) [2006–2008]	161 (MMT) 214 (NO) 481 (WLC)			*M* age = 35; 100% male; 17% Aborig.			Prison	% Reincarcerated (official)		36 months (max)	Canada	CIHR/PHAC Research Chair in Applied Public Health No DOIs
Marsden et al. (2017) [2010–2016]	8645 (MMT or BUP) 6496 (NO)^n^			*M* age = 35; 76% male	*Mdn* 40 mg MMT/8 mg BUP		Prison	Group mortality rate (i.e., ratio of fatal overdoses to 100 person years) (official)		12 months (max)	England	National Health Service (England) DOIs^o^
Westerberg et al. (2016) [2011–2012]	117 (MMT) 237 (DTX)			67% male; 24% White; 3% Black; 70% Lat.; 3% Other	Clinically indicated. Delivered by a local MMT provider.		Jail (continued from community)	% Reincarcerated *M* # Days to reincarceration (official)		12 months	New Mexico, USA	County of Bernalillo University of New Mexico No DOIs

^a^ Sample sizes reflect groups at the outset of the study (absent attrition). Sample shorthand: H‐MMT and l‐MMT = High‐dose methadone maintenance treatment (MMT) and low‐dose MMT; DTX = Detoxification/forced withdrawal; WLC = Waitlist control; BUP = Buprenorphine; ONX = Oral naltrexone; COUN = non‐specified counseling; INX = Injectable naltrexone; NO = dissemination of educational materials and/or treatment referrals from a counselor, but no sustained intervention); INX‐PN = Injectable naltrexone with patient navigation; MMT‐C = continued MMT; MMT‐T = Terminated MMT; LAAM = levo‐alpha‐acetylmethadol; ELIG = Randomized into the treatment group, but did not begin due to other reasons; NTX = naltrexone implant; SUB = Suboxone (combination buprenorphine and naloxone).

^b^ Totals may not sum to 100% due to rounding for display purposes.

^c^ Represents “target dosage” unless otherwise indicated (e.g., *M*/*Mdn*). Missing information on treatment length and timing indicates these were not clearly specified in the respective article.

^d^ Includes only declarations of interest that are explicitly named by study authors.

^e^ “The initial naltrexone dose was 25 mg. During the first week, subjects returned for two more visits, and on the second visit the dose was increased to 50 mg and on the third visit the dose was 100 mg. Beginning in the second week, subjects receive 150 mg of naltrexone twice a week (300 mg per week) for a total of 26 weeks or 6 months” (Coviello et al., 2010, p. 424).

^f^ Commonwealth Department of Health and Family Services, Glaxo‐Wellcome, the NSW Department of Health and the National Drug and Alcohol Research Centre, UNSW

^g^ “Daily dosing was scheduled to be administered by study nurses for the first 49 days (1 mg, days 1–7; 2 mg, days 8–14; 3 mg, days 15–21; 4 mg, days 22–28; 6 mg, days 29–35, 8 mg; days 36–49). Days 50 through 63 dosing was scheduled every other day at 16 mg (or twice the daily dose). From day 64 onward until release from incarceration, participants were scheduled to receive 16 mg (or twice the daily dose) on Mondays and Wednesdays and 20 mg (or 2.5 times the daily dose) on Fridays. Allowance was made to increase the Friday dose to 3 times the daily dose if needed” (Gordon et al., 2018, p.35).

^h^ “This study was supported by an unrestricted, unsolicited investigator‐initiated request from Reckitt Benckiser Pharmaceuticals, Inc. (provided study drug only) …Drs. Gordon and Fitzgerald received investigator‐initiated funding and study drug from Alkermes. Dr. Blue reports no conflicts of interest. Dr. Schwartz did a one‐time consultation for Reckitt‐Benckiser on behalf of his employer (the Friends Research Institute). Dr. O'Grady has in the past received funding for his time from Reckitt‐Benckiser. Dr.Vocci reports personal fees and other from Braeburn Pharmaceuticals; personal fees and other from Pinney Associates; personal fees and other from Indivior, personal fees and other from Demerx, personal fees from Alkermes, personal fees and other from Insys Pharmaceuticals, and stock ownership from Intratab Labs, Inc.” (Gordon et al., 2018, p. 238)

^i^ “Dr. Schwartz serves as a senior fellow at the Open Society Institute‐Baltimore, which funded a previous study conducted by Dr. Kinlock et al., 2008, p. 238).

^j^ “Dr. Lee reports receiving grant support and study medication from Alkermes and study medication from Indivior (formerly Reckitt Benckiser). Dr. Friedmann reports receiving fees for serving on an advisory board and travel support from Indivior and an honorarium for leading a roundtable discussion from Orexo. Dr. Kinlock reports receiving grant support and study medication from Alkermes. Dr. Nunes reports serving on an advisory board for Alkermes, receiving study medication from Reckitt Benckiser and Duramed Pharmaceuticals, being lead investigator for a NIDA‐funded study of a computer‐delivered behavioral intervention supplied by HealthSim, and being site principal investigator for a study funded by, and receiving travel support from, Brainsway. Dr. Rotrosen reports receiving study medication from Alkermes and Indivior. Dr. Gordon reports receiving grant support and study medication from Alkermes. Dr. Fishman reports receiving travel support from Alkermes. Dr. O'Brien reports receiving consulting fees from Alkermes” (Lee et al., 2016, p. 1241).

^k^ KEEP = Key Extended Entry Program, delivered by Prison Health Services with oversight by the New York City Department of Health and Mental Hygiene (DOHMH) within the Rikers Island jail complex in New York City.

^l^ CODAC Behavioral Healthcare provides community and jail/prison‐based opioid use disorder treatment in Rhode Island.

^m^ One comparison group from original study was excluded in this review because it was not exclusively opioid users, per correspondence with the author.

^n^ The OST unexposed group (control) did not receive OST, or had been withdrawn, or had a low dose (threshold was ≤20 mg MMT and ≤2 mg BUP).

^o^ “J.M. is supported by research grants from the Department of Health, Institute for Health Research (NIHR), Medical Research Council (Drugs Data Warehouse project with MH, Tim Millar, Graham Dun, Sheila Bird and Matthias Pierce) and the NIHR Biomedical Research Centre for Mental Health at South London and Maudsley NHS Mental Health Foundation Trust (SLaM MHFT). He has part‐time employment as Senior Academic Adviser for the Alcohol, Drugs and Tobacco Division, Health and Wellbeing Directorate, Public Health England. He declares grant funding at IoPPN and SLaM MHFT for a study of psychological interventions in OST (2010–2016; Indivior PLC via Action on Addiction), support from NIHR (HTA) for a trial of extended‐release naltrexone, and honoraria from Merck Serono (2013, 2015; clinical oncology medicine) and Indivior (via PCM Scientific) as speaker (2013), cochair (2015–16) and chair (2017) for the Improving Outcomes in Treatment of Opioid Dependence conference. M.H. acknowledges support from NIHR Health Protection Research Unit in Evaluation of Interventions, the NIHR School of Public Health Research, and the Medical Research Council (Drugs Data Warehouse project with J. M., Tim Millar, Graham Dun, Sheila Bird and Matthias Pierce). He has received unrestricted research grants and travel support from Gilead, Jansen and Merck Serono. H.J. acknowledges support from the Medical Research Council” (Marsden et al., 2017, p. 1416).

About two‐thirds of included studies (*n* = 13) had only one comparator condition, or if they had more than one, only one comparator condition was eligible for inclusion in this review. The remaining seven studies had two comparator conditions or only two comparator conditions that were eligible for inclusion. Two studies explicitly compared different types of opioid‐specific MAT, and four studies had a comparator condition that entailed either a different dosage or adherence level for the same drug or a MAT + condition (e.g., MAT with patient navigation). When the comparator conditions did not include any MAT, they were sub‐classified into two groups: (1) “no treatment” (*n* = 14; e.g., nothing or referral to treatment, wait list control, or detoxification); or (2) “treatment as usual” (e.g., non‐specified psychosocial treatment [*n* = 3] or probation + [*n* = 1]).

Across the 20 studies, 30,119 participants are represented. In total, 13,609 individuals (roughly 45%) received MAT, which includes those in the primary treatment condition (*n* = 12,031) and those receiving MAT in a comparator condition (*n* = 1,578). There were 16,510 individuals (roughly 55%) in a no‐treatment (*n* = 16,182) or psychosocial treatment (*n* = 360) comparator condition. A small proportion of studies employed male‐only samples (*n* = 5). Although the remaining studies had mixed‐gender samples, the majority of participants in each of the original studies were male. Racial and ethnic groups were defined and reported differently across studies, however a diversity of races and ethnicities are represented. Participants were, on average, in their 30s and 40s.

Most studies were conducted in the United States (*n* = 15), predominantly in the northeastern part of the country (MD [*n* = 5], RI [*n* = 3], NY [*n* = 3], PA [*n* = 4], CT [*n* = 1]), with two from New Mexico.^2^ The remaining studies (*n* = 5) yielded from Canada (*n* = 2), Norway (*n* = 1), England (*n* = 1), and Australia (*n* = 1). All studies examined the impact of MAT that was first administered while participants were incarcerated in a jail or prison setting. Outcomes were typically assessed at 12 months (*n* = 7) and 6 months (*n* = 5). One study reported three‐month outcomes, one reported nine‐month outcomes, three studies reported outcomes of two years or longer, and three reported variable lengths of follow up for different outcomes. Four studies representing 2,092 participants reported both criminal justice and overdose outcomes, fifteen studies inclusive of 12,886 participants reported only criminal justice outcomes, and one study of 15,141 participants reported only overdose outcomes. Funding for the included studies came from three general sources: (1) county, state, or federal government agencies (e.g., National Institute of Health, National Health Service, Department of Corrections, Centers for Disease Control and Prevention) (*n* = 17); (2) private research institutes or foundations (e.g., Open Society Institute, Arnold Foundation) (*n* = 3); and (3) pharmaceutical companies (e.g., Alkermes, Glaxo‐Wellcome, DuPont Pharmaceuticals) (*n* = 5).^3^

#### 4.1.3 Excluded studies

There are 17 total studies that, while excluded from the current review, are worth brief mention. As shown in Supporting Information Appendix 6, the nine quasi‐experimental studies listed in Group 1 meet most inclusion criteria for this review except that appropriate levels of statistical control were not used to account for baseline differences between the treatment and comparator conditions. These studies are included in the summary of excluded studies because they could—and should—be considered in future reviews that employ slightly more relaxed inclusion criteria. Group 3 includes seven studies that otherwise meet criteria for inclusion in this review but either still in ongoing data collection, or were not available or ready for inclusion in this review, per correspondence with the lead study authors. These studies should be included in any updates of the current review. Finally, the one study listed in Group 2 was removed after the assessment of Risk of Bias phase and before meta‐analysis and synthesis because it was determined to have “critical” risk of bias in the bias in deviation from interventions domain and moderate to serious risk of bias in five of six remaining domains. Per the ROBINS‐I guidelines, a rating of critical risk in any domain suggests that it should not be included in the synthesis.

### 4.2 Summary of the quality of included studies

#### 4.2.1 Experimental studies

Across the 14 experimental studies, none were rated as low risk of bias. Ten had some risk of bias, and four had high risk of bias. Overall study ratings and justifications are listed in Supporting Information Appendix 4.

##### 4.2.1.1 Bias arising from the randomization process

In total, 10 studies were rated low risk of bias and four as some concern in this domain (Cornish et al., 1997; Coviello et al., 2010; Kinlock et al., 2005; McKenzie et al., 2012). None were rated high risk of bias in this domain.

##### 4.2.1.2 Bias due to deviations from intended interventions

Three studies were rated high risk of bias in this domain (Kinlock et al., 2005; Lobmaier, Kunøe, & Waal, 2010; Schwartz et al., 2020), and the remaining 11 were rated as some concern. None were rated low risk of bias in this domain.

##### 4.2.1.3 Bias due to missing outcome data

One study was rated as high risk of bias in this domain (Coviello et al., 2010), and the remaining were divided into some concern (Brinkley‐Rubinstein et al., 2018; Farabee et al., 2020; Kinlock et al., 2005; Lobmaier, Kunøe, & Waal, 2010; Magura et al., 2009; McKenzie et al., 2012; Schwartz et al., 2020) and low risk of bias (Cornish et al., 1997; Dolan et al., 2005; Gordon et al., 2018; Hyatt et al., 2021; Kinlock et al., ; Lee et al., 2016).

##### 4.2.1.4 Bias in the measurement of the outcome

Almost all studies (*n* = 11) were rated as low risk of bias in this domain, and three were rated as some concern (Lobmaier et al., 2010; Magura et al., 2009; Schwartz et al., 2020). None were rated as high risk of bias in this domain.

##### 4.2.1.5 Bias in the selection of reported results

All studies were rated as low risk of bias in this domain.

#### 4.2.2 Quasi‐Experimental studies

The six quasi‐experimental studies included in this review can be considered among the most rigorous available to date that examine MAT for overdose and criminal justice outcomes among criminal justice samples. Even with the strict inclusion criteria, however, two had a moderate risk of bias, and two had a serious risk of bias. Two others were quite rigorous but were missing information needed to score at least one of the domains. All studies are included in the analyses despite their risk of bias. Supporting Information Appendix 5 details all risk of bias ratings and corresponding justifications.

##### 4.2.2.1 Bias due to confounding

One study was rated as serious risk (Westerberg et al., 2016), one as moderate risk (Bellin et al., 1999), and four as low risk (Farrell‐MacDonald et al., 2014; Haas, 2020; Marsden et al., 2017; McSwain et al., 2014) in this domain.

##### 4.2.2.2 Bias in selection of participants into the study

Three studies were rated as moderate risk (Bellin et al., 1999; Haas, 2020; Marsden et al., 2017) and three were rated as low risk of bias in this domain (Farrell‐MacDonald et al., 2014; McSwain et al., 2014; Westerberg et al., 2016).

##### 4.2.2.3 Bias in classification of the interventions

One study (Zaller et al., 2013) was rated as serious risk, one study as moderate risk (Marsden et al., 2017), and the remaining studies (*n* = 5) were rated as low risk of bias in this domain.

##### 4.2.2.4 Bias due to deviations from intended interventions

One study had low risk (Hass, 2020), one had moderate risk (Marsden et al., 2017), one had serious risk (Westerberg et al., 2016), and three studies (Bellin et al., 1999; Farrell‐MacDonald et al., 2014; McSwain et al., 2014) did not have enough information to be able to rate this domain.

##### 4.2.2.5 Bias due to missing data

One study had moderate risk (Marsden et al., 2017), and the remaining (*n* = 5) studies had low risk of bias in this domain.

##### 4.2.2.6 Bias in measurement of the outcomes

All studies had low risk of bias in this domain.

##### 4.2.2.7 Bias in the selection of reported results

One study had moderate risk of bias in this domain (Bellin et al., 1999). All others (*n* = 5) had low risk of bias in this domain.

### 4.3 Summary of findings

#### 4.3.1 Criminal justice outcomes (raw effects)

The raw effects of MAT on criminal justice outcomes across all included studies are displayed in Table 2.

**Table 2:** Summary of findings for raw effects of MAT on criminal justice outcomes

Study	Follow‐up	Treatment Control Comparator 2	Incarceration	Arrest	Conviction	Offending	Risk of bias
*Experimental studies*							
Brinkley‐Rubinstein et al. (2018)	12 months	Methadone	57%	49%			Some
		Detox	58%	57%			
Cornish et al. (1997)	6 months	Oral Naltrexone	**26%** a				Some
		Probation/Education	**56%**				
Coviello et al. (2010)^b^	6 months	Oral Naltrexone	*M* Months = 0.5 10% Drug offense 7% Parole Violations		*M* = 0.1	*M* Charges = 0.2	High
		Counseling	*M* months = 0.8 3% drug offenses 27% parole violations		*M* = 0.1	*M C*harges = 0.5	
Dolan et al. (2005)	48 months	Methadone	75%				Some
		Wait List Control	72%				
Farabee et al. (2020)	12 months	Injectable Naltrexone		43%			Some
		Wait List Control		37.5%			
		Injectable Naltrexone + Patient Navigation		30%			
Gordon et al. (2018)	12 months	Buprenorphine		50% Any *M* days to 206 (SD = 105) *M* = 0.9 (SD = 1.1)			Some
		Wail List Control		41.4% *M* days to 171 (SD = 113) *M* = 0.7 (SD = 0.99)			
Hyatt et al. (2021)	6 months	Injectable Naltrexone	23.4%	19.2%			Some
		Wait List Control	17.0%	8.5%			
Kinlock et al. (2005)	9 months	LAAM	29%	33%		*M* days = 40 (SD = 90)	High
		Referral	24%	55%		*M* days = 115 (SD = 126)	
		Non‐Completers	58%	58%		*M* days = 128 (SD = 137)	
Kinlock et al. ()	12 months	Methadone + Counseling		53%		*M* days = 82 (SD = 110)	Some
		Counseling		51%		*M* days = 107 (SD = 129)	
		Counseling + WLC		59%		*M* days = 65 (SD = 96)	
Lee et al. (2016)	6.25 months	Injectable Naltrexone	23% *M* days = 1651				Some
		Counseling	29% *M* days = 2628				
Lobmaier, Kunøe, and Gossop, Katevoll, et al. (2010)^c^	6 months	Methadone	22%			*M* days = 15 (*SD* = 12)	High
		Naltrexone Implant	24%			*M* days = 14 (SD = 13)	
Magura et al. (2009)^d^	3 months	Buprenorphine	40%	14% Drug *M* = 0.7 (SD = 1.0)			Some
		Methadone	50%	24% Drug *M* = 0.7 (SD = 0.8)			
McKenzie et al. (2012)	6 months	Methadone	8%	36%			High
		Referral + Financial Assistance	0%	19%			
		Referral w/No Financial Assistance	19.1%	29%			
Schwartz et al. (2020)^e^	12 months	Methadone				*M* days = 10 (SD = 2)	Some
		Detox				*M* days = 10 (SD = 2)	
		Methadone + Patient Navigation				*M* days = 7 (SD = 1.5)	
*Quasi‐experimental studies*							
Bellin et al. (1999)^f^	4‐23 Mos.	High Dose Methadone	***Mdn*** **Days to** = **253**				Serious
		Detox	***Mdn*** **Days to** = **337**				
		Low Dose Methadone	***Mdn*** **Days to** = **187**				
Farrell‐MacDonald et al. (2014)	<27 months	Methadone Continued	**20%** *M* Days to = 414				No Information
		No Treatment	**57%** *M* Days to = 415				
		Methadone Terminated	22% *M* Days to = 339				
Haas (2020)	Varied	Methadone	52%				Moderate
		No Treatment	49%				
MacSwain et al. (2014)	36 months	Methadone	40%				No Information
		No Treatment	48%				
		Wait List Control	52%				
Westerberg et al. (2016)	12 months	Methadone	53% ***M*** **Days to** = **276**				Serious
		Detox	72% ***M*** **Days to** = **236**				

*Note*: Bold values are statistical significance.

Abbreviation: MAT, medication‐assisted therapies.

^a^ Outcomes included in the table are those that most comparable to outcomes reported by other studies. The offenses for reincarceration that are reported were selected because they were the most common. Bold font indicates statistical significance.

^b^ This study is not included in the meta‐analysis as its outcomes differed too much from those observed across other studies.

^c^ This study was not included in the meta‐analyses as it compared two treatment conditions.

^d^ This study was not included in the meta‐analyses as it compared two treatment conditions.

^e^ This study is not included in the meta‐analysis as its outcome differed too much from those observed across other studies.

^f^ This study is not included in the meta‐analysis as significant differences were observed between treatment group and each comparator.

#### 4.3.2 Overdose outcomes (raw effects)

The raw effects of MAT on overdose outcomes across all included studies are displayed in Table 3.

**Table 3:** Summary of findings for raw effects of MAT on overdose outcomes

Study	Follow‐up	Treatment Control Comparator 2	Nonfatal	Fatal	Risk of Bias
Experimental studies					
Brinkley‐Rubinstein et al. (2018)	12 months	Methadone	6.3%		Some
		Detox	14.5%		
Lee et al. (2016)	<19.5 months	Injectable Naltrexone	0%	0%	Some
		Counseling	2.6%	1.3%	
McKenzie et al. (2012)	6 months	Methadone	12%	0%	High
		Referral + Financial Assistance	19%	3%	
		Referral w/No Financial Assistance	9.5%	11%	
Quasi‐experimental studies					
Haas (2020)	Varied	Methadone	**4.5%**	2.4%	Moderate
		No Treatment	**8%**	3%	
Marsden et al. (2017)	12 months	Buprenorphine or Methadone		0.3%	Moderate
		No Treatment		0.4%	

*Note*: Bold values are statistical significance.

Abbreviation: MAT, medication‐assisted therapies.

#### 4.3.3 Meta‐analysis of MAT effects on criminal justice outcomes

**Figure 2:**
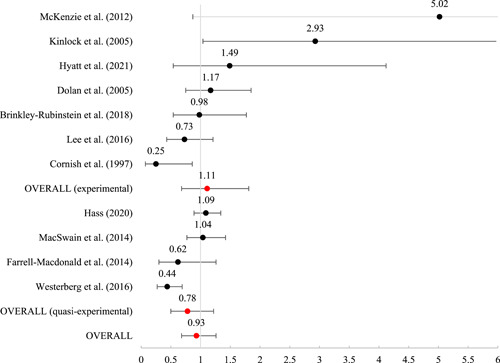
Reincarceration forest plot (corresponds with Table 4). Experimental studies are listed first, followed by quasi‐experimental studies. Effect sizes of individual studies and all confidence intervals are in black. Average effect sizes are in red. Vertical axis at 1 denotes a no‐effect value. Upper bound of confidence interval for McKenzie et al. (2012) is 28.90 and for Kinlock et al. (2005) is 8.26

**Figure 3:**
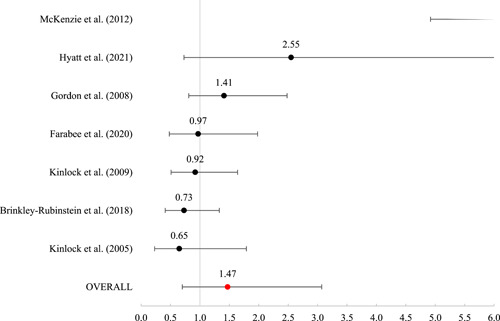
Rearrest forest plot (corresponds with Table 4). All studies are experimental. Effect sizes of individual studies and all confidence intervals are in black. Average effect sizes are in red. Vertical axis at 1 denotes a no‐effect value. Effect size for McKenzie et al. (2012) is 14.13 [4.92, 40.53]. Upper bound for confidence interval for Hyatt et al. (2021) is 8.94

Two separate criminal justice outcomes were meta‐analyzed: (1) reincarceration, which included seven experimental and four quasi‐experimental studies inclusive of 4,249 participants (*n* = 1,609 treatment and *n* = 2,640 control); and (2) rearrest, which included seven experimental studies inclusive of 1,151 participants (*n* = 475 treatment and *n* = 676 control). For reincarceration (*n* = 11), the overall mean effect was nonsignificant (OR = 0.93 [0.68, 1.26], SE = .16). This held for both experimental studies only (*n* = 7; OR = 1.11 [0.68, 1.81], SE = 0.25) and quasi‐experimental studies only (*n* = 4; OR = 0.78 [0.50, 1.22], SE = 0.23). For rearrest (*n* = 7, all experimental designs), the overall mean effect was also nonsignificant (OR = 1.47 [0.70, 3.07], SE = 0.38). Table 4 contains all standardized effects (i.e., mean odds ratios) as well as the results of the tests of subgroup homogeneity (experimental and quasi‐experimental) and the corresponding forest plots (Figures 2 and 3) are displayed below.

**Table 4:** Mean random‐effects odds ratio by criminal justice outcome and study design

Outcome	Study design^a^	Mean OR (*SE*)	Confidence interval		*K* b	τ^2^	*Q* c (*df*)
			Low	High			
Reincarceration	1	1.11 (0.25)	0.68	1.81	7	0.24	
	2	0.78 (0.23)	0.50	1.22	4	0.16	
	3	0.93 (0.16)	0.68	1.26	11	0.15	1.08 (1)
Rearrest	1	1.47 (0.38)	0.70	3.07	7	0.82	

*p < 0.05. *p* is based on *z*‐tests.

^a^ 1 = Experimental, 2 = Quasi‐Experimental, 3 = Overall.

^b^ Number of studies in group.

^c^ Test of between‐subgroup homogeneity is nonsignificant (*p* = 0.30).

#### 4.3.4 Meta‐analysis of MAT effects on overdose outcomes

**Figure 4:**
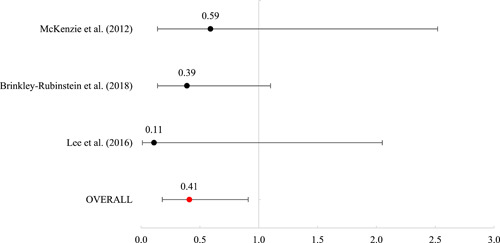
Nonfatal overdose forest plot (corresponds with Table 5). All studies are experimental. Effect sizes of individual studies and all confidence intervals are in black. Average effect sizes are in red. Vertical axis at 1 denotes a no‐effect value

**Figure 5:**
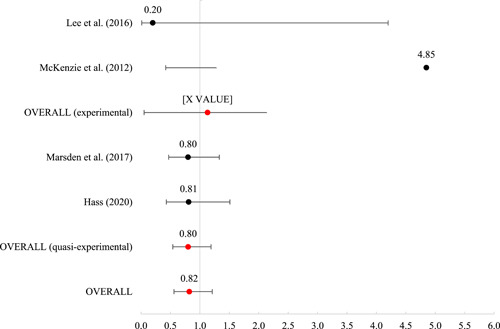
Fatal overdose forest plot (corresponds with Table 5). All studies are experimental. Effect sizes of individual studies and all confidence intervals are in black. Average effect sizes are in red. Vertical axis at 1 denotes a no‐effect value. Upper bound of confidence interval for overall (experimental) group is 25.41 and for McKenzie et al. (2012) is 55.53

For standardized average effects, fatal and nonfatal overdoses were meta‐analyzed separately. For fatal overdose, analyses included two experimental and two quasi‐experimental studies representing 17,273 participants (*n* = 9,483 treatment and *n* = 7,790 control). The mean effect across these four studies was nonsignificant (OR = 0.82 [0.56, 1.21], SE = 0.20), as were the individual effects for both experimental studies (*n* = 2; OR = 1.13 [0.05, 25.41], SE = 1.59) and quasi‐experimental studies (*n* = 2; OR = 0.80 [0.54, 1.19], SE = 0.20). The analysis of the nonfatal overdose outcome included three experimental studies inclusive of 2,245 participants (*n* = 1,087 treatment and *n* = 1,158 control). The average effect for this outcome was significant across the experimental studies (*n* = 3; OR = 0.41 [0.18, 0.91], SE = 0.41, *p* < 0.05). Results suggest that those receiving MAT had, on average, 59% lower odds of a nonfatal overdose than those receiving no treatment (or treatment as usual). Table 5 contains all standardized effects as well as the results of the tests of subgroup homogeneity (experimental and quasi‐experimental) and the corresponding forest plots (Figures 4 and 5) are displayed below.

**Table 5:** Mean random‐effects odds ratio by overdose outcome and study design

Outcome	Study design^a^	Mean OR (SE)	Confidence interval		*K* b	*τ* ^2^	*Q* c (df)
			Low	High			
Nonfatal overdose	1	0.41* (0.41)	0.18	0.91	3	0.00	
Fatal overdose	1	1.13 (1.59)	0.05	25.41	2	3.11	
	2	0.80 (0.20)	0.54	1.19	2	0.00	
	3	0.82 (0.20)	0.56	1.21	4	0.00	0.05 (1)

*p < 0.05. *p* is based on *z*‐tests.

^a^ 1 = Experimental, 2 = Quasi‐Experimental, 3 = Overall.

^b^ Number of studies in group.

^c^ Test of between‐subgroup homogeneity is nonsignificant (*p* = 0.83).

## 5 DISCUSSION

### 5.1 Summary of main results

Quantitative synthesis of the most methodologically rigorous studies available to date suggest a large effect of MAT on nonfatal overdose outcomes (59% reduction in odds) for justice‐involved individuals who misuse opioids. The impact of MAT on fatal overdose and criminal justice outcomes, however, can best be described as mixed and nonsignificant. For reincarceration, the overall findings were nonsignificant and essentially null, with no clear pattern favoring either condition. The subgroup analyses yielded average nonsignificant effects in opposite directions with more favorable results observed in less rigorous designs (as would be expected). Concerning rearrest, the nonsignificant findings favored the comparison group, though there was considerable variability across studies. For fatal overdose the overall findings were also nonsignificant but favored the treatment group, though the subgroup analyses once again produced average effects in different directions.

### 5.2 Overall completeness and applicability of evidence

Due to the variability with which the original studies reported details about their samples, this review was unable to examine the impact of most planned moderators. Additionally, this review did not examine cost effectiveness or the impact of MAT on opioid relapse. These are two important, policy relevant outcomes that would be useful to consider in future reviews. Several studies that otherwise met inclusion criteria for this review could not be included because either data collection was ongoing, or the data were not available for sharing at the time of analysis. Overall, our findings are applicable to institutional or community agencies that serve justice‐involved people who misuse opioids and have or are considering implementing MAT.

### 5.3 Quality of the evidence

There are several important methodological limitations in the original studies included in this review. Across all the included studies, none were given a low risk of bias rating across two independent coders. In both quasi‐experimental and experimental designs, deviations from the intended intervention/group assignment were consistently problematic. Underpowered studies, poor medication adherence among treatment participants (when these data were available), compensatory contamination of control participants, and missing or insufficient information accounting for experimental and control group contamination may have biased our results. The findings could also be a function of how study outcomes were measured. For instance, although most jurisdictions routinely track fatal overdose rates in specific communities, very few track these at the individual level in a systematic way that includes identifiable information. This makes it difficult for researchers to access and link these outcomes to individual study participants.

For criminal outcomes, three of the four indices examined in this review were typically accessed through official record, which can provide inaccurate estimates of the true incidence of criminality. Further, procedural factors (e.g., bail, plea bargaining) often play an important role in outcomes such as conviction or incarceration. In contrast, self‐reported criminal behavior may provide a more “pure” and potentially more accurate index of criminality; however, it was often assessed in the included studies using the Addiction Severity Index, which requires respondents to report outcomes only over the “past 30 days.” Thus, for both official records and self‐report in these studies, there is a likely underestimation of criminal behavior.

### 5.4 Potential biases in the review process

This review included an agency report, a student dissertation, and several peer reviewed publications. Additionally, the study authors were fairly liberal in the inclusion of citations during the screening stage so as to ensure that no eligible but unpublished or not‐yet‐published studies were excluded from consideration. The inclusion of several conference presentations and clinical trial protocols allowed for the identification of studies that were missed in the initial search. This approach also permitted the identification of several studies that would have otherwise been included in this review if the timing were later (and should be included in an update). Indeed, seven studies meeting inclusion criteria were still in active data collection and/or were not yet available for sharing with our team (per study authors).

### 5.5 Agreements or disagreements with other studies or reviews

To our knowledge there are no other systematic reviews that include only studies which assess opioid‐specific MAT effectiveness for criminal justice and overdose outcomes specific to justice‐involved populations. We believe this is an important distinction as this group likely differs from the general population in terms of their relative levels of “risk” and “need.” As the current review is an update and expansion of Egli et al. (2009), however, it is important to compare findings. The Egli et al. (2009) team found that certain medications *did* significantly reduce offending behaviors, which stands in contrast to the current study's findings. This may have resulted from key differences between the two reviews—namely, that Egli et al. (2009) included studies that were lower in methodological rigor and included types of MAT that were not assessed in the current review (e.g., heroin maintenance—which produced their largest effect) despite being eligible for inclusion. Current study findings are more in line with the systematic review by Moore et al. (2018), which considered the impacts of MAT on offending and substance use outcomes for incarcerated populations and found null effects specific to criminal recidivism.

## 6 AUTHORS' CONCLUSIONS

Our results suggest that MAT can yield meaningful reductions in nonfatal overdose among those involved in the criminal justice system. They do not support MAT's ability to reduce fatal overdose or criminal outcomes for people with current or prior justice system involvement. Given the design rigor of the included studies, this conclusion might be considered by some to be foregone. While it is possible that MAT is ineffective at reducing these outcomes, substantial methodological issues outside the main design render these findings more tentative. Under more ideal research conditions (e.g., where medication adherence was improved, attrition was reduced, fatal overdose data were more readily accessible, and sample sizes were larger), the study authors would be more confident in the estimates of MAT's impacts on overdose and criminal outcomes.

### 6.1 Implications for research

Corresponding to the shift toward medicalization (vs. criminalization) of addiction, there has been a substantial uptick in research on MAT in criminal justice settings and samples over the past several years. Studies employing rigorous methodologies are few and far between, relative to those employing single‐group (pre–post) or weaker quasi‐experimental designs. Arguably, it is quite difficult to conduct experimental research with criminal justice samples. Institutional context and regulations can impede successful implementation of study protocols. Additionally, instability across a number of domains (e.g., housing, transportation, employment/income, social support) post incarceration and/or during or post adjudication makes it difficult for people to maintain adherence to MAT. As such, researchers should work closely with agencies to improve treatment group medication adherence and to monitor and account for control group contamination. Collecting more detailed participant demographic information, as well as symptom onset and severity information and treatment and criminal histories would allow for more nuanced analyses examining “for whom” and “under what conditions” MAT would be most impactful for justice‐involved persons who misuse opioids. Additionally, outcomes should be assessed in multiple ways, if possible (e.g., self‐report and official record), as enhanced validity in outcome measurement can provide better effect estimates. Finally, participants' outcomes should be carefully tracked at multiple time points and over an extended period. This would help determine: (a) whether the impact of MAT on criminal and overdose outcomes is linear; (b) which is the optimal timing and length of MAT treatment for justice‐involved people with opioid misuse; and (c) whether MAT can have lasting impacts over time.

### 6.2 Implications for practice

The opioid epidemic is now in its third decade with no signs of slowing. Whole communities feel the toll of this crisis. As policymakers and practitioners work to identify solutions to reduce the harm of opioid addiction, particularly for public health and criminal justice outcomes, they must deploy multiple strategies at once and emphasize those that have the strongest impact and evidence. MAT is one tool in this effort. Its harm reduction utility in treatment‐seeking samples is well‐established. The findings from this review suggest that MAT's impact on nonfatal overdose also extends to individuals who are justice system involved, though the findings must be interpreted in light of considerable risk of bias in the evidence. One must be cautious not to oversell the promise of MAT as an antidote to criminality and overdose among people involved in the justice system. Indeed, these are highly complex social and health outcomes; both addiction and criminal behavior are influenced by a wide range of risk factors that also must be targeted in interventions. Timely access to appropriate and evidence‐based treatment must be coupled with an infrastructure of resources and social support.

## CONFLICT OF INTERESTS

One member of the author team, Dr. Jordan Hyatt, conducted a study that was included in this review. The team took the steps to ensure that he was not involved in the decision‐making concerning its inclusion or exclusion. There are no other potential conflicts of interest in this study.

## AUTHOR CONTRIBUTIONS

The contributions of the author team are as follows: C. Clare Strange developed the search strings, conducted and documented the search, managed the screening software, developed the coding protocol, screened and coded studies, developed all tables and figures, and participated in writing the full manuscript; Sarah M. Manchak developed the coding protocol and trained the coding team, screened studies during both the initial and quality assurance phases, coded studies, conducted risk of bias assessments, assisted with study tables, and assisted with writing the full manuscript; Jordan M. Hyatt coded studies and conducted the analyses; Damon M. Petrich screened and coded studies and conducted risk of bias assessments; Alisha Desai coded studies and participated in editing the full manuscript; and Cory Haberman conducted risk of bias assessments. All study authors (with the exception of DP who had not yet joined the project) contributed to conceptualizing and/or writing the study's protocol.

## DIFFERENCES BETWEEN PROTOCOL AND REVIEW

There were deviations from the study protocol at search, screening, coding, and analysis stages. These decisions are detailed below in the order that they were made.

To streamline the search process, we reduced the number of strings from seven to two: one for the group of criminal justice outcomes and one for overdose. This did not change the substantive nature of the search but combined the terms into strings representing either category of outcome.

Delimiters were also added to the search strings for certain databases (e.g., searching specific indices or source types) to draw more relevant results and/or temper the high volume retrieved. This was done under the guidance of the Campbell Collaboration and the delimiters used are noted in Supporting Information Appendix 1.

Several platforms/databases were removed or replaced due poor search functionality and/or the inability to refine sufficiently large results. These included the following platforms (and databases): Gale (Expanded Academic ASAP, Opposing Viewpoints Resource Center), FirstSearch (GPO Monthly Catalog, PapersFirst), Office of Justice Programs (National Criminal Justice Reference Service), and Nexis Uni. These sources overlapped in journal coverage with the databases that were used, which suggested they also would not add much unique content to the results. ClinicalTrials. gov was replaced with the Cochrane Register of Trials and MEDLINE to draw more relevant results by way of better search refinement tools. Summon was removed as the author team did not have institutional access to this database. Science. gov could be searched through crimesolutions. gov and therefore did not need to be searched separately. Last, Google Scholar was used to “forward search” included articles only, as there was no reliable way to search this database and no transparency in the search algorithms. All decisions to remove or replace databases specified in the protocol were done after much troubleshooting and in conjunction with the Campbell Collaboration.

The expanded list of criminal justice outcomes specified in the protocol (including specialized court docket failure, mandated treatment failure, and revocation of community supervision) was reduced to self‐reported and official indices of offending, arrest, conviction, and incarceration. This made our review a “pure” update in terms of the original categories of criminal justice outcomes reported in Egli et al. (2009). This decision was made in conjunction with the Campbell Collaboration in the interest of limiting the high volume of search results. This decision also meant that search strings featuring criminal justice outcomes could be given a publication filter of January 1, 2007 to October 31, 2020 as Egli et al. (2009) screened the relevant records before that date. This change was implemented post hoc by the Campbell Collaboration using the EndNote® reference management software, therefore the search strings specific to criminal justice outcomes in Supporting Information Appendix 1 still reflect a publication date filter of January 1, 1960 to October 31, 2020.

In the protocol we specified that the total number of studies retrieved from each string and database would be recorded once deduplication was complete. Deduplication was done by the Campbell Collaboration at a later stage (once all Research Information Systems [RIS] files were combined and uploaded into the EndNote® reference management software) and so the deduplicated number could not be traced back to the individual string and database. Instead, the number of results per string per database was recorded before deduplication.

The study team did not specify reference screening software in the protocol. Given the high volume of search results (*n* = 27,361 after deduplication), the team purchased access to DistillerSR® (at the recommendation of the Campbell Collaboration) and proceeded with screening using a team of graduate students in addition to three of the study authors. Using the DistillerSR® "Check for Screening Errors" tool also afforded us the opportunity to replace the protocol's initial strategies outlined to hand‐check for screening errors with a more reliable method.

In terms of coding, it was reported in the protocol that we would use the same coding scheme as Egli et al. (2009). We instead modified their coding scheme to capture differences in the outcomes examined and in the methodological and reporting standards. This updated coding scheme is included in Supporting Information Appendix 3.

Due to time constraints, we could not contact all authors of the included studies for unpublished data. However, multiple databases that we searched included gray literature and drew unpublished results (some of which were tied to the included studies, including conference presentations). This supports that the risk of publication bias remains low even without having contacting study authors. This is also why we did not display or report contour enhanced funnel plots.

In terms of the analyses, we did not examine the effects of moderators because they were not recorded in a consistent manner in the original studies. Furthermore, in terms of the timing of treatment as a potential moderator, nearly all interventions were initiated while individuals were incarcerated and then followed up in the community. As such, there was little to no variability to examine.

Last, continuous or quasi‐continuous measures of outcomes (e.g., average number of arrests) were rarely and inconsistently reported across studies. These results were not meta‐analyzed but instead reported in a narrative format.

## SOURCES OF SUPPORT

This study/project is funded by the National Institute for Health Research (NIHR) Incentive Award Scheme 2020 Reference 133293. The views expressed are those of the author(s) and not necessarily those of the NIHR or the Department of Health and Social Care.

## Supporting information

Supporting information.

## Footnotes

1 Total exceeds 14 because two studies compared different types of MAT.

2 Total exceeds 15 because one study spanned four states.

3 Total exceeds 20 as some studies received funding from multiple types of sources.
