# Supplementary material for: Opioid‐specific medication‐assisted therapy and its impact on criminal justice and overdose outcomes
Source: Campbell Syst Rev. 2022 Jan 7;18(1):e1215. doi: 10.1002/cl2.1215 (PMC8742132; doi:10.1002/cl2.1215)
Supplement: Supplementary file 1 — Supporting information. [file CL2-18-e1215-s001.docx]

# Appendices

## Appendix 1. Search Platforms, Databases, URLs, and Search Fields

| **Database**  **Platform**  **Date Searched** | **String** | **Expanders/**  **Delimiters** |
| --- | --- | --- |
| Criminal Justice Abstracts with Full Text  EBSCO  5/13/21 | ( TI ( "Medication assisted treat*" OR "Medication assisted therap*" OR drug* OR "therapeutic use*" OR opioid* OR "replacement therap*" OR "substitution therap*" OR pharmacotherap* OR "pharmacological treatment*" OR addict* OR agonist* OR "partial agonist*" OR methadone OR methadose OR dolophine OR buprenorphine OR suboxone OR sublocade OR naltrexone OR depade OR vivitrol OR revia OR "levomethadyl acetate" OR LAAM OR orlaam OR morphine OR analgesic* OR heroin OR narcotic OR heroin OR intravenous OR "dose-response relationship*" OR "drug prescription*" ) OR AB ("Medication assisted treat*" OR "Medication assisted therap*" OR drug* OR "therapeutic use*" OR opioid* OR "replacement therap*" OR "substitution therap*" OR pharmacotherap* OR "pharmacological treatment*" OR addict* OR agonist* OR "partial agonist*" OR methadone OR methadose OR dolophine OR buprenorphine OR suboxone OR sublocade OR naltrexone OR depade OR vivitrol OR revia OR "levomethadyl acetate" OR LAAM OR orlaam OR morphine OR analgesic* OR heroin OR narcotic OR heroin OR intravenous OR "dose-response relationship*" OR "drug prescription*" ) OR KW ("Medication assisted treat*" OR "Medication assisted therap*" OR drug* OR "therapeutic use*" OR opioid* OR "replacement therap*" OR "substitution therap*" OR pharmacotherap* OR "pharmacological treatment*" OR addict* OR agonist* OR "partial agonist*" OR methadone OR methadose OR dolophine OR buprenorphine OR suboxone OR sublocade OR naltrexone OR depade OR vivitrol OR revia OR "levomethadyl acetate" OR LAAM OR orlaam OR morphine OR analgesic* OR heroin OR narcotic OR heroin OR intravenous OR "dose-response relationship*" OR "drug prescription*") OR SU ("Medication assisted treat*" OR "Medication assisted therap*" OR drug* OR "therapeutic use*" OR opioid* OR "replacement therap*" OR "substitution therap*" OR pharmacotherap* OR "pharmacological treatment*" OR addict* OR agonist* OR "partial agonist*" OR methadone OR methadose OR dolophine OR buprenorphine OR suboxone OR sublocade OR naltrexone OR depade OR vivitrol OR revia OR "levomethadyl acetate" OR LAAM OR orlaam OR morphine OR analgesic* OR heroin OR narcotic OR heroin OR intravenous OR "dose-response relationship*" OR "drug prescription*" ) ) AND ( TI ( crim* OR incarcerat* OR convict* OR offend* OR offence* OR offense* OR reincarceration OR reconvict* OR reoffen* OR recidiv* OR rearrest* OR arrest* OR probation* OR parole* OR "community supervis*" OR "technical violat*" OR "drug court*" OR "special* court*" OR "treatment court*" ) OR AB ( crim* OR incarcerat* OR convict* OR offend* OR offence* OR offense* OR reincarceration OR reconvict* OR reoffen* OR recidiv* OR rearrest* OR arrest* OR probation* OR parole* OR "community supervis*" OR "technical violat*" OR "drug court*" OR "special* court*" OR "treatment court*" ) OR KW ( crim* OR incarcerat* OR convict* OR offend* OR offence* OR offense* OR reincarceration OR reconvict* OR reoffen* OR recidiv* OR rearrest* OR arrest* OR probation* OR parole* OR "community supervis*" OR "technical violat*" OR "drug court*" OR "special* court*" OR "treatment court*" ) OR SU (crim* OR incarcerat* OR convict* OR offend* OR offence* OR offense* OR reincarceration OR reconvict* OR reoffen* OR recidiv* OR rearrest* OR arrest* OR probation* OR parole* OR "community supervis*" OR "technical violat*" OR "drug court*" OR "special* court*" OR "treatment court*" ) ) AND ( TI ( "comparison condition*" OR "comparison group*" OR "control group*" OR "control condition" OR crossover OR effecti* OR efficac* OR evaluat* OR experiment* OR interven* OR match* OR metaanaly* OR pilot* OR placebo* OR program* OR “propensity score*” OR random* OR RCT OR review* OR service* OR therap* OR train* OR treat* OR trial* ) OR AB ("comparison condition*" OR "comparison group*" OR "control group*" OR "control condition" OR crossover OR effecti* OR efficac* OR evaluat* OR experiment* OR interven* OR match* OR metaanaly* OR pilot* OR placebo* OR program* OR “propensity score*” OR random* OR RCT OR review* OR service* OR therap* OR train* OR treat* OR trial* ) OR KW ( "comparison condition*" OR "comparison group*" OR "control group*" OR "control condition" OR crossover OR effecti* OR efficac* OR evaluat* OR experiment* OR interven* OR match* OR metaanaly* OR pilot* OR placebo* OR program* OR “propensity score*” OR random* OR RCT OR review* OR service* OR therap* OR train* OR treat* OR trial* ) OR SU ( "comparison condition*" OR "comparison group*" OR "control group*" OR "control condition" OR crossover OR effecti* OR efficac* OR evaluat* OR experiment* OR interven* OR match* OR metaanaly* OR pilot* OR placebo* OR program* OR “propensity score*” OR random* OR RCT OR review* OR service* OR therap* OR train* OR treat* OR trial*) ) | Apply equivalent subjects, Publication Date: 19600101-20201031, Search modes - Boolean/Phrase, Language: English |
| Criminal Justice Abstracts with Full Text  EBSCO  6/19/21 | ( TI ( "Medication assisted treat*" OR "Medication assisted therap*" OR drug* OR "therapeutic use*" OR opioid* OR "replacement therap*" OR "substitution therap*" OR pharmacotherap* OR "pharmacological treatment*" OR addict* OR agonist* OR "partial agonist*" OR methadone OR methadose OR dolophine OR buprenorphine OR suboxone OR sublocade OR naltrexone OR depade OR vivitrol OR revia OR "levomethadyl acetate" OR LAAM OR orlaam OR morphine OR analgesic* OR heroin OR narcotic OR heroin OR intravenous OR "dose-response relationship*" OR "drug prescription*" ) OR AB ( "Medication assisted treat*" OR "Medication assisted therap*" OR drug* OR "therapeutic use*" OR opioid* OR "replacement therap*" OR "substitution therap*" OR pharmacotherap* OR "pharmacological treatment*" OR addict* OR agonist* OR "partial agonist*" OR methadone OR methadose OR dolophine OR buprenorphine OR suboxone OR sublocade OR naltrexone OR depade OR vivitrol OR revia OR "levomethadyl acetate" OR LAAM OR orlaam OR morphine OR analgesic* OR heroin OR narcotic OR heroin OR intravenous OR "dose-response relationship*" OR "drug prescription*" ) OR KW ( "Medication assisted treat*" OR "Medication assisted therap*" OR drug* OR "therapeutic use*" OR opioid* OR "replacement therap*" OR "substitution therap*" OR pharmacotherap* OR "pharmacological treatment*" OR addict* OR agonist* OR "partial agonist*" OR methadone OR methadose OR dolophine OR buprenorphine OR suboxone OR sublocade OR naltrexone OR depade OR vivitrol OR revia OR "levomethadyl acetate" OR LAAM OR orlaam OR morphine OR analgesic* OR heroin OR narcotic OR heroin OR intravenous OR "dose-response relationship*" OR "drug prescription*" ) OR SU ( "Medication assisted treat*" OR "Medication assisted therap*" OR drug* OR "therapeutic use*" OR opioid* OR "replacement therap*" OR "substitution therap*" OR pharmacotherap* OR "pharmacological treatment*" OR addict* OR agonist* OR "partial agonist*" OR methadone OR methadose OR dolophine OR buprenorphine OR suboxone OR sublocade OR naltrexone OR depade OR vivitrol OR revia OR "levomethadyl acetate" OR LAAM OR orlaam OR morphine OR analgesic* OR heroin OR narcotic OR heroin OR intravenous OR "dose-response relationship*" OR "drug prescription*" ) ) AND ( TI ( crim* OR incarcerat* OR convict* OR offend* OR offence* OR offense* OR reincarceration OR reconvict* OR reoffen* OR recidiv* OR rearrest* OR arrest* OR probation* OR parole* OR "community supervis*" OR "technical violat*" OR "drug court*" OR "special* court*" OR "treatment court*" ) OR AB ( crim* OR incarcerat* OR convict* OR offend* OR offence* OR offense* OR reincarceration OR reconvict* OR reoffen* OR recidiv* OR rearrest* OR arrest* OR probation* OR parole* OR "community supervis*" OR "technical violat*" OR "drug court*" OR "special* court*" OR "treatment court*" ) OR KW ( crim* OR incarcerat* OR convict* OR offend* OR offence* OR offense* OR reincarceration OR reconvict* OR reoffen* OR recidiv* OR rearrest* OR arrest* OR probation* OR parole* OR "community supervis*" OR "technical violat*" OR "drug court*" OR "special* court*" OR "treatment court*" ) OR SU ( crim* OR incarcerat* OR convict* OR offend* OR offence* OR offense* OR reincarceration OR reconvict* OR reoffen* OR recidiv* OR rearrest* OR arrest* OR probation* OR parole* OR "community supervis*" OR "technical violat*" OR "drug court*" OR "special* court*" OR "treatment court*" ) ) AND ( TI (overdos*) OR AB (overdos*) OR KW (overdos*) OR SU (overdos*) ) | Apply equivalent subjects, Publication Date: 19600101-20201031, Search modes - Boolean/Phrase, Language: English |
| SocINDEX with Full Text  EBSCO  5/13/21 | ( TI ( "Medication assisted treat*" OR "Medication assisted therap*" OR drug* OR "therapeutic use*" OR opioid* OR "replacement therap*" OR "substitution therap*" OR pharmacotherap* OR "pharmacological treatment*" OR addict* OR agonist* OR "partial agonist*" OR methadone OR methadose OR dolophine OR buprenorphine OR suboxone OR sublocade OR naltrexone OR depade OR vivitrol OR revia OR "levomethadyl acetate" OR LAAM OR orlaam OR morphine OR analgesic* OR heroin OR narcotic OR heroin OR intravenous OR "dose-response relationship*" OR "drug prescription*" ) OR AB ("Medication assisted treat*" OR "Medication assisted therap*" OR drug* OR "therapeutic use*" OR opioid* OR "replacement therap*" OR "substitution therap*" OR pharmacotherap* OR "pharmacological treatment*" OR addict* OR agonist* OR "partial agonist*" OR methadone OR methadose OR dolophine OR buprenorphine OR suboxone OR sublocade OR naltrexone OR depade OR vivitrol OR revia OR "levomethadyl acetate" OR LAAM OR orlaam OR morphine OR analgesic* OR heroin OR narcotic OR heroin OR intravenous OR "dose-response relationship*" OR "drug prescription*" ) OR KW ("Medication assisted treat*" OR "Medication assisted therap*" OR drug* OR "therapeutic use*" OR opioid* OR "replacement therap*" OR "substitution therap*" OR pharmacotherap* OR "pharmacological treatment*" OR addict* OR agonist* OR "partial agonist*" OR methadone OR methadose OR dolophine OR buprenorphine OR suboxone OR sublocade OR naltrexone OR depade OR vivitrol OR revia OR "levomethadyl acetate" OR LAAM OR orlaam OR morphine OR analgesic* OR heroin OR narcotic OR heroin OR intravenous OR "dose-response relationship*" OR "drug prescription*") OR SU ("Medication assisted treat*" OR "Medication assisted therap*" OR drug* OR "therapeutic use*" OR opioid* OR "replacement therap*" OR "substitution therap*" OR pharmacotherap* OR "pharmacological treatment*" OR addict* OR agonist* OR "partial agonist*" OR methadone OR methadose OR dolophine OR buprenorphine OR suboxone OR sublocade OR naltrexone OR depade OR vivitrol OR revia OR "levomethadyl acetate" OR LAAM OR orlaam OR morphine OR analgesic* OR heroin OR narcotic OR heroin OR intravenous OR "dose-response relationship*" OR "drug prescription*" ) ) AND ( TI ( crim* OR incarcerat* OR convict* OR offend* OR offence* OR offense* OR reincarceration OR reconvict* OR reoffen* OR recidiv* OR rearrest* OR arrest* OR probation* OR parole* OR "community supervis*" OR "technical violat*" OR "drug court*" OR "special* court*" OR "treatment court*" ) OR AB ( crim* OR incarcerat* OR convict* OR offend* OR offence* OR offense* OR reincarceration OR reconvict* OR reoffen* OR recidiv* OR rearrest* OR arrest* OR probation* OR parole* OR "community supervis*" OR "technical violat*" OR "drug court*" OR "special* court*" OR "treatment court*" ) OR KW ( crim* OR incarcerat* OR convict* OR offend* OR offence* OR offense* OR reincarceration OR reconvict* OR reoffen* OR recidiv* OR rearrest* OR arrest* OR probation* OR parole* OR "community supervis*" OR "technical violat*" OR "drug court*" OR "special* court*" OR "treatment court*" ) OR SU (crim* OR incarcerat* OR convict* OR offend* OR offence* OR offense* OR reincarceration OR reconvict* OR reoffen* OR recidiv* OR rearrest* OR arrest* OR probation* OR parole* OR "community supervis*" OR "technical violat*" OR "drug court*" OR "special* court*" OR "treatment court*" ) ) AND ( TI ( "comparison condition*" OR "comparison group*" OR "control group*" OR "control condition" OR crossover OR effecti* OR efficac* OR evaluat* OR experiment* OR interven* OR match* OR metaanaly* OR pilot* OR placebo* OR program* OR “propensity score*” OR random* OR RCT OR review* OR service* OR therap* OR train* OR treat* OR trial* ) OR AB ("comparison condition*" OR "comparison group*" OR "control group*" OR "control condition" OR crossover OR effecti* OR efficac* OR evaluat* OR experiment* OR interven* OR match* OR metaanaly* OR pilot* OR placebo* OR program* OR “propensity score*” OR random* OR RCT OR review* OR service* OR therap* OR train* OR treat* OR trial* ) OR KW ( "comparison condition*" OR "comparison group*" OR "control group*" OR "control condition" OR crossover OR effecti* OR efficac* OR evaluat* OR experiment* OR interven* OR match* OR metaanaly* OR pilot* OR placebo* OR program* OR “propensity score*” OR random* OR RCT OR review* OR service* OR therap* OR train* OR treat* OR trial* ) OR SU ( "comparison condition*" OR "comparison group*" OR "control group*" OR "control condition" OR crossover OR effecti* OR efficac* OR evaluat* OR experiment* OR interven* OR match* OR metaanaly* OR pilot* OR placebo* OR program* OR “propensity score*” OR random* OR RCT OR review* OR service* OR therap* OR train* OR treat* OR trial*) ) | Apply equivalent subjects, Publication Date: 19600101-20201031, Search modes - Boolean/Phrase, Language: English |
| SocINDEX with Full Text  EBSCO  5/9/21 | ( TI ( "Medication assisted treat*" OR "Medication assisted therap*" OR drug* OR "therapeutic use*" OR opioid* OR "replacement therap*" OR "substitution therap*" OR pharmacotherap* OR "pharmacological treatment*" OR addict* OR agonist* OR "partial agonist*" OR methadone OR methadose OR dolophine OR buprenorphine OR suboxone OR sublocade OR naltrexone OR depade OR vivitrol OR revia OR "levomethadyl acetate" OR LAAM OR orlaam OR morphine OR analgesic* OR heroin OR narcotic OR heroin OR intravenous OR "dose-response relationship*" OR "drug prescription*" ) OR AB ("Medication assisted treat*" OR "Medication assisted therap*" OR drug* OR "therapeutic use*" OR opioid* OR "replacement therap*" OR "substitution therap*" OR pharmacotherap* OR "pharmacological treatment*" OR addict* OR agonist* OR "partial agonist*" OR methadone OR methadose OR dolophine OR buprenorphine OR suboxone OR sublocade OR naltrexone OR depade OR vivitrol OR revia OR "levomethadyl acetate" OR LAAM OR orlaam OR morphine OR analgesic* OR heroin OR narcotic OR heroin OR intravenous OR "dose-response relationship*" OR "drug prescription*" ) OR KW ("Medication assisted treat*" OR "Medication assisted therap*" OR drug* OR "therapeutic use*" OR opioid* OR "replacement therap*" OR "substitution therap*" OR pharmacotherap* OR "pharmacological treatment*" OR addict* OR agonist* OR "partial agonist*" OR methadone OR methadose OR dolophine OR buprenorphine OR suboxone OR sublocade OR naltrexone OR depade OR vivitrol OR revia OR "levomethadyl acetate" OR LAAM OR orlaam OR morphine OR analgesic* OR heroin OR narcotic OR heroin OR intravenous OR "dose-response relationship*" OR "drug prescription*") OR SU ("Medication assisted treat*" OR "Medication assisted therap*" OR drug* OR "therapeutic use*" OR opioid* OR "replacement therap*" OR "substitution therap*" OR pharmacotherap* OR "pharmacological treatment*" OR addict* OR agonist* OR "partial agonist*" OR methadone OR methadose OR dolophine OR buprenorphine OR suboxone OR sublocade OR naltrexone OR depade OR vivitrol OR revia OR "levomethadyl acetate" OR LAAM OR orlaam OR morphine OR analgesic* OR heroin OR narcotic OR heroin OR intravenous OR "dose-response relationship*" OR "drug prescription*" ) ) AND ( TI ( crim* OR incarcerat* OR convict* OR offend* OR offence* OR offense* OR reincarceration OR reconvict* OR reoffen* OR recidiv* OR rearrest* OR arrest* OR probation* OR parole* OR "community supervis*" OR "technical violat*" OR "drug court*" OR "special* court*" OR "treatment court*" ) OR AB ( crim* OR incarcerat* OR convict* OR offend* OR offence* OR offense* OR reincarceration OR reconvict* OR reoffen* OR recidiv* OR rearrest* OR arrest* OR probation* OR parole* OR "community supervis*" OR "technical violat*" OR "drug court*" OR "special* court*" OR "treatment court*" ) OR KW ( crim* OR incarcerat* OR convict* OR offend* OR offence* OR offense* OR reincarceration OR reconvict* OR reoffen* OR recidiv* OR rearrest* OR arrest* OR probation* OR parole* OR "community supervis*" OR "technical violat*" OR "drug court*" OR "special* court*" OR "treatment court*" ) OR SU ( crim* OR incarcerat* OR convict* OR offend* OR offence* OR offense* OR reincarceration OR reconvict* OR reoffen* OR recidiv* OR rearrest* OR arrest* OR probation* OR parole* OR "community supervis*" OR "technical violat*" OR "drug court*" OR "special* court*" OR "treatment court*" ) ) AND ( TI (overdos*) OR AB (overdos*) OR KW (overdos*) OR SU (overdos*) ) | Apply equivalent subjects, Publication Date: 19600101-20201031, Search modes - Boolean/Phrase, Language: English |
| Legal Collection  EBSCO  5/13/21 | ( TI ( "Medication assisted treat*" OR "Medication assisted therap*" OR drug* OR "therapeutic use*" OR opioid* OR "replacement therap*" OR "substitution therap*" OR pharmacotherap* OR "pharmacological treatment*" OR addict* OR agonist* OR "partial agonist*" OR methadone OR methadose OR dolophine OR buprenorphine OR suboxone OR sublocade OR naltrexone OR depade OR vivitrol OR revia OR "levomethadyl acetate" OR LAAM OR orlaam OR morphine OR analgesic* OR heroin OR narcotic OR heroin OR intravenous OR "dose-response relationship*" OR "drug prescription*" ) OR AB ("Medication assisted treat*" OR "Medication assisted therap*" OR drug* OR "therapeutic use*" OR opioid* OR "replacement therap*" OR "substitution therap*" OR pharmacotherap* OR "pharmacological treatment*" OR addict* OR agonist* OR "partial agonist*" OR methadone OR methadose OR dolophine OR buprenorphine OR suboxone OR sublocade OR naltrexone OR depade OR vivitrol OR revia OR "levomethadyl acetate" OR LAAM OR orlaam OR morphine OR analgesic* OR heroin OR narcotic OR heroin OR intravenous OR "dose-response relationship*" OR "drug prescription*" ) OR KW ("Medication assisted treat*" OR "Medication assisted therap*" OR drug* OR "therapeutic use*" OR opioid* OR "replacement therap*" OR "substitution therap*" OR pharmacotherap* OR "pharmacological treatment*" OR addict* OR agonist* OR "partial agonist*" OR methadone OR methadose OR dolophine OR buprenorphine OR suboxone OR sublocade OR naltrexone OR depade OR vivitrol OR revia OR "levomethadyl acetate" OR LAAM OR orlaam OR morphine OR analgesic* OR heroin OR narcotic OR heroin OR intravenous OR "dose-response relationship*" OR "drug prescription*") OR SU ("Medication assisted treat*" OR "Medication assisted therap*" OR drug* OR "therapeutic use*" OR opioid* OR "replacement therap*" OR "substitution therap*" OR pharmacotherap* OR "pharmacological treatment*" OR addict* OR agonist* OR "partial agonist*" OR methadone OR methadose OR dolophine OR buprenorphine OR suboxone OR sublocade OR naltrexone OR depade OR vivitrol OR revia OR "levomethadyl acetate" OR LAAM OR orlaam OR morphine OR analgesic* OR heroin OR narcotic OR heroin OR intravenous OR "dose-response relationship*" OR "drug prescription*" ) ) AND ( TI ( crim* OR incarcerat* OR convict* OR offend* OR offence* OR offense* OR reincarceration OR reconvict* OR reoffen* OR recidiv* OR rearrest* OR arrest* OR probation* OR parole* OR "community supervis*" OR "technical violat*" OR "drug court*" OR "special* court*" OR "treatment court*" ) OR AB ( crim* OR incarcerat* OR convict* OR offend* OR offence* OR offense* OR reincarceration OR reconvict* OR reoffen* OR recidiv* OR rearrest* OR arrest* OR probation* OR parole* OR "community supervis*" OR "technical violat*" OR "drug court*" OR "special* court*" OR "treatment court*" ) OR KW ( crim* OR incarcerat* OR convict* OR offend* OR offence* OR offense* OR reincarceration OR reconvict* OR reoffen* OR recidiv* OR rearrest* OR arrest* OR probation* OR parole* OR "community supervis*" OR "technical violat*" OR "drug court*" OR "special* court*" OR "treatment court*" ) OR SU (crim* OR incarcerat* OR convict* OR offend* OR offence* OR offense* OR reincarceration OR reconvict* OR reoffen* OR recidiv* OR rearrest* OR arrest* OR probation* OR parole* OR "community supervis*" OR "technical violat*" OR "drug court*" OR "special* court*" OR "treatment court*" ) ) AND ( TI ( "comparison condition*" OR "comparison group*" OR "control group*" OR "control condition" OR crossover OR effecti* OR efficac* OR evaluat* OR experiment* OR interven* OR match* OR metaanaly* OR pilot* OR placebo* OR program* OR “propensity score*” OR random* OR RCT OR review* OR service* OR therap* OR train* OR treat* OR trial* ) OR AB ("comparison condition*" OR "comparison group*" OR "control group*" OR "control condition" OR crossover OR effecti* OR efficac* OR evaluat* OR experiment* OR interven* OR match* OR metaanaly* OR pilot* OR placebo* OR program* OR “propensity score*” OR random* OR RCT OR review* OR service* OR therap* OR train* OR treat* OR trial* ) OR KW ( "comparison condition*" OR "comparison group*" OR "control group*" OR "control condition" OR crossover OR effecti* OR efficac* OR evaluat* OR experiment* OR interven* OR match* OR metaanaly* OR pilot* OR placebo* OR program* OR “propensity score*” OR random* OR RCT OR review* OR service* OR therap* OR train* OR treat* OR trial* ) OR SU ( "comparison condition*" OR "comparison group*" OR "control group*" OR "control condition" OR crossover OR effecti* OR efficac* OR evaluat* OR experiment* OR interven* OR match* OR metaanaly* OR pilot* OR placebo* OR program* OR “propensity score*” OR random* OR RCT OR review* OR service* OR therap* OR train* OR treat* OR trial*) ) | Apply equivalent subjects, Publication Date: 19600101-20201031, Search modes - Boolean/Phrase |
| Legal Collection  EBSCO  5/7/21 | ( TI ( "Medication assisted treat*" OR "Medication assisted therap*" OR drug* OR "therapeutic use*" OR opioid* OR "replacement therap*" OR "substitution therap*" OR pharmacotherap* OR "pharmacological treatment*" OR addict* OR agonist* OR "partial agonist*" OR methadone OR methadose OR dolophine OR buprenorphine OR suboxone OR sublocade OR naltrexone OR depade OR vivitrol OR revia OR "levomethadyl acetate" OR LAAM OR orlaam OR morphine OR analgesic* OR heroin OR narcotic OR heroin OR intravenous OR "dose-response relationship*" OR "drug prescription*" ) OR AB ("Medication assisted treat*" OR "Medication assisted therap*" OR drug* OR "therapeutic use*" OR opioid* OR "replacement therap*" OR "substitution therap*" OR pharmacotherap* OR "pharmacological treatment*" OR addict* OR agonist* OR "partial agonist*" OR methadone OR methadose OR dolophine OR buprenorphine OR suboxone OR sublocade OR naltrexone OR depade OR vivitrol OR revia OR "levomethadyl acetate" OR LAAM OR orlaam OR morphine OR analgesic* OR heroin OR narcotic OR heroin OR intravenous OR "dose-response relationship*" OR "drug prescription*" ) OR KW ("Medication assisted treat*" OR "Medication assisted therap*" OR drug* OR "therapeutic use*" OR opioid* OR "replacement therap*" OR "substitution therap*" OR pharmacotherap* OR "pharmacological treatment*" OR addict* OR agonist* OR "partial agonist*" OR methadone OR methadose OR dolophine OR buprenorphine OR suboxone OR sublocade OR naltrexone OR depade OR vivitrol OR revia OR "levomethadyl acetate" OR LAAM OR orlaam OR morphine OR analgesic* OR heroin OR narcotic OR heroin OR intravenous OR "dose-response relationship*" OR "drug prescription*") OR SU ("Medication assisted treat*" OR "Medication assisted therap*" OR drug* OR "therapeutic use*" OR opioid* OR "replacement therap*" OR "substitution therap*" OR pharmacotherap* OR "pharmacological treatment*" OR addict* OR agonist* OR "partial agonist*" OR methadone OR methadose OR dolophine OR buprenorphine OR suboxone OR sublocade OR naltrexone OR depade OR vivitrol OR revia OR "levomethadyl acetate" OR LAAM OR orlaam OR morphine OR analgesic* OR heroin OR narcotic OR heroin OR intravenous OR "dose-response relationship*" OR "drug prescription*" ) ) AND ( TI ( crim* OR incarcerat* OR convict* OR offend* OR offence* OR offense* OR reincarceration OR reconvict* OR reoffen* OR recidiv* OR rearrest* OR arrest* OR probation* OR parole* OR "community supervis*" OR "technical violat*" OR "drug court*" OR "special* court*" OR "treatment court*" ) OR AB ( crim* OR incarcerat* OR convict* OR offend* OR offence* OR offense* OR reincarceration OR reconvict* OR reoffen* OR recidiv* OR rearrest* OR arrest* OR probation* OR parole* OR "community supervis*" OR "technical violat*" OR "drug court*" OR "special* court*" OR "treatment court*" ) OR KW ( crim* OR incarcerat* OR convict* OR offend* OR offence* OR offense* OR reincarceration OR reconvict* OR reoffen* OR recidiv* OR rearrest* OR arrest* OR probation* OR parole* OR "community supervis*" OR "technical violat*" OR "drug court*" OR "special* court*" OR "treatment court*" ) OR SU ( crim* OR incarcerat* OR convict* OR offend* OR offence* OR offense* OR reincarceration OR reconvict* OR reoffen* OR recidiv* OR rearrest* OR arrest* OR probation* OR parole* OR "community supervis*" OR "technical violat*" OR "drug court*" OR "special* court*" OR "treatment court*" ) ) AND ( TI (overdos*) OR AB (overdos*) OR KW (overdos*) OR SU (overdos*) ) | Apply equivalent subjects, Publication Date: 19600101-20201031, Search modes - Boolean/Phrase |
| MEDLINE  EBSCO  6/19/21 | ( TI ( "Medication assisted treat*" OR "Medication assisted therap*" OR drug* OR "therapeutic use*" OR opioid* OR "replacement therap*" OR "substitution therap*" OR pharmacotherap* OR "pharmacological treatment*" OR addict* OR agonist* OR "partial agonist*" OR methadone OR methadose OR dolophine OR buprenorphine OR suboxone OR sublocade OR naltrexone OR depade OR vivitrol OR revia OR "levomethadyl acetate" OR LAAM OR orlaam OR morphine OR analgesic* OR heroin OR narcotic OR heroin OR intravenous OR "dose-response relationship*" OR "drug prescription*" ) OR AB ("Medication assisted treat*" OR "Medication assisted therap*" OR drug* OR "therapeutic use*" OR opioid* OR "replacement therap*" OR "substitution therap*" OR pharmacotherap* OR "pharmacological treatment*" OR addict* OR agonist* OR "partial agonist*" OR methadone OR methadose OR dolophine OR buprenorphine OR suboxone OR sublocade OR naltrexone OR depade OR vivitrol OR revia OR "levomethadyl acetate" OR LAAM OR orlaam OR morphine OR analgesic* OR heroin OR narcotic OR heroin OR intravenous OR "dose-response relationship*" OR "drug prescription*" ) OR MJ ("Medication assisted treat*" OR "Medication assisted therap*" OR drug* OR "therapeutic use*" OR opioid* OR "replacement therap*" OR "substitution therap*" OR pharmacotherap* OR "pharmacological treatment*" OR addict* OR agonist* OR "partial agonist*" OR methadone OR methadose OR dolophine OR buprenorphine OR suboxone OR sublocade OR naltrexone OR depade OR vivitrol OR revia OR "levomethadyl acetate" OR LAAM OR orlaam OR morphine OR analgesic* OR heroin OR narcotic OR heroin OR intravenous OR "dose-response relationship*" OR "drug prescription*") ) AND ( TI ( crim* OR incarcerat* OR convict* OR offend* OR offence* OR offense* OR reincarceration OR reconvict* OR reoffen* OR recidiv* OR rearrest* OR arrest* OR probation* OR parole* OR "community supervis*" OR "technical violat*" OR "drug court*" OR "special* court*" OR "treatment court*" ) OR AB ( crim* OR incarcerat* OR convict* OR offend* OR offence* OR offense* OR reincarceration OR reconvict* OR reoffen* OR recidiv* OR rearrest* OR arrest* OR probation* OR parole* OR "community supervis*" OR "technical violat*" OR "drug court*" OR "special* court*" OR "treatment court*" ) OR MJ ( crim* OR incarcerat* OR convict* OR offend* OR offence* OR offense* OR reincarceration OR reconvict* OR reoffen* OR recidiv* OR rearrest* OR arrest* OR probation* OR parole* OR "community supervis*" OR "technical violat*" OR "drug court*" OR "special* court*" OR "treatment court*" ) ) AND ( TI ( "comparison condition*" OR "comparison group*" OR "control group*" OR "control condition" OR crossover OR effecti* OR efficac* OR evaluat* OR experiment* OR interven* OR match* OR metaanaly* OR pilot* OR placebo* OR program* OR “propensity score*” OR random* OR RCT OR review* OR service* OR therap* OR train* OR treat* OR trial* ) OR AB ("comparison condition*" OR "comparison group*" OR "control group*" OR "control condition" OR crossover OR effecti* OR efficac* OR evaluat* OR experiment* OR interven* OR match* OR metaanaly* OR pilot* OR placebo* OR program* OR “propensity score*” OR random* OR RCT OR review* OR service* OR therap* OR train* OR treat* OR trial* ) OR MJ ( "comparison condition*" OR "comparison group*" OR "control group*" OR "control condition" OR crossover OR effecti* OR efficac* OR evaluat* OR experiment* OR interven* OR match* OR metaanaly* OR pilot* OR placebo* OR program* OR “propensity score*” OR random* OR RCT OR review* OR service* OR therap* OR train* OR treat* OR trial* ) ) | Publication date: 19600101-20201031; English Language; Language: English, Apply equivalent subjects Narrow by SubjectMajor: - substance abuse, intravenous, opioid-related disorders, crime, prisoners, substance-related disorders, Search mode: Boolean/Phrase |
| MEDLINE  EBSCO  6/19/21 | ( TI ( "Medication assisted treat*" OR "Medication assisted therap*" OR drug* OR "therapeutic use*" OR opioid* OR "replacement therap*" OR "substitution therap*" OR pharmacotherap* OR "pharmacological treatment*" OR addict* OR agonist* OR "partial agonist*" OR methadone OR methadose OR dolophine OR buprenorphine OR suboxone OR sublocade OR naltrexone OR depade OR vivitrol OR revia OR "levomethadyl acetate" OR LAAM OR orlaam OR morphine OR analgesic* OR heroin OR narcotic OR heroin OR intravenous OR "dose-response relationship*" OR "drug prescription*" ) OR AB ( "Medication assisted treat*" OR "Medication assisted therap*" OR drug* OR "therapeutic use*" OR opioid* OR "replacement therap*" OR "substitution therap*" OR pharmacotherap* OR "pharmacological treatment*" OR addict* OR agonist* OR "partial agonist*" OR methadone OR methadose OR dolophine OR buprenorphine OR suboxone OR sublocade OR naltrexone OR depade OR vivitrol OR revia OR "levomethadyl acetate" OR LAAM OR orlaam OR morphine OR analgesic* OR heroin OR narcotic OR heroin OR intravenous OR "dose-response relationship*" OR "drug prescription*" ) OR MJ ( "Medication assisted treat*" OR "Medication assisted therap*" OR drug* OR "therapeutic use*" OR opioid* OR "replacement therap*" OR "substitution therap*" OR pharmacotherap* OR "pharmacological treatment*" OR addict* OR agonist* OR "partial agonist*" OR methadone OR methadose OR dolophine OR buprenorphine OR suboxone OR sublocade OR naltrexone OR depade OR vivitrol OR revia OR "levomethadyl acetate" OR LAAM OR orlaam OR morphine OR analgesic* OR heroin OR narcotic OR heroin OR intravenous OR "dose-response relationship*" OR "drug prescription*" ) ) AND ( TI ( crim* OR incarcerat* OR convict* OR offend* OR offence* OR offense* OR reincarceration OR reconvict* OR reoffen* OR recidiv* OR rearrest* OR arrest* OR probation* OR parole* OR "community supervis*" OR "technical violat*" OR "drug court*" OR "special* court*" OR "treatment court*" ) OR AB ( crim* OR incarcerat* OR convict* OR offend* OR offence* OR offense* OR reincarceration OR reconvict* OR reoffen* OR recidiv* OR rearrest* OR arrest* OR probation* OR parole* OR "community supervis*" OR "technical violat*" OR "drug court*" OR "special* court*" OR "treatment court*" ) OR MJ ( crim* OR incarcerat* OR convict* OR offend* OR offence* OR offense* OR reincarceration OR reconvict* OR reoffen* OR recidiv* OR rearrest* OR arrest* OR probation* OR parole* OR "community supervis*" OR "technical violat*" OR "drug court*" OR "special* court*" OR "treatment court*" ) ) AND ( TI ( overdos*) OR AB (overdos*) OR MJ (overdos*) ) | Publication date: 19600101-20201031; English Language; Language: English, Apply equivalent subjects, Search mode: Boolean/Phrase |
| Omnifile Full Text Mega  EBSCO  5/14/21 | ( TI ( "Medication assisted treat*" OR "Medication assisted therap*" OR drug* OR "therapeutic use*" OR opioid* OR "replacement therap*" OR "substitution therap*" OR pharmacotherap* OR "pharmacological treatment*" OR addict* OR agonist* OR "partial agonist*" OR methadone OR methadose OR dolophine OR buprenorphine OR suboxone OR sublocade OR naltrexone OR depade OR vivitrol OR revia OR "levomethadyl acetate" OR LAAM OR orlaam OR morphine OR analgesic* OR heroin OR narcotic OR heroin OR intravenous OR "dose-response relationship*" OR "drug prescription*" ) OR AB ("Medication assisted treat*" OR "Medication assisted therap*" OR drug* OR "therapeutic use*" OR opioid* OR "replacement therap*" OR "substitution therap*" OR pharmacotherap* OR "pharmacological treatment*" OR addict* OR agonist* OR "partial agonist*" OR methadone OR methadose OR dolophine OR buprenorphine OR suboxone OR sublocade OR naltrexone OR depade OR vivitrol OR revia OR "levomethadyl acetate" OR LAAM OR orlaam OR morphine OR analgesic* OR heroin OR narcotic OR heroin OR intravenous OR "dose-response relationship*" OR "drug prescription*" ) OR KW ("Medication assisted treat*" OR "Medication assisted therap*" OR drug* OR "therapeutic use*" OR opioid* OR "replacement therap*" OR "substitution therap*" OR pharmacotherap* OR "pharmacological treatment*" OR addict* OR agonist* OR "partial agonist*" OR methadone OR methadose OR dolophine OR buprenorphine OR suboxone OR sublocade OR naltrexone OR depade OR vivitrol OR revia OR "levomethadyl acetate" OR LAAM OR orlaam OR morphine OR analgesic* OR heroin OR narcotic OR heroin OR intravenous OR "dose-response relationship*" OR "drug prescription*") OR SU ("Medication assisted treat*" OR "Medication assisted therap*" OR drug* OR "therapeutic use*" OR opioid* OR "replacement therap*" OR "substitution therap*" OR pharmacotherap* OR "pharmacological treatment*" OR addict* OR agonist* OR "partial agonist*" OR methadone OR methadose OR dolophine OR buprenorphine OR suboxone OR sublocade OR naltrexone OR depade OR vivitrol OR revia OR "levomethadyl acetate" OR LAAM OR orlaam OR morphine OR analgesic* OR heroin OR narcotic OR heroin OR intravenous OR "dose-response relationship*" OR "drug prescription*" ) ) AND ( TI ( crim* OR incarcerat* OR convict* OR offend* OR offence* OR offense* OR reincarceration OR reconvict* OR reoffen* OR recidiv* OR rearrest* OR arrest* OR probation* OR parole* OR "community supervis*" OR "technical violat*" OR "drug court*" OR "special* court*" OR "treatment court*" ) OR AB ( crim* OR incarcerat* OR convict* OR offend* OR offence* OR offense* OR reincarceration OR reconvict* OR reoffen* OR recidiv* OR rearrest* OR arrest* OR probation* OR parole* OR "community supervis*" OR "technical violat*" OR "drug court*" OR "special* court*" OR "treatment court*" ) OR KW ( crim* OR incarcerat* OR convict* OR offend* OR offence* OR offense* OR reincarceration OR reconvict* OR reoffen* OR recidiv* OR rearrest* OR arrest* OR probation* OR parole* OR "community supervis*" OR "technical violat*" OR "drug court*" OR "special* court*" OR "treatment court*" ) OR SU ( crim* OR incarcerat* OR convict* OR offend* OR offence* OR offense* OR reincarceration OR reconvict* OR reoffen* OR recidiv* OR rearrest* OR arrest* OR probation* OR parole* OR "community supervis*" OR "technical violat*" OR "drug court*" OR "special* court*" OR "treatment court*" ) ) AND ( TI ( "comparison condition*" OR "comparison group*" OR "control group*" OR "control condition" OR crossover OR effecti* OR efficac* OR evaluat* OR experiment* OR interven* OR match* OR metaanaly* OR pilot* OR placebo* OR program* OR “propensity score*” OR random* OR RCT OR review* OR service* OR therap* OR train* OR treat* OR trial* ) OR AB ("comparison condition*" OR "comparison group*" OR "control group*" OR "control condition" OR crossover OR effecti* OR efficac* OR evaluat* OR experiment* OR interven* OR match* OR metaanaly* OR pilot* OR placebo* OR program* OR “propensity score*” OR random* OR RCT OR review* OR service* OR therap* OR train* OR treat* OR trial* ) OR KW ( "comparison condition*" OR "comparison group*" OR "control group*" OR "control condition" OR crossover OR effecti* OR efficac* OR evaluat* OR experiment* OR interven* OR match* OR metaanaly* OR pilot* OR placebo* OR program* OR “propensity score*” OR random* OR RCT OR review* OR service* OR therap* OR train* OR treat* OR trial* ) OR SU ( "comparison condition*" OR "comparison group*" OR "control group*" OR "control condition" OR crossover OR effecti* OR efficac* OR evaluat* OR experiment* OR interven* OR match* OR metaanaly* OR pilot* OR placebo* OR program* OR “propensity score*” OR random* OR RCT OR review* OR service* OR therap* OR train* OR treat* OR trial*) ) | Apply equivalent subjects, Publication Date: 19600101-20201031, Search modes - Boolean/Phrase |
| Omnifile Full Text Mega  EBSCO  5/7/21 | ( TI ( "Medication assisted treat*" OR "Medication assisted therap*" OR drug* OR "therapeutic use*" OR opioid* OR "replacement therap*" OR "substitution therap*" OR pharmacotherap* OR "pharmacological treatment*" OR addict* OR agonist* OR "partial agonist*" OR methadone OR methadose OR dolophine OR buprenorphine OR suboxone OR sublocade OR naltrexone OR depade OR vivitrol OR revia OR "levomethadyl acetate" OR LAAM OR orlaam OR morphine OR analgesic* OR heroin OR narcotic OR heroin OR intravenous OR "dose-response relationship*" OR "drug prescription*" ) OR AB ("Medication assisted treat*" OR "Medication assisted therap*" OR drug* OR "therapeutic use*" OR opioid* OR "replacement therap*" OR "substitution therap*" OR pharmacotherap* OR "pharmacological treatment*" OR addict* OR agonist* OR "partial agonist*" OR methadone OR methadose OR dolophine OR buprenorphine OR suboxone OR sublocade OR naltrexone OR depade OR vivitrol OR revia OR "levomethadyl acetate" OR LAAM OR orlaam OR morphine OR analgesic* OR heroin OR narcotic OR heroin OR intravenous OR "dose-response relationship*" OR "drug prescription*" ) OR KW ("Medication assisted treat*" OR "Medication assisted therap*" OR drug* OR "therapeutic use*" OR opioid* OR "replacement therap*" OR "substitution therap*" OR pharmacotherap* OR "pharmacological treatment*" OR addict* OR agonist* OR "partial agonist*" OR methadone OR methadose OR dolophine OR buprenorphine OR suboxone OR sublocade OR naltrexone OR depade OR vivitrol OR revia OR "levomethadyl acetate" OR LAAM OR orlaam OR morphine OR analgesic* OR heroin OR narcotic OR heroin OR intravenous OR "dose-response relationship*" OR "drug prescription*") OR SU ("Medication assisted treat*" OR "Medication assisted therap*" OR drug* OR "therapeutic use*" OR opioid* OR "replacement therap*" OR "substitution therap*" OR pharmacotherap* OR "pharmacological treatment*" OR addict* OR agonist* OR "partial agonist*" OR methadone OR methadose OR dolophine OR buprenorphine OR suboxone OR sublocade OR naltrexone OR depade OR vivitrol OR revia OR "levomethadyl acetate" OR LAAM OR orlaam OR morphine OR analgesic* OR heroin OR narcotic OR heroin OR intravenous OR "dose-response relationship*" OR "drug prescription*" ) ) AND ( TI ( crim* OR incarcerat* OR convict* OR offend* OR offence* OR offense* OR reincarceration OR reconvict* OR reoffen* OR recidiv* OR rearrest* OR arrest* OR probation* OR parole* OR "community supervis*" OR "technical violat*" OR "drug court*" OR "special* court*" OR "treatment court*" ) OR AB ( crim* OR incarcerat* OR convict* OR offend* OR offence* OR offense* OR reincarceration OR reconvict* OR reoffen* OR recidiv* OR rearrest* OR arrest* OR probation* OR parole* OR "community supervis*" OR "technical violat*" OR "drug court*" OR "special* court*" OR "treatment court*" ) OR KW ( crim* OR incarcerat* OR convict* OR offend* OR offence* OR offense* OR reincarceration OR reconvict* OR reoffen* OR recidiv* OR rearrest* OR arrest* OR probation* OR parole* OR "community supervis*" OR "technical violat*" OR "drug court*" OR "special* court*" OR "treatment court*" ) OR SU ( crim* OR incarcerat* OR convict* OR offend* OR offence* OR offense* OR reincarceration OR reconvict* OR reoffen* OR recidiv* OR rearrest* OR arrest* OR probation* OR parole* OR "community supervis*" OR "technical violat*" OR "drug court*" OR "special* court*" OR "treatment court*" ) ) AND ( TI (overdos*) OR AB (overdos*) OR KW (overdos*) OR SU (overdos*) ) | Apply equivalent subjects, Publication Date: 19600101-20201031, Search modes - Boolean/Phrase |
| APA PsycINFO  EBSCO  6/7/21 | ( TI ( "Medication assisted treat*" OR "Medication assisted therap*" OR drug* OR "therapeutic use*" OR opioid* OR "replacement therap*" OR "substitution therap*" OR pharmacotherap* OR "pharmacological treatment*" OR addict* OR agonist* OR "partial agonist*" OR methadone OR methadose OR dolophine OR buprenorphine OR suboxone OR sublocade OR naltrexone OR depade OR vivitrol OR revia OR "levomethadyl acetate" OR LAAM OR orlaam OR morphine OR analgesic* OR heroin OR narcotic OR heroin OR intravenous OR "dose-response relationship*" OR "drug prescription*" ) OR AB ("Medication assisted treat*" OR "Medication assisted therap*" OR drug* OR "therapeutic use*" OR opioid* OR "replacement therap*" OR "substitution therap*" OR pharmacotherap* OR "pharmacological treatment*" OR addict* OR agonist* OR "partial agonist*" OR methadone OR methadose OR dolophine OR buprenorphine OR suboxone OR sublocade OR naltrexone OR depade OR vivitrol OR revia OR "levomethadyl acetate" OR LAAM OR orlaam OR morphine OR analgesic* OR heroin OR narcotic OR heroin OR intravenous OR "dose-response relationship*" OR "drug prescription*" ) OR SU ("Medication assisted treat*" OR "Medication assisted therap*" OR drug* OR "therapeutic use*" OR opioid* OR "replacement therap*" OR "substitution therap*" OR pharmacotherap* OR "pharmacological treatment*" OR addict* OR agonist* OR "partial agonist*" OR methadone OR methadose OR dolophine OR buprenorphine OR suboxone OR sublocade OR naltrexone OR depade OR vivitrol OR revia OR "levomethadyl acetate" OR LAAM OR orlaam OR morphine OR analgesic* OR heroin OR narcotic OR heroin OR intravenous OR "dose-response relationship*" OR "drug prescription*" ) OR MJ ("Medication assisted treat*" OR "Medication assisted therap*" OR drug* OR "therapeutic use*" OR opioid* OR "replacement therap*" OR "substitution therap*" OR pharmacotherap* OR "pharmacological treatment*" OR addict* OR agonist* OR "partial agonist*" OR methadone OR methadose OR dolophine OR buprenorphine OR suboxone OR sublocade OR naltrexone OR depade OR vivitrol OR revia OR "levomethadyl acetate" OR LAAM OR orlaam OR morphine OR analgesic* OR heroin OR narcotic OR heroin OR intravenous OR "dose-response relationship*" OR "drug prescription*" ) ) AND ( TI ( crim* OR incarcerat* OR convict* OR offend* OR offence* OR offense* OR reincarceration OR reconvict* OR reoffen* OR recidiv* OR rearrest* OR arrest* OR probation* OR parole* OR "community supervis*" OR "technical violat*" OR "drug court*" OR "special* court*" OR "treatment court*" ) OR AB ( crim* OR incarcerat* OR convict* OR offend* OR offence* OR offense* OR reincarceration OR reconvict* OR reoffen* OR recidiv* OR rearrest* OR arrest* OR probation* OR parole* OR "community supervis*" OR "technical violat*" OR "drug court*" OR "special* court*" OR "treatment court*" ) OR SU (crim* OR incarcerat* OR convict* OR offend* OR offence* OR offense* OR reincarceration OR reconvict* OR reoffen* OR recidiv* OR rearrest* OR arrest* OR probation* OR parole* OR "community supervis*" OR "technical violat*" OR "drug court*" OR "special* court*" OR "treatment court*" ) OR MJ ((crim* OR incarcerat* OR convict* OR offend* OR offence* OR offense* OR reincarceration OR reconvict* OR reoffen* OR recidiv* OR rearrest* OR arrest* OR probation* OR parole* OR "community supervis*" OR "technical violat*" OR "drug court*" OR "special* court*" OR "treatment court*" ) ) AND ( TI ( "comparison condition*" OR "comparison group*" OR "control group*" OR "control condition" OR crossover OR effecti* OR efficac* OR evaluat* OR experiment* OR interven* OR match* OR metaanaly* OR pilot* OR placebo* OR program* OR “propensity score*” OR random* OR RCT OR review* OR service* OR therap* OR train* OR treat* OR trial* ) OR AB ("comparison condition*" OR "comparison group*" OR "control group*" OR "control condition" OR crossover OR effecti* OR efficac* OR evaluat* OR experiment* OR interven* OR match* OR metaanaly* OR pilot* OR placebo* OR program* OR “propensity score*” OR random* OR RCT OR review* OR service* OR therap* OR train* OR treat* OR trial* ) OR SU ( "comparison condition*" OR "comparison group*" OR "control group*" OR "control condition" OR crossover OR effecti* OR efficac* OR evaluat* OR experiment* OR interven* OR match* OR metaanaly* OR pilot* OR placebo* OR program* OR “propensity score*” OR random* OR RCT OR review* OR service* OR therap* OR train* OR treat* OR trial*) OR MJ ( "comparison condition*" OR "comparison group*" OR "control group*" OR "control condition" OR crossover OR effecti* OR efficac* OR evaluat* OR experiment* OR interven* OR match* OR metaanaly* OR pilot* OR placebo* OR program* OR “propensity score*” OR random* OR RCT OR review* OR service* OR therap* OR train* OR treat* OR trial*) ) | Apply equivalent subjects, Publication Date: 19600101-20201031, Search modes - Boolean/Phrase, Language: English |
| APA PsycINFO  EBSCO  5/9/21 | ( TI ( "Medication assisted treat*" OR "Medication assisted therap*" OR drug* OR "therapeutic use*" OR opioid* OR "replacement therap*" OR "substitution therap*" OR pharmacotherap* OR "pharmacological treatment*" OR addict* OR agonist* OR "partial agonist*" OR methadone OR methadose OR dolophine OR buprenorphine OR suboxone OR sublocade OR naltrexone OR depade OR vivitrol OR revia OR "levomethadyl acetate" OR LAAM OR orlaam OR morphine OR analgesic* OR heroin OR narcotic OR heroin OR intravenous OR "dose-response relationship*" OR "drug prescription*" "Medication assisted treat*" OR "Medication assisted therap*" OR drug* OR "therapeutic use*" OR opioid* OR "replacement therap*" OR "substitution therap*" OR pharmacotherap* OR "pharmacological treatment*" OR addict* OR agonist* OR "partial agonist*" OR methadone OR methadose OR dolophine OR buprenorphine OR suboxone OR sublocade OR naltrexone OR depade OR vivitrol OR revia OR "levomethadyl acetate" OR LAAM OR orlaam OR morphine OR analgesic* OR heroin OR narcotic OR heroin OR intravenous OR "dose-response relationship*" OR "drug prescription*" ) OR AB ( "Medication assisted treat*" OR "Medication assisted therap*" OR drug* OR "therapeutic use*" OR opioid* OR "replacement therap*" OR "substitution therap*" OR pharmacotherap* OR "pharmacological treatment*" OR addict* OR agonist* OR "partial agonist*" OR methadone OR methadose OR dolophine OR buprenorphine OR suboxone OR sublocade OR naltrexone OR depade OR vivitrol OR revia OR "levomethadyl acetate" OR LAAM OR orlaam OR morphine OR analgesic* OR heroin OR narcotic OR heroin OR intravenous OR "dose-response relationship*" OR "drug prescription*" "Medication assisted treat*" OR "Medication assisted therap*" OR drug* OR "therapeutic use*" OR opioid* OR "replacement therap*" OR "substitution therap*" OR pharmacotherap* OR "pharmacological treatment*" OR addict* OR agonist* OR "partial agonist*" OR methadone OR methadose OR dolophine OR buprenorphine OR suboxone OR sublocade OR naltrexone OR depade OR vivitrol OR revia OR "levomethadyl acetate" OR LAAM OR orlaam OR morphine OR analgesic* OR heroin OR narcotic OR heroin OR intravenous OR "dose-response relationship*" OR "drug prescription*" ) OR SU ( "Medication assisted treat*" OR "Medication assisted therap*" OR drug* OR "therapeutic use*" OR opioid* OR "replacement therap*" OR "substitution therap*" OR pharmacotherap* OR "pharmacological treatment*" OR addict* OR agonist* OR "partial agonist*" OR methadone OR methadose OR dolophine OR buprenorphine OR suboxone OR sublocade OR naltrexone OR depade OR vivitrol OR revia OR "levomethadyl acetate" OR LAAM OR orlaam OR morphine OR analgesic* OR heroin OR narcotic OR heroin OR intravenous OR "dose-response relationship*" OR "drug prescription*" "Medication assisted treat*" OR "Medication assisted therap*" OR drug* OR "therapeutic use*" OR opioid* OR "replacement therap*" OR "substitution therap*" OR pharmacotherap* OR "pharmacological treatment*" OR addict* OR agonist* OR "partial agonist*" OR methadone OR methadose OR dolophine OR buprenorphine OR suboxone OR sublocade OR naltrexone OR depade OR vivitrol OR revia OR "levomethadyl acetate" OR LAAM OR orlaam OR morphine OR analgesic* OR heroin OR narcotic OR heroin OR intravenous OR "dose-response relationship*" OR "drug prescription*" ) ) AND ( TI ( crim* OR incarcerat* OR convict* OR offend* OR offence* OR offense* OR reincarceration OR reconvict* OR reoffen* OR recidiv* OR rearrest* OR arrest* OR probation* OR parole* OR "community supervis*" OR "technical violat*" OR "drug court*" OR "special* court*" OR "treatment court*" ) OR AB ( crim* OR incarcerat* OR convict* OR offend* OR offence* OR offense* OR reincarceration OR reconvict* OR reoffen* OR recidiv* OR rearrest* OR arrest* OR probation* OR parole* OR "community supervis*" OR "technical violat*" OR "drug court*" OR "special* court*" OR "treatment court*" ) OR SU ( crim* OR incarcerat* OR convict* OR offend* OR offence* OR offense* OR reincarceration OR reconvict* OR reoffen* OR recidiv* OR rearrest* OR arrest* OR probation* OR parole* OR "community supervis*" OR "technical violat*" OR "drug court*" OR "special* court*" OR "treatment court*" ) ) AND ( TI (overdos*) OR AB (overdos*) OR KW (overdos*) OR SU (overdos*) ) | Apply equivalent subjects, Publication Date: 19600101-20201031, Search modes - Boolean/Phrase, Language: English |
| Social Work Abstracts  EBSCO  5/15/21 | ( TI ( "Medication assisted treat*" OR "Medication assisted therap*" OR drug* OR "therapeutic use*" OR opioid* OR "replacement therap*" OR "substitution therap*" OR pharmacotherap* OR "pharmacological treatment*" OR addict* OR agonist* OR "partial agonist*" OR methadone OR methadose OR dolophine OR buprenorphine OR suboxone OR sublocade OR naltrexone OR depade OR vivitrol OR revia OR "levomethadyl acetate" OR LAAM OR orlaam OR morphine OR analgesic* OR heroin OR narcotic OR heroin OR intravenous OR "dose-response relationship*" OR "drug prescription*" ) OR AB ("Medication assisted treat*" OR "Medication assisted therap*" OR drug* OR "therapeutic use*" OR opioid* OR "replacement therap*" OR "substitution therap*" OR pharmacotherap* OR "pharmacological treatment*" OR addict* OR agonist* OR "partial agonist*" OR methadone OR methadose OR dolophine OR buprenorphine OR suboxone OR sublocade OR naltrexone OR depade OR vivitrol OR revia OR "levomethadyl acetate" OR LAAM OR orlaam OR morphine OR analgesic* OR heroin OR narcotic OR heroin OR intravenous OR "dose-response relationship*" OR "drug prescription*" ) OR SU ("Medication assisted treat*" OR "Medication assisted therap*" OR drug* OR "therapeutic use*" OR opioid* OR "replacement therap*" OR "substitution therap*" OR pharmacotherap* OR "pharmacological treatment*" OR addict* OR agonist* OR "partial agonist*" OR methadone OR methadose OR dolophine OR buprenorphine OR suboxone OR sublocade OR naltrexone OR depade OR vivitrol OR revia OR "levomethadyl acetate" OR LAAM OR orlaam OR morphine OR analgesic* OR heroin OR narcotic OR heroin OR intravenous OR "dose-response relationship*" OR "drug prescription*" ) ) AND ( TI ( crim* OR incarcerat* OR convict* OR offend* OR offence* OR offense* OR reincarceration OR reconvict* OR reoffen* OR recidiv* OR rearrest* OR arrest* OR probation* OR parole* OR "community supervis*" OR "technical violat*" OR "drug court*" OR "special* court*" OR "treatment court*" ) OR AB ( crim* OR incarcerat* OR convict* OR offend* OR offence* OR offense* OR reincarceration OR reconvict* OR reoffen* OR recidiv* OR rearrest* OR arrest* OR probation* OR parole* OR "community supervis*" OR "technical violat*" OR "drug court*" OR "special* court*" OR "treatment court*" ) OR SU (crim* OR incarcerat* OR convict* OR offend* OR offence* OR offense* OR reincarceration OR reconvict* OR reoffen* OR recidiv* OR rearrest* OR arrest* OR probation* OR parole* OR "community supervis*" OR "technical violat*" OR "drug court*" OR "special* court*" OR "treatment court*" ) ) AND ( TI ( "comparison condition*" OR "comparison group*" OR "control group*" OR "control condition" OR crossover OR effecti* OR efficac* OR evaluat* OR experiment* OR interven* OR match* OR metaanaly* OR pilot* OR placebo* OR program* OR “propensity score*” OR random* OR RCT OR review* OR service* OR therap* OR train* OR treat* OR trial* ) OR AB ("comparison condition*" OR "comparison group*" OR "control group*" OR "control condition" OR crossover OR effecti* OR efficac* OR evaluat* OR experiment* OR interven* OR match* OR metaanaly* OR pilot* OR placebo* OR program* OR “propensity score*” OR random* OR RCT OR review* OR service* OR therap* OR train* OR treat* OR trial* ) OR SU ( "comparison condition*" OR "comparison group*" OR "control group*" OR "control condition" OR crossover OR effecti* OR efficac* OR evaluat* OR experiment* OR interven* OR match* OR metaanaly* OR pilot* OR placebo* OR program* OR “propensity score*” OR random* OR RCT OR review* OR service* OR therap* OR train* OR treat* OR trial*) ) | Apply equivalent subjects, Publication Date: 19600101-20201031, Search modes - Boolean/Phrase |
| Social Work Abstracts  EBSCO  5/9/21 | ( TI ( "Medication assisted treat*" OR "Medication assisted therap*" OR drug* OR "therapeutic use*" OR opioid* OR "replacement therap*" OR "substitution therap*" OR pharmacotherap* OR "pharmacological treatment*" OR addict* OR agonist* OR "partial agonist*" OR methadone OR methadose OR dolophine OR buprenorphine OR suboxone OR sublocade OR naltrexone OR depade OR vivitrol OR revia OR "levomethadyl acetate" OR LAAM OR orlaam OR morphine OR analgesic* OR heroin OR narcotic OR heroin OR intravenous OR "dose-response relationship*" OR "drug prescription*") OR AB ( "Medication assisted treat*" OR "Medication assisted therap*" OR drug* OR "therapeutic use*" OR opioid* OR "replacement therap*" OR "substitution therap*" OR pharmacotherap* OR "pharmacological treatment*" OR addict* OR agonist* OR "partial agonist*" OR methadone OR methadose OR dolophine OR buprenorphine OR suboxone OR sublocade OR naltrexone OR depade OR vivitrol OR revia OR "levomethadyl acetate" OR LAAM OR orlaam OR morphine OR analgesic* OR heroin OR narcotic OR heroin OR intravenous OR "dose-response relationship*" OR "drug prescription*") OR SU ( "Medication assisted treat*" OR "Medication assisted therap*" OR drug* OR "therapeutic use*" OR opioid* OR "replacement therap*" OR "substitution therap*" OR pharmacotherap* OR "pharmacological treatment*" OR addict* OR agonist* OR "partial agonist*" OR methadone OR methadose OR dolophine OR buprenorphine OR suboxone OR sublocade OR naltrexone OR depade OR vivitrol OR revia OR "levomethadyl acetate" OR LAAM OR orlaam OR morphine OR analgesic* OR heroin OR narcotic OR heroin OR intravenous OR "dose-response relationship*" OR "drug prescription*") ) AND ( TI ( crim* OR incarcerat* OR convict* OR offend* OR offence* OR offense* OR reincarceration OR reconvict* OR reoffen* OR recidiv* OR rearrest* OR arrest* OR probation* OR parole* OR "community supervis*" OR "technical violat*" OR "drug court*" OR "special* court*" OR "treatment court*" ) OR AB ( crim* OR incarcerat* OR convict* OR offend* OR offence* OR offense* OR reincarceration OR reconvict* OR reoffen* OR recidiv* OR rearrest* OR arrest* OR probation* OR parole* OR "community supervis*" OR "technical violat*" OR "drug court*" OR "special* court*" OR "treatment court*" ) OR SU ( crim* OR incarcerat* OR convict* OR offend* OR offence* OR offense* OR reincarceration OR reconvict* OR reoffen* OR recidiv* OR rearrest* OR arrest* OR probation* OR parole* OR "community supervis*" OR "technical violat*" OR "drug court*" OR "special* court*" OR "treatment court*" ) ) AND ( TI (overdos*) OR AB (overdos*) OR SU (overdos*) ) | Apply equivalent subjects, Publication Date: 19600101-20201031, Search modes - Boolean/Phrase |
| Women's Studies International  EBSCO  5/14/21 | ( TI ( "Medication assisted treat*" OR "Medication assisted therap*" OR drug* OR "therapeutic use*" OR opioid* OR "replacement therap*" OR "substitution therap*" OR pharmacotherap* OR "pharmacological treatment*" OR addict* OR agonist* OR "partial agonist*" OR methadone OR methadose OR dolophine OR buprenorphine OR suboxone OR sublocade OR naltrexone OR depade OR vivitrol OR revia OR "levomethadyl acetate" OR LAAM OR orlaam OR morphine OR analgesic* OR heroin OR narcotic OR heroin OR intravenous OR "dose-response relationship*" OR "drug prescription*" ) OR AB ("Medication assisted treat*" OR "Medication assisted therap*" OR drug* OR "therapeutic use*" OR opioid* OR "replacement therap*" OR "substitution therap*" OR pharmacotherap* OR "pharmacological treatment*" OR addict* OR agonist* OR "partial agonist*" OR methadone OR methadose OR dolophine OR buprenorphine OR suboxone OR sublocade OR naltrexone OR depade OR vivitrol OR revia OR "levomethadyl acetate" OR LAAM OR orlaam OR morphine OR analgesic* OR heroin OR narcotic OR heroin OR intravenous OR "dose-response relationship*" OR "drug prescription*" ) OR KW ("Medication assisted treat*" OR "Medication assisted therap*" OR drug* OR "therapeutic use*" OR opioid* OR "replacement therap*" OR "substitution therap*" OR pharmacotherap* OR "pharmacological treatment*" OR addict* OR agonist* OR "partial agonist*" OR methadone OR methadose OR dolophine OR buprenorphine OR suboxone OR sublocade OR naltrexone OR depade OR vivitrol OR revia OR "levomethadyl acetate" OR LAAM OR orlaam OR morphine OR analgesic* OR heroin OR narcotic OR heroin OR intravenous OR "dose-response relationship*" OR "drug prescription*") OR SU ("Medication assisted treat*" OR "Medication assisted therap*" OR drug* OR "therapeutic use*" OR opioid* OR "replacement therap*" OR "substitution therap*" OR pharmacotherap* OR "pharmacological treatment*" OR addict* OR agonist* OR "partial agonist*" OR methadone OR methadose OR dolophine OR buprenorphine OR suboxone OR sublocade OR naltrexone OR depade OR vivitrol OR revia OR "levomethadyl acetate" OR LAAM OR orlaam OR morphine OR analgesic* OR heroin OR narcotic OR heroin OR intravenous OR "dose-response relationship*" OR "drug prescription*" ) ) AND ( TI ( crim* OR incarcerat* OR convict* OR offend* OR offence* OR offense* OR reincarceration OR reconvict* OR reoffen* OR recidiv* OR rearrest* OR arrest* OR probation* OR parole* OR "community supervis*" OR "technical violat*" OR "drug court*" OR "special* court*" OR "treatment court*" ) OR AB ( crim* OR incarcerat* OR convict* OR offend* OR offence* OR offense* OR reincarceration OR reconvict* OR reoffen* OR recidiv* OR rearrest* OR arrest* OR probation* OR parole* OR "community supervis*" OR "technical violat*" OR "drug court*" OR "special* court*" OR "treatment court*" ) OR KW ( crim* OR incarcerat* OR convict* OR offend* OR offence* OR offense* OR reincarceration OR reconvict* OR reoffen* OR recidiv* OR rearrest* OR arrest* OR probation* OR parole* OR "community supervis*" OR "technical violat*" OR "drug court*" OR "special* court*" OR "treatment court*" ) OR SU (crim* OR incarcerat* OR convict* OR offend* OR offence* OR offense* OR reincarceration OR reconvict* OR reoffen* OR recidiv* OR rearrest* OR arrest* OR probation* OR parole* OR "community supervis*" OR "technical violat*" OR "drug court*" OR "special* court*" OR "treatment court*" ) ) AND ( TI ( "comparison condition*" OR "comparison group*" OR "control group*" OR "control condition" OR crossover OR effecti* OR efficac* OR evaluat* OR experiment* OR interven* OR match* OR metaanaly* OR pilot* OR placebo* OR program* OR “propensity score*” OR random* OR RCT OR review* OR service* OR therap* OR train* OR treat* OR trial* ) OR AB ("comparison condition*" OR "comparison group*" OR "control group*" OR "control condition" OR crossover OR effecti* OR efficac* OR evaluat* OR experiment* OR interven* OR match* OR metaanaly* OR pilot* OR placebo* OR program* OR “propensity score*” OR random* OR RCT OR review* OR service* OR therap* OR train* OR treat* OR trial* ) OR KW ( "comparison condition*" OR "comparison group*" OR "control group*" OR "control condition" OR crossover OR effecti* OR efficac* OR evaluat* OR experiment* OR interven* OR match* OR metaanaly* OR pilot* OR placebo* OR program* OR “propensity score*” OR random* OR RCT OR review* OR service* OR therap* OR train* OR treat* OR trial* ) OR SU ( "comparison condition*" OR "comparison group*" OR "control group*" OR "control condition" OR crossover OR effecti* OR efficac* OR evaluat* OR experiment* OR interven* OR match* OR metaanaly* OR pilot* OR placebo* OR program* OR “propensity score*” OR random* OR RCT OR review* OR service* OR therap* OR train* OR treat* OR trial*) ) | Apply equivalent subjects, Publication Date: 19600101-20201031, Search modes - Boolean/Phrase |
| Women's Studies International  EBSCO  5/9/21 | ( TI ( "Medication assisted treat*" OR "Medication assisted therap*" OR drug* OR "therapeutic use*" OR opioid* OR "replacement therap*" OR "substitution therap*" OR pharmacotherap* OR "pharmacological treatment*" OR addict* OR agonist* OR "partial agonist*" OR methadone OR methadose OR dolophine OR buprenorphine OR suboxone OR sublocade OR naltrexone OR depade OR vivitrol OR revia OR "levomethadyl acetate" OR LAAM OR orlaam OR morphine OR analgesic* OR heroin OR narcotic OR heroin OR intravenous OR "dose-response relationship*" OR "drug prescription*" "Medication assisted treat*" OR "Medication assisted therap*" OR drug* OR "therapeutic use*" OR opioid* OR "replacement therap*" OR "substitution therap*" OR pharmacotherap* OR "pharmacological treatment*" OR addict* OR agonist* OR "partial agonist*" OR methadone OR methadose OR dolophine OR buprenorphine OR suboxone OR sublocade OR naltrexone OR depade OR vivitrol OR revia OR "levomethadyl acetate" OR LAAM OR orlaam OR morphine OR analgesic* OR heroin OR narcotic OR heroin OR intravenous OR "dose-response relationship*" OR "drug prescription*" ) OR AB ( "Medication assisted treat*" OR "Medication assisted therap*" OR drug* OR "therapeutic use*" OR opioid* OR "replacement therap*" OR "substitution therap*" OR pharmacotherap* OR "pharmacological treatment*" OR addict* OR agonist* OR "partial agonist*" OR methadone OR methadose OR dolophine OR buprenorphine OR suboxone OR sublocade OR naltrexone OR depade OR vivitrol OR revia OR "levomethadyl acetate" OR LAAM OR orlaam OR morphine OR analgesic* OR heroin OR narcotic OR heroin OR intravenous OR "dose-response relationship*" OR "drug prescription*" "Medication assisted treat*" OR "Medication assisted therap*" OR drug* OR "therapeutic use*" OR opioid* OR "replacement therap*" OR "substitution therap*" OR pharmacotherap* OR "pharmacological treatment*" OR addict* OR agonist* OR "partial agonist*" OR methadone OR methadose OR dolophine OR buprenorphine OR suboxone OR sublocade OR naltrexone OR depade OR vivitrol OR revia OR "levomethadyl acetate" OR LAAM OR orlaam OR morphine OR analgesic* OR heroin OR narcotic OR heroin OR intravenous OR "dose-response relationship*" OR "drug prescription*" ) OR KW ( "Medication assisted treat*" OR "Medication assisted therap*" OR drug* OR "therapeutic use*" OR opioid* OR "replacement therap*" OR "substitution therap*" OR pharmacotherap* OR "pharmacological treatment*" OR addict* OR agonist* OR "partial agonist*" OR methadone OR methadose OR dolophine OR buprenorphine OR suboxone OR sublocade OR naltrexone OR depade OR vivitrol OR revia OR "levomethadyl acetate" OR LAAM OR orlaam OR morphine OR analgesic* OR heroin OR narcotic OR heroin OR intravenous OR "dose-response relationship*" OR "drug prescription*" "Medication assisted treat*" OR "Medication assisted therap*" OR drug* OR "therapeutic use*" OR opioid* OR "replacement therap*" OR "substitution therap*" OR pharmacotherap* OR "pharmacological treatment*" OR addict* OR agonist* OR "partial agonist*" OR methadone OR methadose OR dolophine OR buprenorphine OR suboxone OR sublocade OR naltrexone OR depade OR vivitrol OR revia OR "levomethadyl acetate" OR LAAM OR orlaam OR morphine OR analgesic* OR heroin OR narcotic OR heroin OR intravenous OR "dose-response relationship*" OR "drug prescription*" ) OR SU ( "Medication assisted treat*" OR "Medication assisted therap*" OR drug* OR "therapeutic use*" OR opioid* OR "replacement therap*" OR "substitution therap*" OR pharmacotherap* OR "pharmacological treatment*" OR addict* OR agonist* OR "partial agonist*" OR methadone OR methadose OR dolophine OR buprenorphine OR suboxone OR sublocade OR naltrexone OR depade OR vivitrol OR revia OR "levomethadyl acetate" OR LAAM OR orlaam OR morphine OR analgesic* OR heroin OR narcotic OR heroin OR intravenous OR "dose-response relationship*" OR "drug prescription*" "Medication assisted treat*" OR "Medication assisted therap*" OR drug* OR "therapeutic use*" OR opioid* OR "replacement therap*" OR "substitution therap*" OR pharmacotherap* OR "pharmacological treatment*" OR addict* OR agonist* OR "partial agonist*" OR methadone OR methadose OR dolophine OR buprenorphine OR suboxone OR sublocade OR naltrexone OR depade OR vivitrol OR revia OR "levomethadyl acetate" OR LAAM OR orlaam OR morphine OR analgesic* OR heroin OR narcotic OR heroin OR intravenous OR "dose-response relationship*" OR "drug prescription*" ) ) AND ( TI ( crim* OR incarcerat* OR convict* OR offend* OR offence* OR offense* OR reincarceration OR reconvict* OR reoffen* OR recidiv* OR rearrest* OR arrest* OR probation* OR parole* OR "community supervis*" OR "technical violat*" OR "drug court*" OR "special* court*" OR "treatment court*" ) OR AB ( crim* OR incarcerat* OR convict* OR offend* OR offence* OR offense* OR reincarceration OR reconvict* OR reoffen* OR recidiv* OR rearrest* OR arrest* OR probation* OR parole* OR "community supervis*" OR "technical violat*" OR "drug court*" OR "special* court*" OR "treatment court*" ) OR KW ( crim* OR incarcerat* OR convict* OR offend* OR offence* OR offense* OR reincarceration OR reconvict* OR reoffen* OR recidiv* OR rearrest* OR arrest* OR probation* OR parole* OR "community supervis*" OR "technical violat*" OR "drug court*" OR "special* court*" OR "treatment court*" ) OR SU ( crim* OR incarcerat* OR convict* OR offend* OR offence* OR offense* OR reincarceration OR reconvict* OR reoffen* OR recidiv* OR rearrest* OR arrest* OR probation* OR parole* OR "community supervis*" OR "technical violat*" OR "drug court*" OR "special* court*" OR "treatment court*" ) ) AND ( TI (overdos*) OR AB (overdos*) OR KW (overdos*) OR SU (overdos*) ) | Apply equivalent subjects, Publication Date: 19600101-20201031, Search modes - Boolean/Phrase |
| Criminal Justice Database  ProQuest  6/7/21 | (ti("Medication assisted treat*" OR "Medication assisted therap*" OR drug* OR "therapeutic use*" OR opioid* OR "replacement therap*" OR "substitution therap*" OR pharmacotherap* OR "pharmacological treatment*" OR addict* OR agonist* OR "partial agonist*" OR methadone OR methadose OR dolophine OR buprenorphine OR suboxone OR sublocade OR naltrexone OR depade OR vivitrol OR revia OR "levomethadyl acetate" OR LAAM OR orlaam OR morphine OR analgesic* OR heroin OR narcotic OR heroin OR intravenous OR "dose-response relationship*" OR "drug prescription*") OR ab("Medication assisted treat*" OR "Medication assisted therap*" OR drug* OR "therapeutic use*" OR opioid* OR "replacement therap*" OR "substitution therap*" OR pharmacotherap* OR "pharmacological treatment*" OR addict* OR agonist* OR "partial agonist*" OR methadone OR methadose OR dolophine OR buprenorphine OR suboxone OR sublocade OR naltrexone OR depade OR vivitrol OR revia OR "levomethadyl acetate" OR LAAM OR orlaam OR morphine OR analgesic* OR heroin OR narcotic OR heroin OR intravenous OR "dose-response relationship*" OR "drug prescription*")) AND (ti(crim* OR incarcerat* OR convict* OR offend* OR offence* OR offense* OR reincarceration OR reconvict* OR reoffen* OR recidiv* OR rearrest* OR arrest* OR probation* OR parole* OR "community supervis*" OR "technical violat*" OR "drug court*" OR "special* court*" OR "treatment court*") OR ab(crim* OR incarcerat* OR convict* OR offend* OR offence* OR offense* OR reincarceration OR reconvict* OR reoffen* OR recidiv* OR rearrest* OR arrest* OR probation* OR parole* OR "community supervis*" OR "technical violat*" OR "drug court*" OR "special* court*" OR "treatment court*")) AND (ti("comparison condition*" OR "comparison group*" OR "control group*" OR "control condition" OR crossover OR effecti* OR efficac* OR evaluat* OR experiment* OR interven* OR match* OR metaanaly* OR pilot* OR placebo* OR program* OR "propensity score*" OR random* OR RCT OR review* OR service* OR therap* OR train* OR treat* OR trial*) OR ab("comparison condition*" OR "comparison group*" OR "control group*" OR "control condition" OR crossover OR effecti* OR efficac* OR evaluat* OR experiment* OR interven* OR match* OR metaanaly* OR pilot* OR placebo* OR program* OR "propensity score*" OR random* OR RCT OR review* OR service* OR therap* OR train* OR treat* OR trial*)) | Dates of Publication: January 1, 1960 - October 31, 2020, Language: English, Removed 'Source Type' (Blogs, Podcasts, and Websites, and Wire Feeds), Removed 'Document type' (Advertisements, Blog, Correspondence, Editorial, Editorial Cartoon, General Information, Image/photograph, Interview, News, Obituary, Poem, Prose, Recipe), Search modes - Boolean/Phrase |
| Criminal Justice Database  ProQuest  5/9/21 | (ti("Medication assisted treat*" OR "Medication assisted therap*" OR drug* OR "therapeutic use*" OR opioid* OR "replacement therap*" OR "substitution therap*" OR pharmacotherap* OR "pharmacological treatment*" OR addict* OR agonist* OR "partial agonist*" OR methadone OR methadose OR dolophine OR buprenorphine OR suboxone OR sublocade OR naltrexone OR depade OR vivitrol OR revia OR "levomethadyl acetate" OR LAAM OR orlaam OR morphine OR analgesic* OR heroin OR narcotic OR heroin OR intravenous OR "dose-response relationship*" OR "drug prescription*") OR ab("Medication assisted treat*" OR "Medication assisted therap*" OR drug* OR "therapeutic use*" OR opioid* OR "replacement therap*" OR "substitution therap*" OR pharmacotherap* OR "pharmacological treatment*" OR addict* OR agonist* OR "partial agonist*" OR methadone OR methadose OR dolophine OR buprenorphine OR suboxone OR sublocade OR naltrexone OR depade OR vivitrol OR revia OR "levomethadyl acetate" OR LAAM OR orlaam OR morphine OR analgesic* OR heroin OR narcotic OR heroin OR intravenous OR "dose-response relationship*" OR "drug prescription*")) AND (ti(crim* OR incarcerat* OR convict* OR offend* OR offence* OR offense* OR reincarceration OR reconvict* OR reoffen* OR recidiv* OR rearrest* OR arrest* OR probation* OR parole* OR "community supervis*" OR "technical violat*" OR "drug court*" OR "special* court*" OR "treatment court*") OR ab(crim* OR incarcerat* OR convict* OR offend* OR offence* OR offense* OR reincarceration OR reconvict* OR reoffen* OR recidiv* OR rearrest* OR arrest* OR probation* OR parole* OR "community supervis*" OR "technical violat*" OR "drug court*" OR "special* court*" OR "treatment court*")) AND (ti(overdos*) OR ab(overdos*)) | Dates of Publication: January 1, 1960 - October 31, 2020, Language: English, Removed 'Source Type' (Blogs, Podcasts, and Websites, and Wire Feeds), Removed 'Document type' (Advertisements, Blog, Correspondence, Editorial, Editorial Cartoon, General Information, Image/photograph, Interview, News, Obituary, Poem, Prose, Recipe), Search modes - Boolean/Phrase |
| PAIS  ProQuest  6/7/21 | (ti("Medication assisted treat*" OR "Medication assisted therap*" OR drug* OR "therapeutic use*" OR opioid* OR "replacement therap*" OR "substitution therap*" OR pharmacotherap* OR "pharmacological treatment*" OR addict* OR agonist* OR "partial agonist*" OR methadone OR methadose OR dolophine OR buprenorphine OR suboxone OR sublocade OR naltrexone OR depade OR vivitrol OR revia OR "levomethadyl acetate" OR LAAM OR orlaam OR morphine OR analgesic* OR heroin OR narcotic OR heroin OR intravenous OR "dose-response relationship*" OR "drug prescription*") OR ab("Medication assisted treat*" OR "Medication assisted therap*" OR drug* OR "therapeutic use*" OR opioid* OR "replacement therap*" OR "substitution therap*" OR pharmacotherap* OR "pharmacological treatment*" OR addict* OR agonist* OR "partial agonist*" OR methadone OR methadose OR dolophine OR buprenorphine OR suboxone OR sublocade OR naltrexone OR depade OR vivitrol OR revia OR "levomethadyl acetate" OR LAAM OR orlaam OR morphine OR analgesic* OR heroin OR narcotic OR heroin OR intravenous OR "dose-response relationship*" OR "drug prescription*") OR su("Medication assisted treat*" OR "Medication assisted therap*" OR drug* OR "therapeutic use*" OR opioid* OR "replacement therap*" OR "substitution therap*" OR pharmacotherap* OR "pharmacological treatment*" OR addict* OR agonist* OR "partial agonist*" OR methadone OR methadose OR dolophine OR buprenorphine OR suboxone OR sublocade OR naltrexone OR depade OR vivitrol OR revia OR "levomethadyl acetate" OR LAAM OR orlaam OR morphine OR analgesic* OR heroin OR narcotic OR heroin OR intravenous OR "dose-response relationship*" OR "drug prescription*")) AND (ti(crim* OR incarcerat* OR convict* OR offend* OR offence* OR offense* OR reincarceration OR reconvict* OR reoffen* OR recidiv* OR rearrest* OR arrest* OR probation* OR parole* OR "community supervis*" OR "technical violat*" OR "drug court*" OR "special* court*" OR "treatment court*") OR ab(crim* OR incarcerat* OR convict* OR offend* OR offence* OR offense* OR reincarceration OR reconvict* OR reoffen* OR recidiv* OR rearrest* OR arrest* OR probation* OR parole* OR "community supervis*" OR "technical violat*" OR "drug court*" OR "special* court*" OR "treatment court*") OR su(crim* OR incarcerat* OR convict* OR offend* OR offence* OR offense* OR reincarceration OR reconvict* OR reoffen* OR recidiv* OR rearrest* OR arrest* OR probation* OR parole* OR "community supervis*" OR "technical violat*" OR "drug court*" OR "special* court*" OR "treatment court*")) AND (ti("comparison condition*" OR "comparison group*" OR "control group*" OR "control condition" OR crossover OR effecti* OR efficac* OR evaluat* OR experiment* OR interven* OR match* OR metaanaly* OR pilot* OR placebo* OR program* OR "propensity score*" OR random* OR RCT OR review* OR service* OR therap* OR train* OR treat* OR trial*) OR ab("comparison condition*" OR "comparison group*" OR "control group*" OR "control condition" OR crossover OR effecti* OR efficac* OR evaluat* OR experiment* OR interven* OR match* OR metaanaly* OR pilot* OR placebo* OR program* OR "propensity score*" OR random* OR RCT OR review* OR service* OR therap* OR train* OR treat* OR trial*) OR su("comparison condition*" OR "comparison group*" OR "control group*" OR "control condition" OR crossover OR effecti* OR efficac* OR evaluat* OR experiment* OR interven* OR match* OR metaanaly* OR pilot* OR placebo* OR program* OR "propensity score*" OR random* OR RCT OR review* OR service* OR therap* OR train* OR treat* OR trial*)) | Date of publication: January 1, 1960 - October 31, 2020, Language: English, Source Type: All, Removed from 'Document Type' (Advertisement, Correspondence, Editorial, Editorial Cartoon, Fiction, General Information, Illustration, Interview, Image/Photograph, News, Obituary, Poem, Prose, Recipe), Search mode: Boolean/Phrase |
| PAIS  ProQuest  6/5/21 | (ti("Medication assisted treat*" OR "Medication assisted therap*" OR drug* OR "therapeutic use*" OR opioid* OR "replacement therap*" OR "substitution therap*" OR pharmacotherap* OR "pharmacological treatment*" OR addict* OR agonist* OR "partial agonist*" OR methadone OR methadose OR dolophine OR buprenorphine OR suboxone OR sublocade OR naltrexone OR depade OR vivitrol OR revia OR "levomethadyl acetate" OR LAAM OR orlaam OR morphine OR analgesic* OR heroin OR narcotic OR heroin OR intravenous OR "dose-response relationship*" OR "drug prescription*") OR ab("Medication assisted treat*" OR "Medication assisted therap*" OR drug* OR "therapeutic use*" OR opioid* OR "replacement therap*" OR "substitution therap*" OR pharmacotherap* OR "pharmacological treatment*" OR addict* OR agonist* OR "partial agonist*" OR methadone OR methadose OR dolophine OR buprenorphine OR suboxone OR sublocade OR naltrexone OR depade OR vivitrol OR revia OR "levomethadyl acetate" OR LAAM OR orlaam OR morphine OR analgesic* OR heroin OR narcotic OR heroin OR intravenous OR "dose-response relationship*" OR "drug prescription*") OR su("Medication assisted treat*" OR "Medication assisted therap*" OR drug* OR "therapeutic use*" OR opioid* OR "replacement therap*" OR "substitution therap*" OR pharmacotherap* OR "pharmacological treatment*" OR addict* OR agonist* OR "partial agonist*" OR methadone OR methadose OR dolophine OR buprenorphine OR suboxone OR sublocade OR naltrexone OR depade OR vivitrol OR revia OR "levomethadyl acetate" OR LAAM OR orlaam OR morphine OR analgesic* OR heroin OR narcotic OR heroin OR intravenous OR "dose-response relationship*" OR "drug prescription*")) AND (ti(crim* OR incarcerat* OR convict* OR offend* OR offence* OR offense* OR reincarceration OR reconvict* OR reoffen* OR recidiv* OR rearrest* OR arrest* OR probation* OR parole* OR "community supervis*" OR "technical violat*" OR "drug court*" OR "special* court*" OR "treatment court*") OR ab(crim* OR incarcerat* OR convict* OR offend* OR offence* OR offense* OR reincarceration OR reconvict* OR reoffen* OR recidiv* OR rearrest* OR arrest* OR probation* OR parole* OR "community supervis*" OR "technical violat*" OR "drug court*" OR "special* court*" OR "treatment court*") OR su(crim* OR incarcerat* OR convict* OR offend* OR offence* OR offense* OR reincarceration OR reconvict* OR reoffen* OR recidiv* OR rearrest* OR arrest* OR probation* OR parole* OR "community supervis*" OR "technical violat*" OR "drug court*" OR "special* court*" OR "treatment court*")) AND (ti(overdos*) OR ab(overdos*) OR su(overdos*)) | Date of publication: January 1, 1960 - October 31, 2020, Language: English, Source Type: All, Removed from 'Document Type' (Advertisement, Correspondence, Editorial, Editorial Cartoon, Fiction, General Information, Illustration, Interview, Image/Photograph, News, Obituary, Poem, Prose, Recipe), Search mode: Boolean/Phrase |
| Dissertations & Theses Global  ProQuest  6/7/21 | (ti("Medication assisted treat*" OR "Medication assisted therap*" OR drug* OR "therapeutic use*" OR opioid* OR "replacement therap*" OR "substitution therap*" OR pharmacotherap* OR "pharmacological treatment*" OR addict* OR agonist* OR "partial agonist*" OR methadone OR methadose OR dolophine OR buprenorphine OR suboxone OR sublocade OR naltrexone OR depade OR vivitrol OR revia OR "levomethadyl acetate" OR LAAM OR orlaam OR morphine OR analgesic* OR heroin OR narcotic OR heroin OR intravenous OR "dose-response relationship*" OR "drug prescription*") OR ab("Medication assisted treat*" OR "Medication assisted therap*" OR drug* OR "therapeutic use*" OR opioid* OR "replacement therap*" OR "substitution therap*" OR pharmacotherap* OR "pharmacological treatment*" OR addict* OR agonist* OR "partial agonist*" OR methadone OR methadose OR dolophine OR buprenorphine OR suboxone OR sublocade OR naltrexone OR depade OR vivitrol OR revia OR "levomethadyl acetate" OR LAAM OR orlaam OR morphine OR analgesic* OR heroin OR narcotic OR heroin OR intravenous OR "dose-response relationship*" OR "drug prescription*") OR su("Medication assisted treat*" OR "Medication assisted therap*" OR drug* OR "therapeutic use*" OR opioid* OR "replacement therap*" OR "substitution therap*" OR pharmacotherap* OR "pharmacological treatment*" OR addict* OR agonist* OR "partial agonist*" OR methadone OR methadose OR dolophine OR buprenorphine OR suboxone OR sublocade OR naltrexone OR depade OR vivitrol OR revia OR "levomethadyl acetate" OR LAAM OR orlaam OR morphine OR analgesic* OR heroin OR narcotic OR heroin OR intravenous OR "dose-response relationship*" OR "drug prescription*")) AND (ti(crim* OR incarcerat* OR convict* OR offend* OR offence* OR offense* OR reincarceration OR reconvict* OR reoffen* OR recidiv* OR rearrest* OR arrest* OR probation* OR parole* OR "community supervis*" OR "technical violat*" OR "drug court*" OR "special* court*" OR "treatment court*") OR ab(crim* OR incarcerat* OR convict* OR offend* OR offence* OR offense* OR reincarceration OR reconvict* OR reoffen* OR recidiv* OR rearrest* OR arrest* OR probation* OR parole* OR "community supervis*" OR "technical violat*" OR "drug court*" OR "special* court*" OR "treatment court*") OR su(crim* OR incarcerat* OR convict* OR offend* OR offence* OR offense* OR reincarceration OR reconvict* OR reoffen* OR recidiv* OR rearrest* OR arrest* OR probation* OR parole* OR "community supervis*" OR "technical violat*" OR "drug court*" OR "special* court*" OR "treatment court*")) AND (ti("comparison condition*" OR "comparison group*" OR "control group*" OR "control condition" OR crossover OR effecti* OR efficac* OR evaluat* OR experiment* OR interven* OR match* OR metaanaly* OR pilot* OR placebo* OR program* OR "propensity score*" OR random* OR RCT OR review* OR service* OR therap* OR train* OR treat* OR trial*) OR ab("comparison condition*" OR "comparison group*" OR "control group*" OR "control condition" OR crossover OR effecti* OR efficac* OR evaluat* OR experiment* OR interven* OR match* OR metaanaly* OR pilot* OR placebo* OR program* OR "propensity score*" OR random* OR RCT OR review* OR service* OR therap* OR train* OR treat* OR trial*) OR su("comparison condition*" OR "comparison group*" OR "control group*" OR "control condition" OR crossover OR effecti* OR efficac* OR evaluat* OR experiment* OR interven* OR match* OR metaanaly* OR pilot* OR placebo* OR program* OR "propensity score*" OR random* OR RCT OR review* OR service* OR therap* OR train* OR treat* OR trial*)) | Date of Publication: 1960-01-01 to 2020-10-31, Language: English, Search mode: Boolean/Phrase |
| Dissertations & Theses Global  ProQuest  5/11/21 | (ti("Medication assisted treat*" OR "Medication assisted therap*" OR drug* OR "therapeutic use*" OR opioid* OR "replacement therap*" OR "substitution therap*" OR pharmacotherap* OR "pharmacological treatment*" OR addict* OR agonist* OR "partial agonist*" OR methadone OR methadose OR dolophine OR buprenorphine OR suboxone OR sublocade OR naltrexone OR depade OR vivitrol OR revia OR "levomethadyl acetate" OR LAAM OR orlaam OR morphine OR analgesic* OR heroin OR narcotic OR heroin OR intravenous OR "dose-response relationship*" OR "drug prescription*") OR ab("Medication assisted treat*" OR "Medication assisted therap*" OR drug* OR "therapeutic use*" OR opioid* OR "replacement therap*" OR "substitution therap*" OR pharmacotherap* OR "pharmacological treatment*" OR addict* OR agonist* OR "partial agonist*" OR methadone OR methadose OR dolophine OR buprenorphine OR suboxone OR sublocade OR naltrexone OR depade OR vivitrol OR revia OR "levomethadyl acetate" OR LAAM OR orlaam OR morphine OR analgesic* OR heroin OR narcotic OR heroin OR intravenous OR "dose-response relationship*" OR "drug prescription*") OR su("Medication assisted treat*" OR "Medication assisted therap*" OR drug* OR "therapeutic use*" OR opioid* OR "replacement therap*" OR "substitution therap*" OR pharmacotherap* OR "pharmacological treatment*" OR addict* OR agonist* OR "partial agonist*" OR methadone OR methadose OR dolophine OR buprenorphine OR suboxone OR sublocade OR naltrexone OR depade OR vivitrol OR revia OR "levomethadyl acetate" OR LAAM OR orlaam OR morphine OR analgesic* OR heroin OR narcotic OR heroin OR intravenous OR "dose-response relationship*" OR "drug prescription*")) AND (ti(crim* OR incarcerat* OR convict* OR offend* OR offence* OR offense* OR reincarceration OR reconvict* OR reoffen* OR recidiv* OR rearrest* OR arrest* OR probation* OR parole* OR "community supervis*" OR "technical violat*" OR "drug court*" OR "special* court*" OR "treatment court*") OR ab(crim* OR incarcerat* OR convict* OR offend* OR offence* OR offense* OR reincarceration OR reconvict* OR reoffen* OR recidiv* OR rearrest* OR arrest* OR probation* OR parole* OR "community supervis*" OR "technical violat*" OR "drug court*" OR "special* court*" OR "treatment court*") OR su(crim* OR incarcerat* OR convict* OR offend* OR offence* OR offense* OR reincarceration OR reconvict* OR reoffen* OR recidiv* OR rearrest* OR arrest* OR probation* OR parole* OR "community supervis*" OR "technical violat*" OR "drug court*" OR "special* court*" OR "treatment court*")) AND (ti(overdos*) OR ab(overdos*) OR su(overdos*)) | Date of Publication: 1960-01-01 to 2020-10-31, Language: English, Search mode: Boolean/Phrase |
| Web of Science Core Collection  Web of Science  6/4/21 | TOPIC: (("Medication assisted treat*" OR "Medication assisted therap*" OR drug* OR "therapeutic use*" OR opioid* OR "replacement therap*" OR "substitution therap*" OR pharmacotherap* OR "pharmacological treatment*" OR addict* OR agonist* OR "partial agonist*" OR methadone OR methadose OR dolophine OR buprenorphine OR suboxone OR sublocade OR naltrexone OR depade OR vivitrol OR revia OR "levomethadyl acetate" OR LAAM OR orlaam OR morphine OR analgesic* OR heroin OR narcotic OR heroin OR intravenous OR "dose-response relationship*" OR "drug prescription*") ) AND TOPIC: ((crim* OR incarcerat* OR convict* OR offend* OR offence* OR offense* OR reincarceration OR reconvict* OR reoffen* OR recidiv* OR rearrest* OR arrest* OR probation* OR parole* OR "communitysupervis*" OR "technical violat*" OR "drug court*" OR "special* court*" OR "treatment court*") ) AND TOPIC: (("comparison condition*" OR "comparison group*" OR "control group*" OR "control condition" OR crossover OR effecti* OR efficac* OR evaluat* OR experiment* OR interven* OR match* OR metaanaly* OR pilot* OR placebo* OR program* OR "propensity score*" OR random* OR RCT OR review* OR service* OR therap* OR train* OR treat* OR trial*) ) AND LANGUAGE: (English) | Indexes=SSCI, CPCI-SSH, Timespan=1965-2020, Language: English |
| Web of Science Core Collection  Web of Science  6/8/21 | TOPIC: (("Medication assisted treat*" OR "Medication assisted therap*" OR drug* OR "therapeutic use*" OR opioid* OR "replacement therap*" OR "substitution therap*" OR pharmacotherap* OR "pharmacological treatment*" OR addict* OR agonist* OR "partial agonist*" OR methadone OR methadose OR dolophine OR buprenorphine OR suboxone OR sublocade OR naltrexone OR depade OR vivitrol OR revia OR "levomethadyl acetate" OR LAAM OR orlaam OR morphine OR analgesic* OR heroin OR narcotic OR heroin OR intravenous OR "dose-response relationship*" OR "drug prescription*") ) AND TOPIC: ((crim* OR incarcerat* OR convict* OR offend* OR offence* OR offense* OR reincarceration OR reconvict* OR reoffen* OR recidiv* OR rearrest* OR arrest* OR probation* OR parole* OR "community supervis*" OR "technical violat*" OR "drug court*" OR "special* court*" OR "treatment court*") ) AND TOPIC: ((overdos*) ) AND LANGUAGE: (English) | Indexes=SSCI, CPCI-SSH, Timespan=1965-2020, Language: English |
| Scopus  Elsevier  6/21/21 | ( TITLE-ABS-KEY ( ( "Medication assisted treat*" OR "Medication assisted therap*" OR drug* OR "therapeutic use*" OR opioid* OR "replacement therap*" OR "substitution therap*" OR pharmacotherap* OR "pharmacological treatment*" OR addict* OR agonist* OR "partial agonist*" OR methadone OR methadose OR dolophine OR buprenorphine OR suboxone OR sublocade OR naltrexone OR depade OR vivitrol OR revia OR "levomethadyl acetate" OR laam OR orlaam OR morphine OR analgesic* OR heroin OR narcotic OR heroin OR intravenous OR "dose-response relationship*" OR "drug prescription*" ) ) AND TITLE-ABS-KEY ( ( crim* OR incarcerat* OR convict* OR offend* OR offence* OR offense* OR reincarceration OR reconvict* OR reoffen* OR recidiv* OR rearrest* OR arrest* OR probation* OR parole* OR "community supervis*" OR "technical violat*" OR "drug court*" OR "special* court*" OR "treatment court*" ) ) AND TITLE-ABS-KEY ( ( "comparison condition*" OR "comparison group*" OR "control group*" OR "control condition" OR crossover OR effecti* OR efficac* OR evaluat* OR experiment* OR interven* OR match* OR metaanaly* OR pilot* OR placebo* OR program* OR “propensity AND score*” OR random* OR rct OR review* OR service* OR therap* OR train* OR treat* OR trial* ) ) ) AND PUBYEAR > 1959 AND PUBYEAR < 2021 AND ( LIMIT-TO ( SUBJAREA , "SOCI" ) OR LIMIT-TO ( SUBJAREA , "PSYC" ) OR LIMIT-TO ( SUBJAREA , "MULT" ) ) | Publication Date: 1960-2020, Subject area: Social Sciences, Psychology, Multidisciplinary, Search mode: Boolean/Phrase |
| Scopus  Elsevier  5/13/21 | TITLE-ABS-KEY ( "Medication assisted treat*" OR "Medication assisted therap*" OR drug* OR "therapeutic use*" OR opioid* OR "replacement therap*" OR "substitution therap*" OR pharmacotherap* OR "pharmacological treatment*" OR addict* OR agonist* OR "partial agonist*" OR methadone OR methadose OR dolophine OR buprenorphine OR suboxone OR sublocade OR naltrexone OR depade OR vivitrol OR revia OR "levomethadyl acetate" OR laam OR orlaam OR morphine OR analgesic* OR heroin OR narcotic OR heroin OR intravenous OR "dose-response relationship*" OR "drug prescription*" ) AND TITLE-ABS-KEY ( crim* OR incarcerat* OR convict* OR offend* OR offence* OR offense* OR reincarceration OR reconvict* OR reoffen* OR recidiv* OR rearrest* OR arrest* OR probation* OR parole* OR "community supervis*" OR "technical violat*" OR "drug court*" OR "special* court*" OR "treatment court*" ) AND TITLE-ABS-KEY ( overdos* ) | Publication Date: 1960-2020, Search mode: Boolean/Phrase |
| Cochrane Central Register of Controlled Trials (CENTRAL)  Cochrane  6/10/21 | ( "Medication assisted treat*" OR "Medication assisted therap*" OR drug* OR "therapeutic use*" OR opioid* OR "replacement therap*" OR "substitution therap*" OR pharmacotherap* OR "pharmacological treatment*" OR addict* OR agonist* OR "partial agonist*" OR methadone OR methadose OR dolophine OR buprenorphine OR suboxone OR sublocade OR naltrexone OR depade OR vivitrol OR revia OR "levomethadyl acetate" OR LAAM OR orlaam OR morphine OR analgesic* OR heroin OR narcotic OR heroin OR intravenous OR "dose-response relationship*" OR "drug prescription*" ) in Title Abstract Keyword AND ( crim* OR incarcerat* OR convict* OR offend* OR offence* OR offense* OR reincarceration OR reconvict* OR reoffen* OR recidiv* OR rearrest* OR arrest* OR probation* OR parole* OR "community supervis*" OR "technical violat*" OR "drug court*" OR "special* court*" OR "treatment court*" ) in Title Abstract Keyword AND ( "comparison condition*" OR "comparison group*" OR "control group*" OR "control condition" OR crossover OR effecti* OR efficac* OR evaluat* OR experiment* OR interven* OR match* OR metaanaly* OR pilot* OR placebo* OR program* OR “propensity score*” OR random* OR RCT OR review* OR service* OR therap* OR train* OR treat* OR trial* ) in Title Abstract Keyword - with Publication Year from 1960 to 2020, with Cochrane Library publication date Between Jan 1960 and Oct 2020, in Trials | Publication Date: 1960-2020, Trials only, Search mode: Boolean/Phrase |
| Cochrane Central Register of Controlled Trials (CENTRAL)  Cochrane  6/10/21 | ( "Medication assisted treat*" OR "Medication assisted therap*" OR drug* OR "therapeutic use*" OR opioid* OR "replacement therap*" OR "substitution therap*" OR pharmacotherap* OR "pharmacological treatment*" OR addict* OR agonist* OR "partial agonist*" OR methadone OR methadose OR dolophine OR buprenorphine OR suboxone OR sublocade OR naltrexone OR depade OR vivitrol OR revia OR "levomethadyl acetate" OR LAAM OR orlaam OR morphine OR analgesic* OR heroin OR narcotic OR heroin OR intravenous OR "dose-response relationship*" OR "drug prescription*" ) in Title Abstract Keyword AND ( crim* OR incarcerat* OR convict* OR offend* OR offence* OR offense* OR reincarceration OR reconvict* OR reoffen* OR recidiv* OR rearrest* OR arrest* OR probation* OR parole* OR "community supervis*" OR "technical violat*" OR "drug court*" OR "special* court*" OR "treatment court*" ) in Title Abstract Keyword AND (overdos*) in Title Abstract Keyword - with Publication Year from 1960 to 2020, with Cochrane Library publication date Between Jan 1960 and Oct 2020, in Trials | Publication Date: 1960-2020, Trials only, Search mode: Boolean/Phrase |
| Science.gov  6/10/21 | ( "Medication assisted treat*" OR "Medication assisted therap*" OR drug* OR "therapeutic use*" OR opioid* OR "replacement therap*" OR "substitution therap*" OR pharmacotherap* OR "pharmacological treatment*" OR addict* OR agonist* OR "partial agonist*" OR methadone OR methadose OR dolophine OR buprenorphine OR suboxone OR sublocade OR naltrexone OR depade OR vivitrol OR revia OR "levomethadyl acetate" OR LAAM OR orlaam OR morphine OR analgesic* OR heroin OR narcotic OR heroin OR intravenous OR "dose-response relationship*" OR "drug prescription*" ) AND ( crim* OR incarcerat* OR convict* OR offend* OR offence* OR offense* OR reincarceration OR reconvict* OR reoffen* OR recidiv* OR rearrest* OR arrest* OR probation* OR parole* OR "community supervis*" OR "technical violat*" OR "drug court*" OR "special* court*" OR "treatment court*" ) / From: 1990 / To: 2020 | Publication date: 1990-2020, Sources: Science.gov Websites; General Science; Health & Medicine; Public Access. |
| Science.gov  6/10/21 | ( "Medication assisted treat*" OR "Medication assisted therap*" OR drug* OR "therapeutic use*" OR opioid* OR "replacement therap*" OR "substitution therap*" OR pharmacotherap* OR "pharmacological treatment*" OR addict* OR agonist* OR "partial agonist*" OR methadone OR methadose OR dolophine OR buprenorphine OR suboxone OR sublocade OR naltrexone OR depade OR vivitrol OR revia OR "levomethadyl acetate" OR LAAM OR orlaam OR morphine OR analgesic* OR heroin OR narcotic OR heroin OR intravenous OR "dose-response relationship*" OR "drug prescription*" ) AND ( crim* OR incarcerat* OR convict* OR offend* OR offence* OR offense* OR reincarceration OR reconvict* OR reoffen* OR recidiv* OR rearrest* OR arrest* OR probation* OR parole* OR "community supervis*" OR "technical violat*" OR "drug court*" OR "special* court*" OR "treatment court*" ) AND (overdos*) / From: 1990 / To: 2020 | Publication date: 1990-2020, Sources: Science.gov Websites; General Science; Health & Medicine; Public Access. |
| Crime Solutions  6/23/21 | Hand search of "Drugs & Substance Abuse" topic for any articles involving MAT. |  |

## Appendix 2. All Reports of Included Studies

| **Included Study** | **Other Studies in Family (used to supplement the coding process)** |
| --- | --- |
| Bellin et al. (1999) | None |
| Brinkley-Rubinstein et al. (2018) | The Miriam Hospital (2013); and Rich et al. (2015) |
| Cornish et al. (1997) | None |
| Coviello et al. (2010) | None |
| Dolan et al. (2005) | Dolan et al. (2003) |
| Farabee et al. (2020) | None |
| Farrell-Macdonald et al. (2014) | None |
| Gordon et al. (2018) | Blue et al., (2019); Friends Research Institute, Inc. (2007); Gordon et al. (2013; 2014; 2018); and Kinlock, Gordon, & Schwartz (2009); and Kinlock, Gordon, Schwartz, & Fitzgerald (2010) |
| Haas (2020) | None |
| Hyatt et al. (2021) | None |
| Kinlock et al. (2005) | The MTC Project Team, Kinlock, Battjes, & Schwartz (2002) |
| Kinlock, Gordon, Schwartz, Fitzgerald, & O’Grady (2009) | Gordon, Kinlock, Schwartz, & O'Grady (2008); Kinlock, Gordon, & Schwartz (2007; 2008); Kinlock, Gordon, Schwartz, & O'Grady (2008; 2013); Kinlock, Gordon, Schwartz, O’Grady, Fitzgerald, & Wilson (2007); Kinlock & The Friends Research Institute, Inc. (2006); and Wilson, Kinlock, Gordon, O'Grady, & Schwartz (2012) |
| Lee et al. (2016) | Lee & NYU School of Medicine (2010); Lee et al. (2015a; 2015b; 2015c); Murphy et al. (2017); Friedmann, Wilson, Hoskinson Jr., Poshkus, & Clarke (2018); Soares III et al. (2019); and  O'Brien, Friedmann, Nunes, Lee, Kinlock (2015) |
| Lobmaier, Kunøe, Gossop, Katevoll, & Waal (2010) | Lobmaier, Kunøe, & Waal (2010) |
| MacSwain et al. (2014) | Johnson & Farrell (2008) |
| Magura et al. (2009) | None |
| Marsden et al. (2017) | None |
| McKenzie et al. (2012) | Rich & The Miriam Hospital (2005) |
| Schwartz et al. (2020) | Zarkin et al. (2020); and Kelly et al. (2020) |
| Westerberg et al. (2016) | None |

## Appendix 3. Study Coding Protocol

**SECTION 1: STUDY IDENTIFICATION**

StudyID

DistillerID

Study Citation

Name of funding agent (string) - (e.g., NIH, NIJ)

Other publication about this study/using these data

Publication year (string - YYYY)

Geographic location of study (string - City, State)

Publication type:

   Book=1

   Book chapter=2

   Federal report=3

   State or regional/county report=4

   Agency or technical report =5

   Refereed Journal=6

   Non-refereed journal/grey literature=7

Study participants are:

   Exclusively adults=1

   Exclusively juveniles=2

   Mixed group=3

Study participants are:

   Exclusively males=1

   Exclusively females=2

   Mixed group=3

Notes from section 1 (string)

**SECTION 2: REVIEW INCLUSION ELIGIBILITY CRITERIA**

Content:

Does the study evaluate the effects of MAT on offending outcomes (e.g., official or self-reported offending, arrest, conviction, or incarceration)? (yes=1; no=0)

Does the study report a measure of opioid overdose for a criminal justice-involved population receiving MAT? (yes=1; no=0)

Group assignment is:

   Experimental=1 (groups were randomly assigned to conditions)

   Quasi-experimental=2 (groups were pre-existing or created post hoc)

If quasi-experimental, did they use:

   Matched design=1 (propensity score matching)

   Control variables=2 (to control for any differences between the groups)

Notes from section 2 (string)

**SECTION 3: INTERVENTION/PROGRAM DESCRIPTION**

What happens to the control group? No treatment (yes=1; no=0)

What happens to the control group? Waiting list control (yes=1; no=0)

What happens to the control group? Placebo (yes=1; no=0)

What happens to the control group? Cognitive behavioral therapy (CBT) delivered in an individual or group setting (yes=1; no=0)

What happens to the control group? Non-CBT (e.g., processing) delivered in an individual or group setting

What happens to the control group? Therapeutic community with CBT (yes=1; no=0)

What happens to the control group? Therapeutic community with no CBT (yes=1; no=0)

What happens to the control group? Detoxification/forced withdrawal (yes=1; no=0)

What happens to the control group? Other= (string - describe)

What happens to the control group? Cannot tell (yes=1; no=0)

What happens to the control group? Another type of opioid-specific MAT (yes=1; no=0)

What happens to the control group? Alternative medication not specifically intended for opioid use treatment (e.g, anti-depressant) (yes=1; no=0)

Which medication assisted therapy does the treatment group receive? (string - name the medication)

Experimental group contamination - Does the treatment group receive any intervention outside of the intended ones that the control group does not get? (string - describe only if identified by study authors)

Control group contamination - Does the control group receive any intervention outside of the intended ones that the treatment group does not get? (string - describe only if identified by study authors)

Where does the treatment take place (e.g., jail, prison, community outpatient program, specialty court, etc.)? (string - describe)

Minimum length of treatment (months)

Maximum length of treatment (months)

Mean length of treatment (months)

Fixed (same for all subjects) length of treatment (months)

Level of adherence to MAT:

   Was treatment adherence reported? (yes/no)

What was the adherence (string)?

Medication dosage (string)

Notes from section 3 (string)

**SECTION 4: METHODOLOGICAL RIGOR ASSESSMENT**

Were there statistical differences between groups post-randomization or matching?  (yes/no)

Describe (string)

Variables used to control/match on pre-intervention differences (select all below):

   Age at the beginning of the program (yes=1; no=0)

   Gender (yes=1; no=0)

   Marital status (yes=1; no=0)

   Education, employment and/or economic status (yes=1; no=0)

   Ethnic background or national origin (yes=1; no=0)

   Criminal history:

      Age at first detention

      Prior records (yes=1; no=0)

      Type of offenses (yes=1; no=0)

      Number of drug related charges (yes=1; no=0)

   Addiction history:

      Age at first consumption (yes=1; no=0)

      Type of drugs (yes=1; no=0)

      Previous treatments (yes=1; no=0)

      Other (string)

Additional statistical controls (string):

Reported attrition rate (percent) for treatment group

Reported attrition rate (percent) for control group

Reported attrition rate (percent) for additional treatment group

Did the people who dropped out from the treatment group statistically differ from those who did not (if a significance test reported)? (yes=1; no=0)

Notes from section 4 (string)

**SECTION 5: SIZE AND COMPOSITION OF THE SAMPLE**

Total N in treatment group

Total N in control group

Total N additional treatment group (e.g., if the study compares 3+ groups)

Treatment Group:

Age at the beginning of the program (mean)

Gender: male (%)

Marital status: married (%)

Educational attainment (string - describe indicator and report %)

Employment status: unemployment (%)

Economic status (string - describe indicator and report %)

Race differentiated (yes=1; no=0)

   White (%)

   Black (%)

   Asian (%)

   Hispano (%)

   Others (string, specify with %)

National origin differentiated (yes=1; no=0)

   Non immigrant (%)

   Immigrant (%)

   Criminal history differentiated (yes=1; no=0)

Age at first detention/conviction (mean)

Prior record (%)

Number of prior records (mean)

Arrests (mean/SD)

Convictions (mean/ SD)

Incarcerations (mean/SD)

Type of offense differentiated (yes=1; no=0)

   Drug offenses (%)

   Personnel offenses (sexual) (%)

   Personnel offenses (non-sexual) (%)

   Personnel offenses (unspecified) (%)

   Property offenses (%)

   Traffic offenses (%)

   Other (string, specify with %)

   Non-specified (%)

   All offenses (%)

   Number of drug related charges (mean)

Control Group:

Age at the beginning of the program (mean)

Gender: male (%)

Marital status: married (%)

Educational attainment (string - describe indicator and report %)

Employment status: unemployment (%)

Economic status (string - describe indicator and report %)

Race differentiated (yes=1; no=0)

   White (%)

   Black (%)

   Asian (%)

   Hispano (%)

   Others (string, specify with %)

National origin differentiated (yes=1; no=0)

   Non immigrant (%)

   Immigrant (%)

   Criminal history differentiated (yes=1; no=0)

Age at first detention/conviction (mean)

Prior record (%)

Number of prior records (mean)

Arrests (mean/SD)

Convictions (mean/ SD)

Incarcerations (mean/SD)

Type of offense differentiated (yes=1; no=0)

   Drug offenses (%)

   Personnel offenses (sexual) (%)

   Personnel offenses (non-sexual) (%)

   Personnel offenses (unspecified) (%)

   Property offenses (%)

   Traffic offenses (%)

   Other (string, specify with %)

   Non-specified (%)

   All offenses (%)

   Number of drug related charges (mean)

Additional Treatment Group (if applicable):

Age at the beginning of the program (mean)

Gender: male (%)

Marital status: married (%)

Educational attainment (string - describe indicator and report %)

Employment status: unemployment (%)

Economic status (string - describe indicator and report %)

Race differentiated (yes=1; no=0)

   White (%)

   Black (%)

   Asian (%)

   Hispano (%)

   Others (string, specify with %)

National origin differentiated (yes=1; no=0)

   Non immigrant (%)

   Immigrant (%)

   Criminal history differentiated (yes=1; no=0)

Age at first detention/conviction (mean)

Prior record (%)

Number of prior records (mean)

Arrests (mean/SD)

Convictions (mean/ SD)

Incarcerations (mean/SD)

Type of offense differentiated (yes=1; no=0)

   Drug offenses (%)

   Personnel offenses (sexual) (%)

   Personnel offenses (non-sexual) (%)

   Personnel offenses (unspecified) (%)

   Property offenses (%)

   Traffic offenses (%)

   Other (string, specify with %)

   Non-specified (%)

   All offenses (%)

   Number of drug related charges (mean)

Notes from section 5 (string)

**SECTION 6: OUTCOME INFORMATION**

Re-offending construct represented by this measure:

Rearrest (yes=1; no=0)

Reconviction (yes=1; no=0)

Re-institutionalization/re-incarceration (yes=1; no=0)

Criminal involvement (yes=1; no=0)

Non-specified reoffending (i.e., “general reoffending”) (yes=1; no=0)

Other indicator of criminal involvement (string, specify)

Types of offenses included in re-offending measure:

All offenses (yes=1; no=0)

Drug offenses (yes=1; no=0)

Person offenses, sexual (yes=1; no=0)

Person offenses, non-sexual (yes=1; no=0)

Person offenses, unspecified (yes=1; no=0)

Property offenses (yes=1; no=0)

Traffic offenses (yes=1; no=0)

Other (string, specify)

Overdose construct represented by this measure:

Fatal overdose (yes=1; no=0)

Nonfatal overdose (yes=1; no=0)

Non-specified overdose (yes=1; no=0)

Type of measurement scale for reoffending outcome(s):

Dichotomy=1

Trichotomy=2

Four or more discrete ordinal categories=3

Continuous measure=4

Survival measure=5

Type of measurement scale for overdose outcome(s):

Dichotomy=1

Trichotomy=2

Four or more discrete ordinal categories=3

Continuous measure=4

Survival measure=5

Data source(s) for CJ outcomes:

Self-report (yes=1; no=0)

Official records (e.g., court, police, administrative) (yes=1; no=0)

Data source(s) for overdose outcomes:

Self-report (yes=1; no=0)

Official records (e.g., court, police, administrative) (yes=1; no=0)

Length of follow up period:

Minimum (months)

Maximum (months)

Mean (months)

Fixed (the same for all subjects) (months)

Notes from section 6 (string)

**SECTION 7: EFFECT SIZE INFORMATION**

Does this study report effect sizes at different time points? (yes=1; no=0).

How many time point effect sizes are reported?

Rearrest effect size reported (yes=1; no=0)

Reconviction effect size reported (yes=1; no=0)

Reincarceration effect size reported (yes=1; no=0)

General offending effect size reported (yes=1; no=0)

Fatal overdose effect size reported (yes=1; no=0)

Nonfatal overdose effect size reported (yes=1; no=0)

Non-specific overdose effect size reported (yes=1; no=0)

Measurement type:

Failure proportion=1

Mean frequency=2

Survival analysis=3

Notes from section 7 (string)

**SECTION 8: EFFECT SIZE DATA**

Sample size:

Treatment group

Control group

Additional treatment group

Means and standard deviations (report only if indicator was "mean frequency")

Treatment group mean rearrests

Treatment group standard deviation rearrests

Control group mean rearrests

Control group standard deviation rearrests

Additional treatment group mean rearrests

Additional treatment group standard deviation rearrests

Treatment group mean reconviction

Treatment group standard deviation reconviction

Control group mean reconviction

Control group standard deviation reconviction

Additional treatment group mean reconviction

Additional treatment group standard deviation reconviction

Treatment group mean reincarceration

Treatment group standard deviation reincarceration

Control group mean reincarceration

Control group standard deviation reincarceration

Additional treatment group mean reincarceration

Additional treatment group standard deviation reincarceration

Treatment group mean general reoffending

Treatment group standard deviation general reoffending

Control group mean general reoffending

Control group standard deviation general reoffending

Additional treatment group mean general reoffending

Additional treatment group standard deviation general reoffending

Treatment group mean nonfatal overdose

Treatment group standard deviation nonfatal overdose

Control group mean nonfatal overdose

Control group standard deviation nonfatal overdose

Additional treatment group mean nonfatal overdose

Additional treatment group standard deviation nonfatal overdose

Treatment group mean fatal overdose

Treatment group standard deviation fatal overdose

Control group mean fatal overdose

Control group standard deviation fatal overdose

Additional treatment group mean fatal overdose

Additional treatment group standard deviation fatal overdose

Treatment group mean non-specified overdose

Treatment group standard deviation non-specified overdose

Control group mean non-specified overdose

Control group standard deviation non-specified overdose

Additional treatment group mean non-specified overdose

Additional treatment group standard deviation non-specified overdose

Failure proportion (only report if indicator was "failure proportion")

Proportion of treatment group that was rearrested

Proportion of control group that was rearrested

Proportion of additional treatment group that was rearrested

Proportion of treatment group that was reconvicted

Proportion of control group that was reconvicted

Proportion of additional treatment group that was reconvicted

Proportion of treatment group that was reincarcerated

Proportion of control group that was reincarcerated

Proportion of additional treatment group that was reincarcerated

Proportion of treatment group that re-offended (general)

Proportion of control group that re-offended (general)

Proportion of additional treatment group that re-offended (general)

Proportion of treatment group that had nonfatal overdose

Proportion of control group that had nonfatal overdose

Proportion of additional treatment group that had nonfatal overdose

Proportion of treatment group that had fatal overdose

Proportion of control group that had fatal overdose

Proportion of additional treatment group that had fatal overdose

Proportion of treatment group that had non-specified overdose

Proportion of control group that had non-specified overdose

Proportion of additional treatment group that had non-specified overdose

Survival (report only if indicator was "survival analysis")

Mean survival time to rearrest for the treatment group

Mean survival time to rearrest for the control group

Mean survival time to rearrest for the additional treatment group

Mean survival time to reconviction for the treatment group

Mean survival time to reconviction for the control group

Mean survival time to reconviction for the additional treatment group

Mean survival time to reincarceration for the treatment group

Mean survival time to reincarceration for the control group

Mean survival time to reincarceration for the additional treatment group

Mean survival time to reoffending for the treatment group

Mean survival time to reoffending for the control group

Mean survival time to reoffending for the additional treatment group

Mean survival time to nonfatal overdose for the treatment group

Mean survival time to nonfatal overdose for the control group

Mean survival time to nonfatal overdose for the additional treatment group

Mean survival time to fatal overdose for the treatment group

Mean survival time to fatal overdose for the control group

Mean survival time to fatal overdose for the additional treatment group

Mean survival time to non-specified overdose for the treatment group

Mean survival time to non-specified overdose for the control group

Mean survival time to non-specific overdose for the additional treatment group

Odds ratio (logistic regression) (record in Notes if hazard ratio reported)

Calculated for the treatment group - rearrest

Calculated for the control group - rearrest

Calculated for additional treatment group - rearrest

Calculated for the treatment group - reconviction

Calculated for the control group - reconviction

Calculated for additional treatment group - reconviction

Calculated for the treatment group - reincarceration

Calculated for the control group - reincarceration

Calculated for additional treatment group - reincarceration

Calculated for the treatment group - general reoffending

Calculated for the control group - general reoffending

Calculated for additional treatment group - general reoffending

Calculated for the treatment group - nonfatal overdose

Calculated for the control group - nonfatal overdose

Calculated for additional treatment group - nonfatal overdose

Calculated for the treatment group - fatal overdose

Calculated for the control group - fatal overdose

Calculated for additional treatment group - fatal overdose

Calculated for the treatment group - non-specified overdose

Calculated for the control group - non-specified overdose

Calculated for additional treatment group - non-specified overdose

Which group does the raw effect favor in terms of rearrest (ignoring statistical significance)?

   Treatment group=1

   Control group=2

   Additional treatment group=3

   Neither (ES equals zero)=4

   Cannot tell (NOTE: ES cannot be used if this option is selected)=5

Is this difference reported as statistically significant?

   No=0

   Yes=1

   Not tested=2

   Cannot tell=3

Type of statistical test used:

   T-test=1

   F-test=2

   Chi-square=3

   Regression analysis=4

   Other (string, specify)

   No analysis (N/A)=5

   Cannot tell=6

Which group does the raw effect favor in terms of reconviction (ignoring statistical significance)?

   Treatment group=1

   Control group=2

   Additional treatment group=3

   Neither (ES equals zero)=4

   Cannot tell (NOTE: ES cannot be used if this option is selected)=5

Is this difference reported as statistically significant?

   No=0

   Yes=1

   Not tested=2

   Cannot tell=3

Type of statistical test used:

   T-test=1

   F-test=2

   Chi-square=3

   Regression analysis=4

   Other (string, specify)

   No analysis (N/A)=5

   Cannot tell=6

Which group does the raw effect favor in terms of reincarceration (ignoring statistical significance)?

   Treatment group=1

   Control group=2

   Additional treatment group=3

   Neither (ES equals zero)=4

   Cannot tell (NOTE: ES cannot be used if this option is selected)=5

Is this difference reported as statistically significant?

   No=0

   Yes=1

   Not tested=2

   Cannot tell=3

Type of statistical test used:

   T-test=1

   F-test=2

   Chi-square=3

   Regression analysis=4

   Other (string, specify)

   No analysis (N/A)=5

   Cannot tell=6

Which group does the raw effect favor in terms of general reoffending (ignoring statistical significance)?

   Treatment group=1

   Control group=2

   Additional treatment group=3

   Neither (ES equals zero)=4

   Cannot tell (NOTE: ES cannot be used if this option is selected)=5

Is this difference reported as statistically significant?

   No=0

   Yes=1

   Not tested=2

   Cannot tell=3

Type of statistical test used:

   T-test=1

   F-test=2

   Chi-square=3

   Regression analysis=4

   Other (string, specify)

   No analysis (N/A)=5

   Cannot tell=6

Which group does the raw effect favor in terms of nonfatal overdose (ignoring statistical significance)?

   Treatment group=1

   Control group=2

   Additional treatment group=3

   Neither (ES equals zero)=4

   Cannot tell (NOTE: ES cannot be used if this option is selected)=5

Is this difference reported as statistically significant?

   No=0

   Yes=1

   Not tested=2

   Cannot tell=3

Type of statistical test used:

   T-test=1

   F-test=2

   Chi-square=3

   Regression analysis=4

   Other (string, specify)

   No analysis (N/A)=5

   Cannot tell=6

Which group does the raw effect favor in terms of fatal overdose (ignoring statistical significance)?

   Treatment group=1

   Control group=2

   Additional treatment group=3

   Neither (ES equals zero)=4

   Cannot tell (NOTE: ES cannot be used if this option is selected)=5

Is this difference reported as statistically significant?

   No=0

   Yes=1

   Not tested=2

   Cannot tell=3

Type of statistical test used:

   T-test=1

   F-test=2

   Chi-square=3

   Regression analysis=4

   Other (string, specify)

   No analysis (N/A)=5

   Cannot tell=6

Which group does the raw effect favor in terms of non-specified overdose (ignoring statistical significance)?

   Treatment group=1

   Control group=2

   Additional treatment group=3

   Neither (ES equals zero)=4

   Cannot tell (NOTE: ES cannot be used if this option is selected)=5

Is this difference reported as statistically significant?

   No=0

   Yes=1

   Not tested=2

   Cannot tell=3

Type of statistical test:

   T-test=1

   F-test=2

   Chi-square=3

   Regression analysis=4

   Other (string, specify)

   No analysis (N/A)=5

   Cannot tell=6

Notes from section 8 (string)

Notes/concerns/questions about the study in general (string)

## Appendix 4. Experimental Studies Risk of Bias (RoB) Assessments (*n* =14)

| Study | Agreement across Domains | Overall Risk of Bias Rating | Rationale for Rating |
| --- | --- | --- | --- |
| Brinkley-Rubinstein et al. (2018) | 100% | Some (2) | 45 participants in the control condition (detoxification/forced withdrawal) were released before completing the withdrawal regimen, and 3 participants in the treatment condition withdrew before release. There was 20% attrition for the 12-month outcome interview. Outcome administration data were described as “incomplete”. Nevertheless, no significant baseline differences between those who did and did not complete a follow up. Re-arrest outcome is based solely on self-report. // At least one domain was rated Some Risk. |
| Cornish  et al. (1997) | 100% | Some (2) | No information about concealment of allocation sequence; Based upon compliance with the protocol as the definition of retention, 52% of the experimental group was adherent during the entire period and 33% of the control group was adherent. Those not adherent for two consecutive weeks or more were dropped from the study. // At least one domain was rated as Some Risk. |
| Coviello  et al. (2010) | 100% | High (3) | No information about concealment of allocation sequence; Low treatment retention and high drop out rates observed; 55% of the treatment group and 58% of the comparison group completed the 6-month follow up, so there is a lot of missing outcome data for both groups. // At least one domain was rated as High Risk. |
| Dolan  et al. (2005) | 100% | Some (2) | By the time of follow up, 97% of participants across both groups had received methadone maintenance therapy at some point during the follow up; Authors recognize that study began as a randomized trial but the long follow up opened the door for confounding. However, this was addressed with control for several time-varying covariates and person-time analyses looking at periods of exposed and unexposed periods of MAT. // At least one domain was rated as Some Risk. |
| Farabee  et al. (2020) | 100% | Some (2) | Low treatment adherence in both conditions with MAT. The treatment group received a M of 2.2 out of 7 injections and attended 3.5 of 10 medication management sessions and 5 treatment group participants never received any injections. MAT + patient navigation had 3 of 7 injections and attended 4 of 10 medication management visits. Some attrition at follow up, but mitigated with official data on outcomes. // At least one domain was rated as Some Risk. |
| Gordon  et al. (2018) | 100% | Some (2) | Some buprenorphine participants took medication out of their mouths and were terminated from treatment. As a result, the study switched from tablets to fast-dissolving strips. A closure of a female prison resulted in inability to enroll previously recruited female participants into MAT treatment for much of the study period; Official records missing for 12 participants, evenly distributed across treatment and comparison groups. // At least one domain was rated Some Risk. |
| Hyatt  et al. (2021) | 100% | Some (2) | Adherence to treatment and control conditions not tracked after release because the study was not designed as an ongoing intervention. Therefore, most participants received dosage post release that was clinically indicated and not necessarily planned. // At least one domain was rated as Some Risk. |
| Kinlock  et al. (2005) | 100% | High (3) | No information on randomization procedures or concealment of allocation sequence; Substantial treatment attrition and study attrition (i.e., missing follow up data). // At least one domain was rated as High Risk. |
| Kinlock  et al. (2009) | 80% | Some (2) | Comparison groups successfully initiated treatment at significantly lower rates than the MAT group. Statistically significant larger drop out from counseling only than MAT or counseling and MAT-transfer groups. // At least one domain was rated Some Risk. |
| Lee  et al. (2016) | 80% | Some (2) | Some deviation from the treatment protocol after release for the MAT group; Over one-third of participants in TAU group pursued agonist MAT after release. // At least one domain was rated Some Risk. |
| Lobmaier  et al. (2010) | 100% | High (3) | Some of the randomized participants (regardless of condition) did not even begin treatment; aftercare was low in both groups; 70% of naltrexone and 24% of methadone participants were still receiving MAT at 6 months. This was statistically significant. Outcome data based on self-report/timeline follow back). // At least one domain was rated as High Risk. |
| Magura  et al. (2009) | 100% | Some (2) | Some of the randomized participants (regardless of condition) did not even begin treatment; Buprenorphine group had significantly higher retention than methadone group; Many in the methadone group received “sub-optimal doses”; Overall study retention for follow up interview was only 70%, but groups did not differ. // Several domains were rated as Some Risk. |
| McKenzie  et al. (2012) | 100% | High (3) | No information on concealment of allocation; Significant cross-over of two comparator arms. Significant treatment attrition in all groups; Attrition from the study also notable, but not different between groups (~30%). // Several domains were rated as Some Risk. |
| Schwartz  et al. (2020) | 80% | Some (2) | Significant treatment attrition and study attrition for both groups. Outcomes were assessed with self-report only, which is subject to recall bias and positive impression management. Statistical analyses done to handle much of these issues but not all. // At least one domain was rated Some Risk. |

## Appendix 5. Quasi-Experimental Studies ROBINS-I Assessment (*n* = 6)

| Study | Agreement across Domains | Overall Risk of Bias | Rationale for Rating |
| --- | --- | --- | --- |
| Bellin  et al. (1999) | 86% | Serious (3) | Some but not all group differences are controlled for in the analyses; Researchers did not track adherence/deviation from medication over time; Results were reported with more detail for the high vs. low dose comparisons than for either vs. forced withdrawal comparator. // At least one domain was rated Serious Risk. |
| Farrell  et al. (2014) | 100% | No Information (5) | One statistically significant difference between groups that was not controlled for in the analyses; No information reported on treatment adherence or control group contamination (e.g., receipt of other services) over the 2-year follow up. // All domains were rated as Low Risk except for deviation from the intervention domain, which was rated as missing. |
| Haas  (2020) | 100% | Moderate (2) | Volunteer sample; Did not examine many group differences between two jail sites from where participants were recruited, but subgroup analysis yielded consistency in findings. Treatment and comparison group differed on race; Examined access to services in the community (vs. dosage or adherence); Used official records for outcomes; no missing data. // Only one domain (selection) was rated as Moderate Risk. |
| MacSwain  et al. (2014) | 100% | No Information (5) | No information reported on treatment adherence or control group contamination (e.g., receipt of other services) over the 2-year follow up // All domains were rated as Low Risk except for deviation from the intervention domain, which was rated as missing. |
| Marsden  et al. (2017) | 100% | Moderate (2) | Possible concerns in grouping people with no methadone exposure and people with exposure not meeting a certain threshold level, given that this could be individuals both decreasing (as assumed) and increasing dosage over time. // At least one domain was rated Moderate Risk. |
| Westerberg  et al. (2016) | 100% | Serious (3) | Likely important differences between treatment groups not due to the intervention, including higher motivation and more treatment continuity for the treatment group; Any assessment of adherence or deviation from treatment was assessed with a similar sample but not the same included in the current study. // At least one domain was rated as  Serious Risk. |

## Appendix 6. Key Excluded Studies

| Study Citation  [Other studies in family] | Study Title |
| --- | --- |
| Group 1: Studies that did not include any or enough statistical controls for baseline differences between groups, or the groups examined for the outcome were not the same as the groups initially being compared. | |
| Clark et al. (2014) | Methadone Maintenance Treatment May Improve Completion Rates and Delay Opioid Relapse for Opioid Dependent Individuals Under Community Corrections Supervision |
| Cornish et al. (2012) | Six-month Depot Naltrexone Treatment Reduces Relapse in Parolees Formerly Addicted to Opioids |
| Doernberg (2019) | Impact of Two Pilot Methadone Treatment Programs in Connecticut Correctional Facilities on Post-Release Outcomes |
| Larney et al. (2012) | Effect of Prison‐based Opioid Substitution Treatment and Post‐release Retention in Treatment on Risk of Re‐incarceration |
| Huang et al. (2011) | Mortality Among a Cohort of Drug Users After their Release from Prison: An Evaluation of the Effectiveness of a Harm Reduction Program in Taiwan |
| Hume & Gorta (1989) | The Effects of the NSW Prison Methadone Program on Criminal Recidivism and Retention in Methadone Treatment |
| Johnson et al. (2001) | Institutional Methadone Treatment: Impact on Release Outcomes and Institutional Behaviour |
| Magura et al. (1993) | The Effectiveness of In-jail Methadone Maintenance |
| Moore et al. (2018) | Feasibility and Effectiveness of Continuing Methadone Maintenance Treatment During Incarceration Compared with Forced Withdrawal |
| Group 2: Studies at high risk of bias as determined by the ROBINS-I tool. | |
| Zaller et al. (2013)^[[1]](#footnote-2)^ | [Initiation of Buprenorphine During Incarceration and Retention in Treatment Upon Release](https://www.sciencedirect.com/science/article/pii/S0740547213000573) |
| Group 3: Studies with data either published outside of search window or the study is ongoing (with no available outcomes) as verified with the corresponding author. | |
| Gordon et al. (2021)  [Gordon & Mitchell (2020)] | A Clinical Protocol of a Comparative Effectiveness Trial of Extended-release Naltrexone Versus Extended-release Buprenorphine with Individuals Leaving Jail |
| Gordon et al., (2017) | Extended-release Naltrexone for Pre-release Prisoners: A Randomized Trial of Medical Mobile Treatment |
| McDonald et al. (2016)  [Velasquez et al. (2019)] | Extended-release Naltrexone Opioid Treatment at Jail Reentry (XOR) |
| Schwartz et al. (2021)  [Mitchell et al. (2020; 2021); Schwartz et al. (2016); Friends Research Institute, Inc. (2015)] | Randomized Trial of Methadone Treatment of Arrestees: 24-month Post-release Outcomes |
| Di Paola et al. (2014)  [Springer et al. (2015; 2018); Vagenas et al. (2014)] | Design and Methods of a Double-blind Randomized Placebo-controlled Trial of Extended-release Naltrexone for HIV-infected, Opioid-dependent Prisoners and Jail Detainees who are Transitioning to the Community |
| Woody et al. (2021)  [Woody (2015)] | Extended-release Injectable Naltrexone Before vs. After Release: A Randomized Trial of Opioid Addicted Persons Who are in Prison |
| Friends Research Institute, Inc. (2018)  [Gordon et al. (2019)] | Buprenorphine for Probationers and Parolees: Bridging the Gap into Treatment |

| Appendix 7. Revised Cochrane Risk-of-bias Tool for Randomized Trials (RoB 2) **Study details**   \| **Reference** \|  \| \| --- \| --- \|   **Study design**   \| X \| Individually-randomized parallel-group trial \| \| --- \| --- \| \| □ \| Cluster-randomized parallel-group trial \| \| □ \| Individually randomized cross-over (or other matched) trial \|   **For the purposes of this assessment, the interventions being compared are defined as**   \| Experimental: \|  \| Comparator: \|  \| \| --- \| --- \| --- \| --- \|  \| **Specify which outcome is being assessed for risk of bias** \|  \| \| --- \| --- \|  \| **Specify the numerical result being assessed.** In case of multiple alternative analyses being presented, specify the numeric result (e.g. RR = 1.52 (95% CI 0.83 to 2.77) and/or a reference (e.g. to a table, figure or paragraph) that uniquely defines the result being assessed. \|  \| \| --- \| --- \|   **Is the review team’s aim for this result…?**   \| □ \| to assess the effect of *assignment to intervention* (the ‘intention-to-treat’ effect) \| \| --- \| --- \| \| □ \| to assess the effect of *adhering to intervention* (the ‘per-protocol’ effect) \|   **If the aim is to assess the effect of *adhering to intervention***, select the deviations from intended intervention that should be addressed (at least one must be checked):  □ occurrence of non-protocol interventions  □ failures in implementing the intervention that could have affected the outcome  □ non-adherence to their assigned intervention by trial participants  **Which of the following sources were obtained to help inform the risk-of-bias assessment? (tick as many as apply)**  □ Journal article(s) with results of the trial  □ Trial protocol  □ Statistical analysis plan (SAP)  □ Non-commercial trial registry record (e.g. ClinicalTrials.gov record)  □ Company-owned trial registry record (e.g. GSK Clinical Study Register record)  □ “Grey literature” (e.g. unpublished thesis)  □ Conference abstract(s) about the trial  □ Regulatory document (e.g. Clinical Study Report, Drug Approval Package)  □ Research ethics application  □ Grant database summary (e.g. NIH RePORTER or Research Councils UK Gateway to Research)  □ Personal communication with trialist  □ Personal communication with the sponsor |
| --- | --- | --- | --- | --- | --- | --- | --- | --- | --- | --- | --- | --- | --- | --- | --- | --- | --- | --- | --- | --- |

**Risk of bias assessment**

Responses underlined in green are potential markers for low risk of bias, and responses in red are potential markers for a risk of bias. Where questions relate only to sign posts to other questions, no formatting is used.

| Domain 1: Risk of bias arising from the randomization process | | |
| --- | --- | --- |
| **Signalling questions** | **Comments** | **Response options** |
| **1.1 Was the allocation sequence random?** |  | Y / PY / PN / N / NI |
| **1.2 Was the allocation sequence concealed until participants were enrolled and assigned to interventions?** |  | Y / PY / PN / N / NI |
| **1.3 Did baseline differences between intervention groups suggest a problem with the randomization process?** |  | Y / PY / PN / N / NI |
| **Risk-of-bias judgement** |  | Low / High / Some concerns |
| Optional: What is the predicted direction of bias arising from the randomization process? |  | NA / Favours experimental / Favours comparator / Towards null /Away from null / Unpredictable |
| Domain 2: Risk of bias due to deviations from the intended interventions (*effect of* ***assignment*** *to intervention*) | | |
| **Signalling questions** | **Comments** | **Response options** |
| **2.1. Were participants aware of their assigned intervention during the trial?** |  | Y / PY / PN / N / NI |
| **2.2. Were carers and people delivering the interventions aware of participants' assigned intervention during the trial?** |  | Y / PY / PN / N / NI |
| **2.3. If Y/PY/NI to 2.1 or 2.2: Were there deviations from the intended intervention that arose because of the trial context?** |  | NA / Y / PY / PN / N / NI |
| **2.4 If Y/PY to 2.3: Were these deviations likely to have affected the outcome?** |  | NA / Y / PY / PN / N / NI |
| **2.5. If Y/PY/NI to 2.4: Were these deviations from intended intervention balanced between groups?** |  | NA / Y / PY / PN / N / NI |
| **2.6 Was an appropriate analysis used to estimate the effect of assignment to intervention?** |  | Y / PY / PN / N / NI |
| **2.7 If N/PN/NI to 2.6: Was there potential for a substantial impact (on the result) of the failure to analyse participants in the group to which they were randomized?** |  | NA / Y / PY / PN / N / NI |
| **Risk-of-bias judgement** |  | Low / High / Some concerns |
| Optional: What is the predicted direction of bias due to deviations from intended interventions? |  | NA / Favours experimental / Favours comparator / Towards null /Away from null / Unpredictable |
| Domain 2: Risk of bias due to deviations from the intended interventions (*effect of* ***adhering*** *to intervention*) | | |
| **Signalling questions** | **Comments** | **Response options** |
| **2.1. Were participants aware of their assigned intervention during the trial?** |  | Y / PY / PN / N / NI |
| **2.2. Were carers and people delivering the interventions aware of participants' assigned intervention during the trial?** |  | Y / PY / PN / N / NI |
| **2.3. [If applicable:] If Y/PY/NI to 2.1 or 2.2: Were important non-protocol interventions balanced across intervention groups?** |  | NA / Y / PY / PN / N / NI |
| **2.4. [If applicable:] Were there failures in implementing the intervention that could have affected the outcome?** |  | NA / Y / PY / PN / N / NI |
| **2.5. [If applicable:] Was there non-adherence to the assigned intervention regimen that could have affected participants’ outcomes?** |  | NA / Y / PY / PN / N / NI |
| **2.6. If N/PN/NI to 2.3, or Y/PY/NI to 2.4 or 2.5: Was an appropriate analysis used to estimate the effect of adhering to the intervention?** |  | NA / Y / PY / PN / N / NI |
| **Risk-of-bias judgement** |  | Low / High / Some concerns |
| Optional: What is the predicted direction of bias due to deviations from intended interventions? |  | NA / Favours experimental / Favours comparator / Towards null /Away from null / Unpredictable |
| Domain 3: Missing outcome data | | |
| **Signalling questions** | **Comments** | **Response options** |
| **3.1 Were data for this outcome available for all, or nearly all, participants randomized?** |  | Y / PY / PN / N / NI |
| **3.2 If N/PN/NI to 3.1: Is there evidence that the result was not biased by missing outcome data?** |  | NA / Y / PY / PN / N |
| **3.3 If N/PN to 3.2: Could missingness in the outcome depend on its true value?** |  | NA / Y / PY / PN / N / NI |
| **3.4 If Y/PY/NI to 3.3: Is it likely that missingness in the outcome depended on its true value?** |  | NA / Y / PY / PN / N / NI |
| **Risk-of-bias judgement** |  | Low / High / Some concerns |
| Optional: What is the predicted direction of bias due to missing outcome data? |  | NA / Favours experimental / Favours comparator / Towards null /Away from null / Unpredictable |
| Domain 4: Risk of bias in measurement of the outcome | | |
| **Signalling questions** | **Comments** | **Response options** |
| **4.1 Was the method of measuring the outcome inappropriate?** |  | Y / PY / PN / N / NI |
| **4.2 Could measurement or ascertainment of the outcome have differed between intervention groups?** |  | Y / PY / PN / N / NI |
| **4.3 If N/PN/NI to 4.1 and 4.2: Were outcome assessors aware of the intervention received by study participants?** |  | NA / Y / PY / PN / N / NI |
| **4.4 If Y/PY/NI to 4.3: Could assessment of the outcome have been influenced by knowledge of intervention received?** |  | NA / Y / PY / PN / N / NI |
| **4.5 If Y/PY/NI to 4.4:** **Is it likely that assessment of the outcome was influenced by knowledge of intervention received?** |  | NA / Y / PY / PN / N / NI |
| **Risk-of-bias judgement** |  | Low / High / Some concerns |
| Optional: What is the predicted direction of bias in measurement of the outcome? |  | NA / Favours experimental / Favours comparator / Towards null /Away from null / Unpredictable |
| Domain 5: Risk of bias in selection of the reported result | | |
| **Signalling questions** | **Comments** | **Response options** |
| **5.1 Were the data that produced this result analysed in accordance with a pre-specified analysis plan that was finalized before unblinded outcome data were available for analysis?** |  | Y / PY / PN / N / NI |
| **Is the numerical result being assessed likely to have been selected, on the basis of the results, from...** |  |  |
| **5.2. ... multiple eligible outcome measurements (e.g. scales, definitions, time points) within the outcome domain?** |  | Y / PY / PN / N / NI |
| **5.3 ... multiple eligible analyses of the data?** |  | Y / PY / PN / N / NI |
| **Risk-of-bias judgement** |  | Low / High / Some concerns |
| Optional: What is the predicted direction of bias due to selection of the reported result? |  | NA / Favours experimental / Favours comparator / Towards null /Away from null / Unpredictable |
| Overall risk of bias | | |
| **Risk-of-bias judgement** |  | Low / High / Some concerns |
| Optional: What is the overall predicted direction of bias for this outcome? |  | NA / Favours experimental / Favours comparator / Towards null /Away from null / Unpredictable |

Edited by Julian PT Higgins, Jelena Savović, Matthew J Page, Jonathan AC Sterne
on behalf of the RoB2 Development Group

**Version of 22 August 2019**

The development of the RoB 2 tool was supported by the MRC Network of Hubs for Trials Methodology Research (MR/L004933/2- N61), with the support of the host MRC ConDuCT-II Hub (Collaboration and innovation for Difficult and Complex randomised controlled Trials In Invasive procedures - MR/K025643/1), by MRC research grant MR/M025209/1, and by a grant from The Cochrane Collaboration.


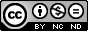


This work is licensed under a [Creative Commons Attribution-NonCommercial-NoDerivatives 4.0 International License](http://creativecommons.org/licenses/by-nc-nd/4.0/).

## Appendix 8. The Risk Of Bias In Non-randomized Studies – of Interventions (ROBINS-I) Assessment Tool

**ROBINS-I tool (Stage I): At protocol stage**

**Specify the review question**

| Participants |  |
| --- | --- |
| Experimental intervention |  |
| Comparator |  |
| Outcomes |  |

**List the confounding domains relevant to all or most studies**

|  |
| --- |

**List co-interventions that could be different between intervention groups and that could impact on outcomes**

|  |
| --- |

**ROBINS-I tool (Stage II): For each study**

**Specify a target randomized trial specific to the study**

| Design | Individually randomized / Cluster randomized / Matched (e.g. cross-over) |
| --- | --- |
| Participants |  |
| Experimental intervention |  |
| Comparator |  |

**Is your aim for this study…?**

| □ | to assess the effect of *assignment to* intervention |
| --- | --- |
| □ | to assess the effect of *starting and adhering to* intervention |

**Specify the outcome**

**Specify which outcome is being assessed for risk of bias (typically from among those earmarked for the Summary of Findings table). Specify whether this is a proposed benefit or harm of intervention.**

|  |
| --- |

**Specify the numerical result being assessed**

**In case of multiple alternative analyses being presented, specify the numeric result (e.g. RR = 1.52 (95% CI 0.83 to 2.77) and/or a reference (e.g. to a table, figure or paragraph) that uniquely defines the result being assessed.**

|  |
| --- |

**Preliminary consideration of confounders**

Complete a row for each important confounding domain (i) listed in the review protocol; and (ii) relevant to the setting of this particular study, or which the study authors identified as potentially important.

#### “Important” confounding domains are those for which, in the context of this study, adjustment is expected to lead to a clinically important change in the estimated effect of the intervention. “Validity” refers to whether the confounding variable or variables fully measure the domain, while “reliability” refers to the precision of the measurement (more measurement error means less reliability).

| **(i) Confounding domains listed in the review protocol** | | | | |
| --- | --- | --- | --- | --- |
| Confounding domain | Measured variable(s) | Is there evidence that controlling for this variable was unnecessary?* | Is the confounding domain measured validly and reliably by this variable (or these variables)? | OPTIONAL: Is failure to adjust for this variable (alone) expected to favour the experimental intervention or the comparator? |
|  |  |  | Yes / No / No information | Favour experimental / Favour comparator / No information |
| **(ii) Additional confounding domains relevant to the setting of this particular study, or which the study authors identified as important** | | | | |
| Confounding domain | Measured variable(s) | Is there evidence that controlling for this variable was unnecessary?* | Is the confounding domain measured validly and reliably by this variable (or these variables)? | OPTIONAL: Is failure to adjust for this variable (alone) expected to favour the experimental intervention or the comparator? |
|  |  |  | Yes / No / No information | Favour experimental / Favour comparator / No information |

* In the context of a particular study, variables can be demonstrated not to be confounders and so not included in the analysis: (a) if they are not predictive of the outcome; (b) if they are not predictive of intervention; or (c) because adjustment makes no or minimal difference to the estimated effect of the primary parameter. Note that “no statistically significant association” is not the same as “not predictive”.

**Preliminary consideration of co-interventions**

Complete a row for each important co-intervention (i) listed in the review protocol; and (ii) relevant to the setting of this particular study, or which the study authors identified as important.

#### “Important” co-interventions are those for which, in the context of this study, adjustment is expected to lead to a clinically important change in the estimated effect of the intervention.

| **(i) Co-interventions listed in the review protocol** | | |
| --- | --- | --- |
| Co-intervention | Is there evidence that controlling for this co-intervention was unnecessary (e.g. because it was not administered)? | Is presence of this co-intervention likely to favour outcomes in the experimental intervention or the comparator |
|  |  | Favour experimental / Favour comparator / No information |
| **(ii) Additional co-interventions relevant to the setting of this particular study, or which the study authors identified as important** | | |
| Co-intervention | Is there evidence that controlling for this co-intervention was unnecessary (e.g. because it was not administered)? | Is presence of this co-intervention likely to favour outcomes in the experimental intervention or the comparator |
|  |  | Favour experimental / Favour comparator / No information |

**Risk of bias assessment**

Responses underlined in green are potential markers for low risk of bias, and responses in red are potential markers for a risk of bias. Where questions relate only to sign posts to other questions, no formatting is used.

| **Signalling questions** | **Description** | **Response options** |
| --- | --- | --- |
| **Bias due to confounding** | | |
| 1.1 Is there potential for confounding of the effect of intervention in this study?  **If N/PN to 1.1:** the study can be considered to be at low risk of bias due to confounding and no further signalling questions need be considered |  | Y / PY / PN / N |
| **If Y/PY to 1.1**: determine whether there is a need to assess time-varying confounding: |  |  |
| 1.2. Was the analysis based on splitting participants’ follow up time according to intervention received?  **If N/PN**, answer questions relating to baseline confounding (1.4 to 1.6)  **If Y/PY**, go to question 1.3. |  | NA / Y / PY / PN / N / NI |
| 1.3. Were intervention discontinuations or switches likely to be related to factors that are prognostic for the outcome?  **If N/PN**, answer questions relating to baseline confounding (1.4 to 1.6)  **If Y/PY**, answer questions relating to both baseline and time-varying confounding (1.7 and 1.8) |  | NA / Y / PY / PN / N / NI |

| **Questions relating to baseline confounding only** | | |
| --- | --- | --- |
| 1.4. Did the authors use an appropriate analysis method that controlled for all the important confounding domains? |  | NA / Y / PY / PN / N / NI |
| 1.5. **If Y/PY to 1.4**: Were confounding domains that were controlled for measured validly and reliably by the variables available in this study? |  | NA / Y / PY / PN / N / NI |
| 1.6. Did the authors control for any post-intervention variables that could have been affected by the intervention? |  | NA / Y / PY / PN / N / NI |
| **Questions relating to baseline and time-varying confounding** | |  |
| 1.7. Did the authors use an appropriate analysis method that controlled for all the important confounding domains and for time-varying confounding? |  | NA / Y / PY / PN / N / NI |
| 1.8. **If Y/PY to 1.7**: Were confounding domains that were controlled for measured validly and reliably by the variables available in this study? |  | NA / Y / PY / PN / N / NI |
| **Risk of bias judgement** |  | Low / Moderate / Serious / Critical / NI |
| Optional: What is the predicted direction of bias due to confounding? |  | Favours experimental / Favours comparator / Unpredictable |

| **Bias in selection of participants into the study** | | |
| --- | --- | --- |
| 2.1. Was selection of participants into the study (or into the analysis) based on participant characteristics observed after the start of intervention?  **If N/PN to 2.1:** go to 2.4 |  | Y / PY / PN / N / NI |
| 2.2. **If Y/PY to 2.1**: Were the post-intervention variables that influenced selection likely to be associated with intervention?  2.3 **If Y/PY to 2.2**: Were the post-intervention variables that influenced selection likely to be influenced by the outcome or a cause of the outcome? |  | NA / Y / PY / PN / N / NI  NA / Y / PY / PN / N / NI |
| 2.4. Do start of follow-up and start of intervention coincide for most participants? |  | Y / PY / PN / N / NI |
| 2.5. **If Y/PY to 2.2 and 2.3, or N/PN to 2.4**: Were adjustment techniques used that are likely to correct for the presence of selection biases? |  | NA / Y / PY / PN / N / NI |
| **Risk of bias judgement** |  | Low / Moderate / Serious / Critical / NI |
| Optional: What is the predicted direction of bias due to selection of participants into the study? |  | Favours experimental / Favours comparator / Towards null /Away from null / Unpredictable |

| **Bias in classification of interventions** | | |
| --- | --- | --- |
| 3.1 Were intervention groups clearly defined? |  | Y / PY / PN / N / NI |
| 3.2 Was the information used to define intervention groups recorded at the start of the intervention? |  | Y / PY / PN / N / NI |
| 3.3 Could classification of intervention status have been affected by knowledge of the outcome or risk of the outcome? |  | Y / PY / PN / N / NI |
| **Risk of bias judgement** |  | Low / Moderate / Serious / Critical / NI |
| Optional: What is the predicted direction of bias due to classification of interventions? |  | Favours experimental / Favours comparator / Towards null /Away from null / Unpredictable |

| **Bias due to deviations from intended interventions** | | |
| --- | --- | --- |
| **If your aim for this study is to assess the effect of assignment to intervention, answer questions 4.1 and 4.2** | |  |
| 4.1. Were there deviations from the intended intervention beyond what would be expected in usual practice? |  | Y / PY / PN / N / NI |
| 4.2. **If Y/PY to 4.1**: Were these deviations from intended intervention unbalanced between groups *and* likely to have affected the outcome? |  | NA / Y / PY / PN / N / NI |
| **If your aim for this study is to assess the effect of starting and adhering to intervention, answer questions 4.3 to 4.6** | |  |
| 4.3. Were important co-interventions balanced across intervention groups? |  | Y / PY / PN / N / NI |
| 4.4. Was the intervention implemented successfully for most participants? |  | Y / PY / PN / N / NI |
| 4.5. Did study participants adhere to the assigned intervention regimen? |  | Y / PY / PN / N / NI |
| 4.6. **If N/PN to 4.3, 4.4 or 4.5**: Was an appropriate analysis used to estimate the effect of starting and adhering to the intervention? |  | NA / Y / PY / PN / N / NI |
| **Risk of bias judgement** |  | Low / Moderate / Serious / Critical / NI |
| Optional: What is the predicted direction of bias due to deviations from the intended interventions? |  | Favours experimental / Favours comparator / Towards null /Away from null / Unpredictable |

| **Bias due to missing data** | | |
| --- | --- | --- |
| 5.1 Were outcome data available for all, or nearly all, participants? |  | Y / PY / PN / N / NI |
| 5.2 Were participants excluded due to missing data on intervention status? |  | Y / PY / PN / N / NI |
| 5.3 Were participants excluded due to missing data on other variables needed for the analysis? |  | Y / PY / PN / N / NI |
| 5.4 **If PN/N to 5.1, or Y/PY to 5.2 or 5.3**: Are the proportion of participants and reasons for missing data similar across interventions? |  | NA / Y / PY / PN / N / NI |
| 5.5 **If PN/N to 5.1, or Y/PY to 5.2 or 5.3**: Is there evidence that results were robust to the presence of missing data? |  | NA / Y / PY / PN / N / NI |
| **Risk of bias judgement** |  | Low / Moderate / Serious / Critical / NI |
| Optional: What is the predicted direction of bias due to missing data? |  | Favours experimental / Favours comparator / Towards null /Away from null / Unpredictable |

| **Bias in measurement of outcomes** | | |
| --- | --- | --- |
| 6.1 Could the outcome measure have been influenced by knowledge of the intervention received? |  | Y / PY / PN / N / NI |
| 6.2 Were outcome assessors aware of the intervention received by study participants? |  | Y / PY / PN / N / NI |
| 6.3 Were the methods of outcome assessment comparable across intervention groups? |  | Y / PY / PN / N / NI |
| 6.4 Were any systematic errors in measurement of the outcome related to intervention received? |  | Y / PY / PN / N / NI |
| **Risk of bias judgement** |  | Low / Moderate / Serious / Critical / NI |
| Optional: What is the predicted direction of bias due to measurement of outcomes? |  | Favours experimental / Favours comparator / Towards null /Away from null / Unpredictable |

| **Bias in selection of the reported result** | | |
| --- | --- | --- |
| Is the reported effect estimate likely to be selected, on the basis of the results, from... |  |  |
| 7.1. ... multiple outcome *measurements* within the outcome domain? |  | Y / PY / PN / N / NI |
| 7.2 ... multiple *analyses* of the intervention-outcome relationship? |  | Y / PY / PN / N / NI |
| 7.3 ... different *subgroups*? |  | Y / PY / PN / N / NI |
| **Risk of bias judgement** |  | Low / Moderate / Serious / Critical / NI |
| Optional: What is the predicted direction of bias due to selection of the reported result? |  | Favours experimental / Favours comparator / Towards null /Away from null / Unpredictable |

| **Overall bias** | | |
| --- | --- | --- |
| **Risk of bias judgement** |  | Low / Moderate / Serious / Critical / NI |
| Optional: What is the overall predicted direction of bias for this outcome? |  | Favours experimental / Favours comparator / Towards null /Away from null / Unpredictable |

**Version 19 September 2016**


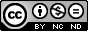


This work is licensed under a [Creative Commons Attribution-NonCommercial-NoDerivatives 4.0 International License](http://creativecommons.org/licenses/by-nc-nd/4.0/).

1. Study originally designed to be single arm; Natural comparison arose because administrative issues prohibited ability to begin medication administration prior to release from incarceration in the earlier stages of the trial; Groups early in the study were compared to those later in the study as post-release MAT administration (akin to a wait list control) and pre-release MAT administration, respectively; Time threats and group threats very likely; Adherence to the conditions was incredibly poor and substantially worse for the treatment group than the comparison group; The opposite was true for attrition for the six month follow up; Outcomes are all self-report and subject to recall bias and positive impression management. At least one domain was rated Critical Risk and therefore the study was excluded from synthesis. [↑](#footnote-ref-2)
